# Synthesis, Molecular Docking, In Vitro and In Vivo Studies of Novel Dimorpholinoquinazoline-Based Potential Inhibitors of PI3K/Akt/mTOR Pathway

**DOI:** 10.3390/ijms231810854

**Published:** 2022-09-17

**Authors:** Maria V. Zapevalova, Ekaterina S. Shchegravina, Irina P. Fonareva, Diana I. Salnikova, Danila V. Sorokin, Alexander M. Scherbakov, Alexander A. Maleev, Stanislav K. Ignatov, Ivan D. Grishin, Alexander N. Kuimov, Maryia V. Konovalova, Elena V. Svirshchevskaya, Alexey Yu. Fedorov

**Affiliations:** 1 Department of Organic Chemistry, Nizhny Novgorod State University, Gagarina Av. 23, 603950 Nizhny Novgorod, Russia; 2 N.D. Zelinsky Insitute of Organic Chemistry RAS, Leninsky Prospect 47, 119991 Moscow, Russia; 3 Department of Experimental Tumor Biology, Blokhin N.N. National Medical Research Center of Oncology, Kashirskoye Sh. 24, 115522 Moscow, Russia; 4 A.N. Belozersky Institute of Physico-Chemical Biology, M.V. Lomonosov Moscow State University, Leninskye Gory, House 1, Building 40, 119992 Moscow, Russia; 5 Department of Immunology, Shemyakin-Ovchinnikov Institute of Bioorganic Chemistry RAS, Miklukho-Maklaya 16/10, 117997 Moscow, Russia

**Keywords:** dimorpholinoquinazoline, cancer, PI3K/Akt/mTOR inhibitor, S6K, kinase inhibition activity

## Abstract

A (series) range of potential dimorpholinoquinazoline-based inhibitors of the *PI3K/Akt/mTOR cascade* was synthesized. Several compounds exhibited cytotoxicity towards a panel of cancer cell lines in the low and sub-micromolar range. Compound **7c** with the highest activity and moderate selectivity towards MCF7 cells which express the mutant type of PI3K was also tested for the ability to inhibit PI3K-(signaling pathway) downstream effectors and associated proteins. Compound **7c** inhibited the phosphorylation of Akt, mTOR, and S6K at 125–250 nM. It also triggered PARP1 cleavage, ROS production, and cell death via several mechanisms. Inhibition of PI3Kα was observed at a concentration of **7b** 50 µM and of **7c** 500 µM and higher, that can indicate minority PI3Kα as a target among other kinases in the titled cascade for **7c**. In vivo studies demonstrated an inhibition of tumor growth in the colorectal tumor model. According to the docking studies, the replacement of the triazine core in gedatolisib (**8**) by a quinazoline fragment, and incorporation of a (hetero)aromatic unit connected with the carbamide group via a flexible spacer, can result in more selective inhibition of the PI3Kα isoform.

## 1. Introduction

The PI3K-Akt-mTOR signaling pathway is one of the most frequently deregulated biochemical cascades in various types of cancer. This signaling network modulates several crucial processes including cell cycle, survival, metabolism, differentiation, and migration of cells [1,2,3,4]. It also influences processes in the tumor microenvironment such as interactions with immune cells, angiogenesis, and activation of the inflammatory response [5,6,7,8]. Strong interconnection of PI3K-Akt-mTOR with another oncogenic pathway, RAS-RAF-MEK-ERK, the ability to activate *MYC* oncogene [9,10,11], and “druggability” of the downstream effectors and the adaptor proteins in this cascade make the PI3K-Akt-mTOR pathway an attractive target for cancer treatment.

Enzymes PI3Ks can be divided into three classes according to their function and structural features [12]. Class I PI3Ks are heterodimers consisting of a p110 catalytic subunit and a p85 regulatory subunit. Four isoforms, namely, p110α, p110β, p110γ, and p110δ are encoded by *PIK3CA*, *PIK3CB*, *PIK3CG*, and *PIK3CD* genes, respectively [12]. Alterations in *PIK3CA*, especially “hotspot” mutations H1047R and E542K/E545K are strongly associated with tumor genesis [1]. Amplifications or the mutations in *PIK3CA* were detected in various types of tumors such as squamous lung cancer (about 47% cases), endometrial cancer (up to 53% cases), breast cancer (about 40% cases), ovarian, colorectal, and bladder cancer (more than 20% cases) [13].

In contrast to class I, PI3Ks kinases of class II have no obligatory regulatory subunit and act as monomers [14]. Class II PI3Ks participate in cell survival, migration, and differentiation; however, the lack of effective inhibitors of this class of PI3K evidences the differences in the architecture of these kinase binding pockets [14]. Class III PI3Ks are the most conservative among all PI3Ks—they demonstrate minimal changes from yeast to human [15]. The key functions of class III PI3Ks are the regulation of autophagy, lysosomal degradation, vesicular trafficking, and modulation of nutrient sensitivity within the mTOR signaling cascade [16,17].

Akt—a serine/threonine kinase (also known as protein kinase B), is a downstream effector in PI3K/Akt/mTOR cascade [18]. Hyperactivation of Akt may result in abnormal cell growth and division, as well as in the suppression of apoptosis. The high complexity of Akt involvement in multiple signal cascades offers an opportunity to simultaneously block several pathways essential for tumor genesis [19]. Although hyperactivation of Akt is frequently observed in different types of cancer [20,21], no FDA-approved Akt inhibitors were implemented in clinics up to date.

The mammalian target of rapamycin (mTOR) is another member of the PI3K-related kinase family. mTOR consists of two protein complexes: mTORC1 and mTORC2 [22]. Activation of mTORC1 is associated with cell growth and metabolism, while the functioning of mTORC2 is closely related to cell proliferation and mechanisms of survival [23]. The first generation of mTOR inhibitors was rapamycin and its structural analogs (“rapalogs”) which are allosteric inhibitors of mTORC1. The second generation of mTOR antagonists targets the ATP-binding site of the mTOR kinase domain. These drugs can inhibit both mTORC1 and mTORC2. Due to the structural similarity of the catalytic sites of mTOR and PI3K, some of the second-generation inhibitors, targeting both types of kinases, can overcome drug resistance and compensatory activation of PI3K, which typically takes place in response to the inhibition of mTORC1 [24].

**Figure 1:**
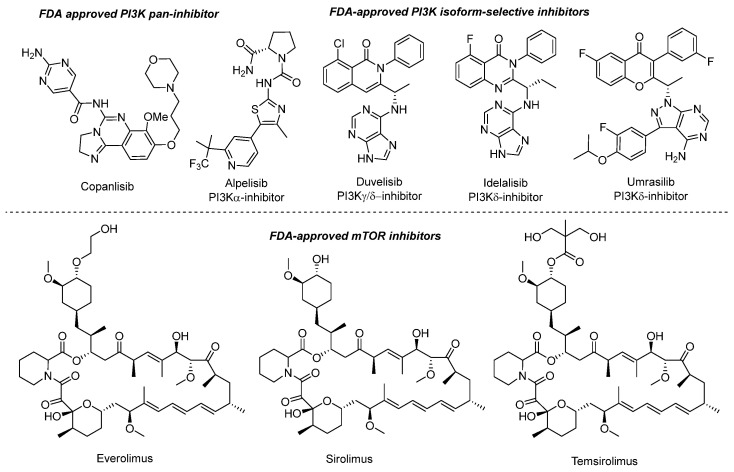
FDA-approved inhibitors of PI3K/Akt/mTOR pathway [2,30,31].

The development of pan-inhibitors of PI3K can be beneficial due to the frequent simultaneous appearance of multiple mutations in various enzyme isoforms in tumors [3]. To date, only one PI3K pan-inhibitor copanlisib (Figure 1) was approved by the FDA for the treatment of relapsed follicular lymphoma [25,26]. Unfortunately, most of the known inhibitors exhibit off-target effects, in particular, hyperglycemia and hyperinsulinemia, which require complex therapy [27]. Thus, elaboration of isoform-selective inhibitors is an important task [26,28,29] (Figure 1).

**Figure 2:**
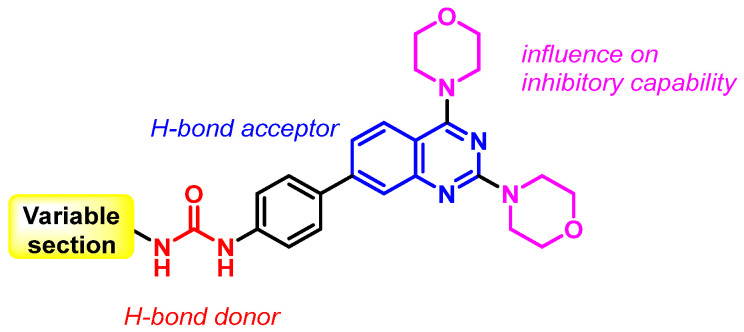
General structure of the target compounds.

Currently, a wide range of small molecules acting as PI3K-pathway inhibitors are under investigation, and some of them have entered clinical trials. Most of them mimic the interaction of the kinase with ATP, and therefore should contain a heterocyclic core. Based on the parent structure, several groups of PI3K inhibitors can be determined: triazines [32,33,34,35,36,37], pyrrolotriazines [38], azaindoles [39], isoindolinones [40,41], pyrimidines, thienopyrimidines, thiazolopyridines [42,43], quinolines, and the related compounds [44,45], quinazolines and aminoquinazolines [46,47,48,49]. This work is devoted to the design and synthesis of novel structural types of potential inhibitors of the PI3K/Akt/mTOR cascade. The general structure of the target molecules is presented in Figure 2. The quinazoline moiety and morpholine fragments are supposed to mimic the adenosine moiety in ATP and interact with the hinge region and the affinity pocket in PI3K [50]; the urea moiety is believed to form H-bonds with Asp810 or Lys802 [51]. The variable section contains aliphatic, aromatic, and heteroaromatic groups, adjusting the affinity, selectivity, or inhibitory activity of the synthesized compounds.

## 2. Results 

### 2.1. Chemistry

**Scheme 1:**
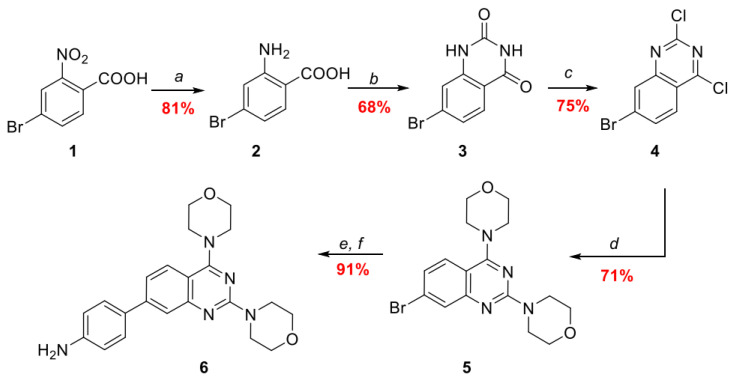
Synthesis of 7-aryl-2,4-bis(morpholino)quinazoline **6**. *Reagents and conditions: a*-SnCl_2_*2H_2_O, H_2_O-HCl (1:1), 90 °C; *b*-urea, 150 °C, no solvent; *c*-POCl_3_, Et_3_N, 95 °C; *d*-morpholine, DCM, 0 °C → rt; *e*-*N-Boc*-protected *p*-aminophenylpinacolborane, Pd(PPh_3_)_4_, Na_2_CO_3_, 1,4-dioxane-H_2_O, 100 °C; *f*-TFA, DCM, rt.

Condensation of urea with 4-bromoanthranilic acid (**2**), carried out without solvent, afforded quinazolinedione **3** in 68% yield (Scheme 1). It was then converted in two steps to bis(morpholino)quinazoline **5** in 55% overall yield. Finally, the 4-aminophenyl group was introduced into the quinazoline skeleton using the Suzuki cross-coupling reaction of 7-bromo-2,4-bis(morpholino)quinazoline **5** and *N-Boc*-protected *p*-aminophenylpinacolborane, followed by the deprotection under acidic conditions. The key intermediate **6** was obtained in 26% overall yield after 6 steps.

**Scheme 2:**
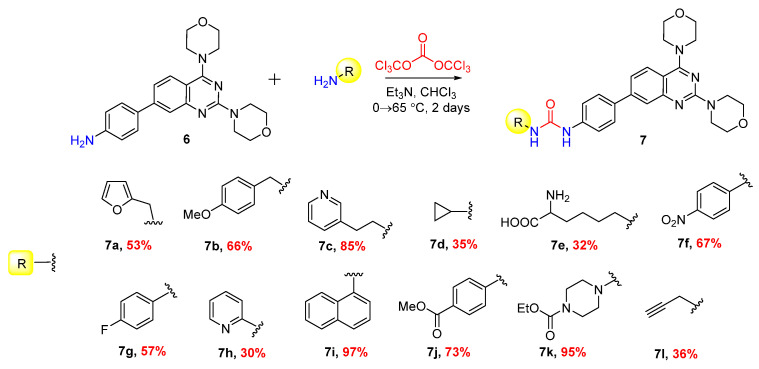
Synthesis of a small library of quinazoline-based asymmetric carbamides.

Aniline **6** was used as a building block for the synthesis of a library of unsymmetrical carbamides **7** (Scheme 2). Triphosgene served as a coupling reagent and a carbonyl source. Primary amines were used as a second component in the condensation.

Carbamides **7** contain various substituents at the urea fragments that are supposed to bind with the non-conserved region of the PI3K binding site. Predicted ADME parameters of target compounds are summarized in Table S1. All synthesized compounds entered preliminary biological investigations.

### 2.2. Biology

#### 2.2.1. Cytotoxicity of the Compounds **7**

**Figure 3:**
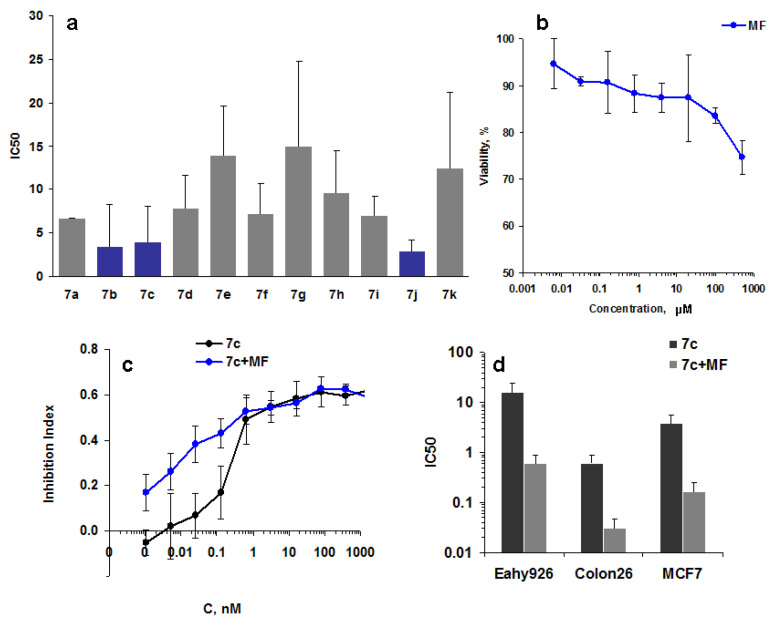
Characteristics of cytotoxicity. (**a**) Average IC_50_ for the compounds **7a**–**7k**. The most active compounds are shown in blue. (**b**) Synergistic effect of 1 mM metformin (MF) and 4 nM compound **7c** on the growth of MCF7 cells. (**c**) Effect of 300 µM MF on MCF7 proliferation. (**d**) IC_50_ for 7c and 7c + MF 300 µM for Eahy926, Colon26, and MCF7.

Antiproliferative activity of compounds **7** was estimated by the standard MTT assay in a panel of cancerous (MCF7 and MDA-MB-231) and noncancerous (macrophages J774, keratinocytes HaCaT, fibroblasts L929) cell lines. The results of cytotoxicity studies are summarized in Table 1 and Figure 3a.

Among 12 compounds synthesized, **7b**, **7c**, and **7j** demonstrated the highest cytotoxicity. However, all the compounds (with the exception of **7l)** had comparable activity in the low to moderate micromolar concentration range. Compound **7c**, bearing a pyridine moiety, exhibited submicromolar cytotoxicity towards MCF7 cells which express the mutant type of PI3K. Notably, the toxicity of **7c** for MDA-MB-231 cells possessing the wild type of PI3K was 5 times lower, indicating a moderate selectivity towards the cells with the mutant type of this target protein. The IC_50_ ratio of less active to active ones was only around 2–3 (Figure 3a) showing low selectivity. Of note, the PI3K pathway functions not only in cancerous but also in pseudonormal cells (Table 1).

The combination of PI3K inhibitors with other therapeutics such as metformin (first-line medication for treatment of type II diabetes) can overcome this drawback (Figure 3b).

Hyperglycemia is one of the most frequent side effects of pan- and isoform-selective PI3K inhibitors [52]. Drug-induced inhibition of PI3K reduces glucose uptake that stimulates insulin secretion. Long-term therapy with PI3K inhibitors can cause insulin resistance. Breakage of the insulin feedback might significantly improve the efficacy of PI3K inhibitors as anticancer remedies [53]. We have previously analyzed the antiproliferative potency of metformin (MF) against MCF7 breast cancer cells. Metformin induced a partial restoration of the cancer cell sensitivity to hormonal and target drugs. MF caused 50% suppression of MCF7 cell growth at a concentration of 5.8 mM [54]. Based on this fact, MF at the concentrations that had no significant antiproliferative effect was used in the combination with the lead compound. To analyze the possible synergetic effect of MF and the lead compound, we co-cultivated MCF7 cells with different doses of **7c** without or in the presence of 300 µM of MF. It resulted in a decrease in IC_50_ 20–25 times (Figure 3c,d)

#### 2.2.2. Inhibition of PI3K/Akt/mTOR Signaling Pathway

**Figure 4:**
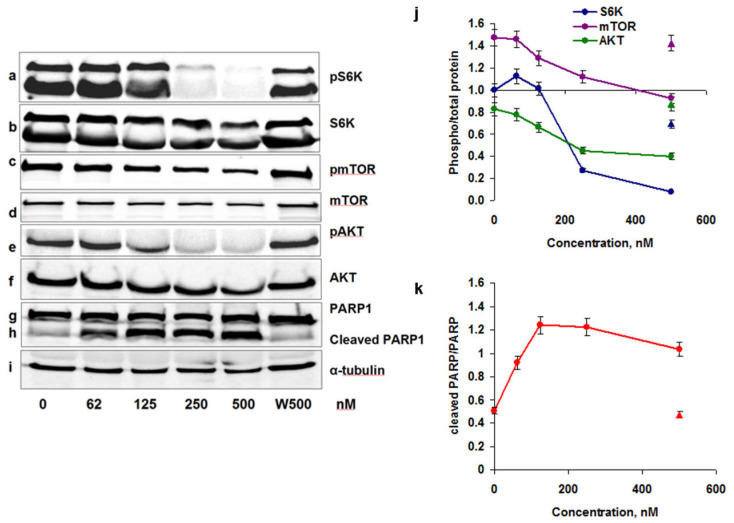
Compound **7c** suppresses the PI3K/Akt/mTOR signaling pathway. (**a**–**i**) MCF7 cells were treated with different concentrations of **7c** or 500 nM wortmannin (W) for 24 h. Cell lysates were immunoblotted with antibodies against PI3K/Akt/mTOR-related total or phosphorylated (p) proteins (**a**–**f**) and PARP1/cleaved PARP1 (**g**,**h**). (**i**) Control α-tubulin. (**j**,**k**) The ratios of p-proteins to total ones for compound **7c** (circles) or wortmannin (triangles) (**j**) and cleaved PARP1 to full PARP1 (**k**) as measured by ImageJ program from two independent experiments.

The effect of **7c** on the PI3K/Akt/mTOR signaling pathway was studied by immunoblotting of MCF7 cells in a comparison with wortmannin (covalent pan-PI3K inhibitor). The results are shown in Figure 4. Compound **7c** inhibited phosphorylation of PI3K/Akt/mTOR proteins at 250–500 nM (Figure 4a–g) to a higher extent than wortmannin. The ratios of phosphorylated to total proteins were calculated (Figure 4j).

These results show that compound **7c** as well as other compounds of type **7** strongly interfere with PI3K/Akt/mTOR signaling pathway. However, cell death was observed at 4–10 times higher concentrations, showing that DNA repair was involved. Poly(ADP-ribose)polymerase-1 (PARP1) binds to DNA, cleaves nicotinamide adenine dinucleotide, and generates ribosyl–ribosyl linkages that act as a signal for other DNA-repair enzymes and DNA base repair [55]. Evidently, the **7c** mechanism of action differs from that of wortmannin, as the latter neither affects PI3K/Akt/mTOR signaling pathway even at 500 nM, nor triggers DNA repair (Figure 4).

#### 2.2.3. PI3Kα Inhibition Assay

**Figure 5:**
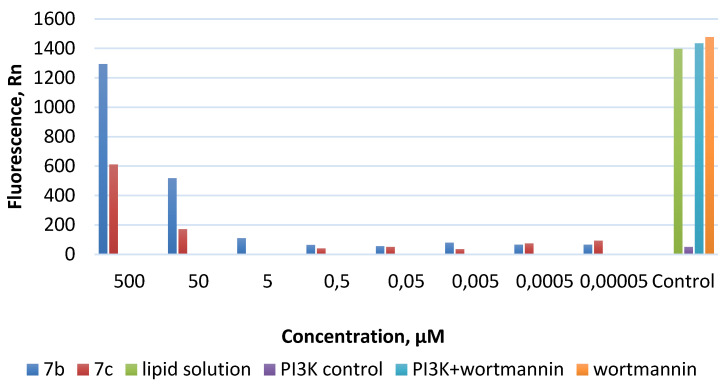
Inhibition of PI3Kα enzymatic activity with compounds **7b** (blue) or **7c** (red).

For two lead compounds **7b**,**c** an estimation of inhibition activity towards PI3Kα was performed. The assay of enzymatic activity was based on fluorescence resonance energy transfer (FRET) detection. The results of the experiment are summarized in Figure 5.

Both tested compounds inhibited PI3Kα. Apparently, compound **7b** (blue bars) was a more potent inhibitor of PI3Kα than its congener **7c** (red bars). IC_50_ for **7b** was about 50 µM, which is an order of magnitude lower than for **7c**. The solutions of single PI3Kα (violet bar), of PI3Kα mixed with its covalent inhibitor wortmannin (0.1 µM) (azure bar), and of single wortmannin (orange bar) were used as positive and negative controls. The green bar represents a signal of the reaction mixture with neither enzyme nor inhibitors. As well as the immunoblotting experiment (Figure 4), where the blockade of key downstream phosphorylation effectors of the PI3K cascade has already appeared in the presence of 125–250 nM **7c**, the data on inhibition of PI3K may suggest that this enzyme is a minor target of **7c** among the proteins of PI3K/Akt/mTOR cascade.

Taken collectively, the data indicate the ability of the synthesized quinazolines to affect PI3Kα activity; however, the determination of the extended range of potential targets and selectivity profiles towards different PI3K needs further investigations.

#### 2.2.4. Cell Cycle Analysis

To study the mechanisms of the cytotoxicity of compounds **7b,c,j**, cell cycle analysis was performed. The differences were found in a narrow window of concentrations between 5 μM and 10 μM. All cells died at concentrations higher than 10 μM, while no changes in the cell cycle were found below 1 μM concentration.

**Figure 6:**
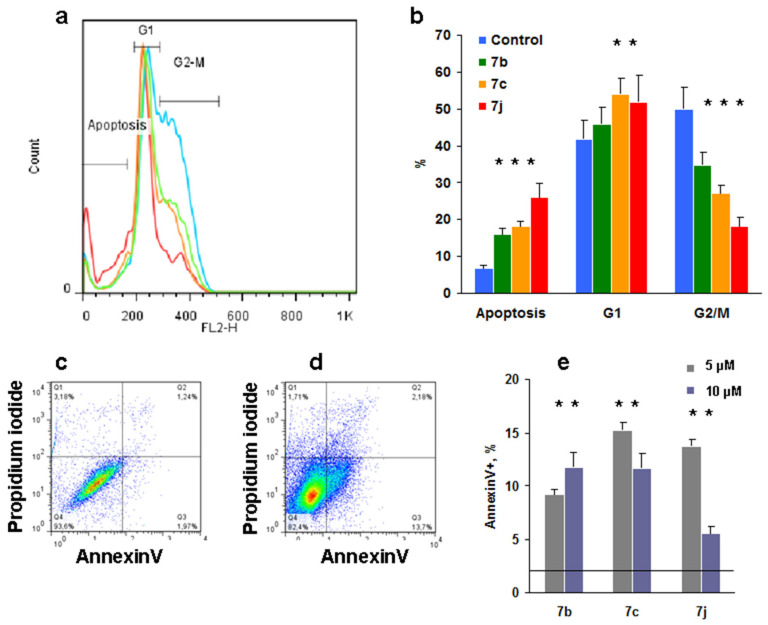
Mechanisms of cell death induced by the compounds **7.** (**a**,**b**) Representative data on the cell cycle of SkBr-3 cells after 24 h incubation with 10 μM of **7b** (green), **7c** (orange), and **7j** (red). Control cells are shown in blue color. (**c**–**e**) Apoptosis of SkBr-3 cells: (**c**) control cells, (**d**) cells incubated with **7c** for 24 h cells. (**e**) Apoptosis induction level for **7b**,**c**,**j** at 5 μM (gray) and 10 μM (violet). Apoptosis level in control cells is shown with a black line. Statistical significance is shown by asterisks.

All three compounds induced cell death of dividing cells, resulting in a decrease in the number of cells in the G2/M cell cycle phase, and an increase in the apoptotic cell population (Figure 6a,b). There was a minor difference in G0/G1 cell number. The effect of a well-known mTOR inhibitor rapamycin on the cell cycle was similar [56]. Apoptosis was demonstrated by cell staining with annexin V and propidium iodide (Figure 6c–e).

#### 2.2.5. Reactive Oxygen Species Production

**Figure 7:**
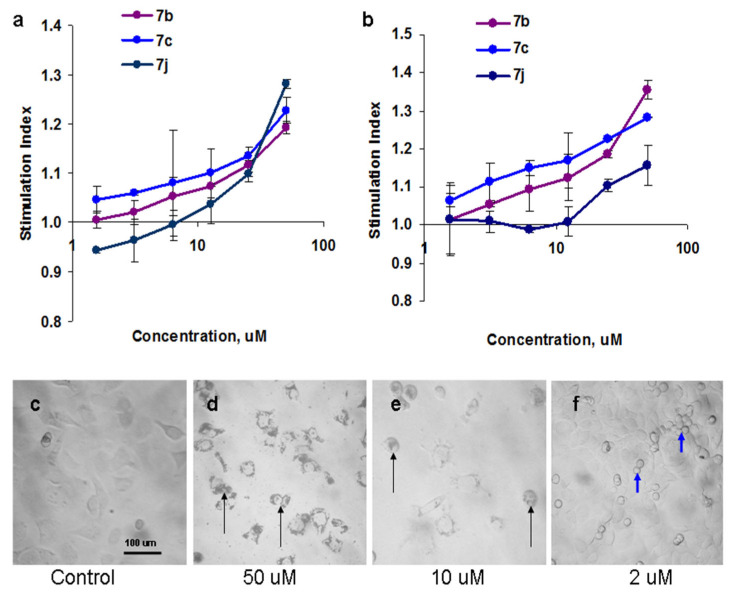
Reactive oxygen species production in the presence of compounds **7**. (**a**,**b**) SkBr-3 (**a**) and MCF7 (**b**) ROS production expressed as the ratios of intracellular ROS production by the stimulated cells to the control ones. (**c**–**f**) Microphotographs of SkBr-3 cells in the presence of compound **7c**. Black arrows show dying cells, blue arrows show unable to duplicate DNA cells.

**Figure 8:**
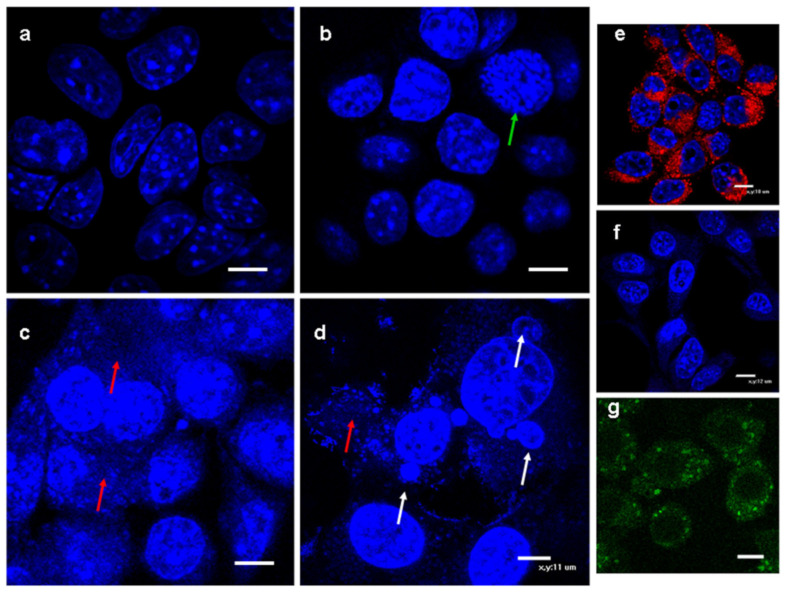
Biological effects induced by compound 7c in vitro. Confocal microphotographs of control (**a**,**e**) and compound **7c** treated (5 μM) (**b**,**c**,**f**,**g**) for 24 h MCF7 cells stained with Hoechst33342 (**a**–**f**), LysoTrackRed (**e**,**f**), and DCF (**g**). Release of nuclear material into cytoplasm (**c**,**d**, red arrows), disassembling of chromosomes (green arrow), large vesicles in the cytoplasm of the cells (white arrows), depolarization of lysosomes (**f**), and ROS production (**g**) show different types of cell death under compounds **7** influence. Scale bar is 10–15 μm.

Cell death can be induced by reactive oxygen species (ROS). We have analyzed ROS production using dichlorofluorescein diacetate (DCF), a specific dye, in which fluorescence is activated by ROS. At concentrations higher than 5 μM, compounds **7** stimulated ROS production (Figure 7a,b), although only moderately. ROS production was visualized by confocal microscopy (Figure 8g).

#### 2.2.6. Autophagy

The morphology of the dying cells under the treatment with 10–50 μM concentrations of compounds **7** differed significantly from a typical apoptosis picture, as no massive membrane blebbing was observed (Figure 7c,d). Seemingly, another type of programmed cell death (PCD) called autophagy, known to be induced by PI3K inhibitors such as apigenin, salidroside [57,58,59,60] as well as by the mTOR inhibitor rapamycin [61], took place. Generally, suppression of the PI3K/Akt/mTOR pathway leads to Atg1–Atg13 complex activation that results in autophagy initiation [62]. Autophagy is another form of PCD, where a section of the cytoplasm with organelles is surrounded by a membrane compartment and separated from the rest of the cytoplasm by two membranes. Autophagosomes fuse with lysosomes to form autophagolysosomes, in which organelles and the rest of the contents of autophagosomes are digested. Microphotographs of the cells treated with high-dose compounds **7** show dark zones in the cells (Figure 7d,e, black arrows), not found during apoptosis.

Analysis of the cell death induced by compounds **7** was performed also by confocal microscopy. We identified a release of nuclear materials into the cytoplasm (Figure 8c,d, red arrows); formation of autophagosomes (Figure 8d, white arrows); disassembling of chromosomes (Figure 8b, green arrow); depolarization of lysosomes (Figure 8e,f); and ROS production (Figure 8g).

**Figure 9:**
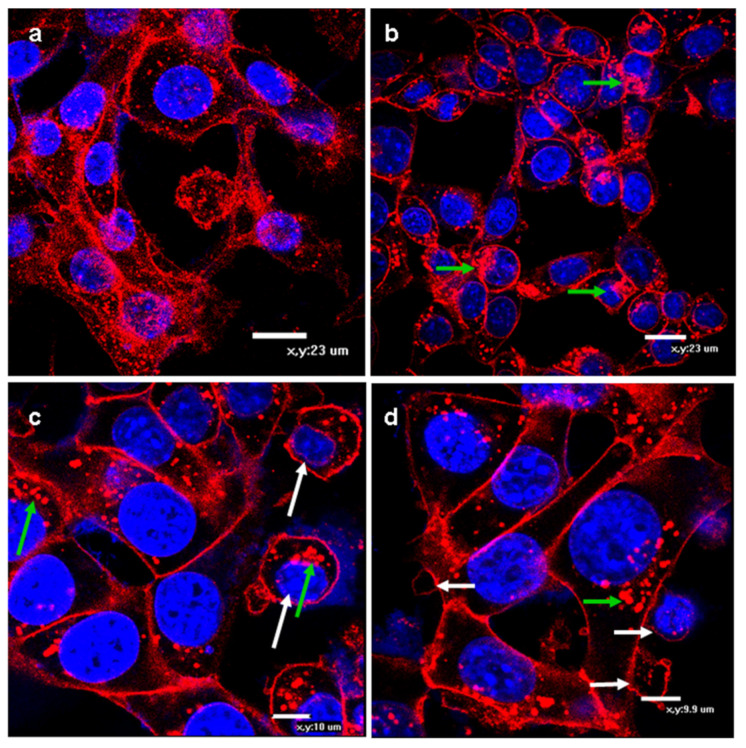
Confocal microphotographs of control (**a**) and the compound **7c** treated (5 μM) (**b**–**d**) for 24 h MCF7 cells stained with Hoechst33342 (blue) and WGA-AlexaFluor555 (red). Enlargement of organelles (green arrows) and autophagolysosome formation (white arrows) are found. Scale bar is 10–23 μm.

Changes in the lysosome membrane potential (Figure 8e,f) also confirmed targeting the PI3K/Akt/mTOR pathway by compounds **7**. A direct correlation between mTOR functioning, metabolic state of the cells, and lysosomal response was observed [63]. The depolarization of lysosomes depends on ATP-sensitive lysoNa_ATP_ channels, that are associated with mTOR [64]. Opening of lysoNa_ATP_ is observed after mTOR blocking, for example, by rapamycin and torin-1 [65]. The complete depolarization of lysosomes might be connected with autophagy induction. Additional staining of cell membranes with lectin WGA demonstrated organelle enlargement (Figure 9b–d, green arrows) and autophagosome formation (white arrows).

Thus, many PI3K inhibitors as well as the compounds of type **7** induce multiple types of PCD including apoptosis, autophagy, necrosis, possibly also ferroptosis, pyroptosis, oncosis, and others [66,67]. A more detailed study should find out the dominant type of PCD, induced by PI3K inhibitors.

#### 2.2.7. In Vivo Tumor Growth

**Figure 10:**
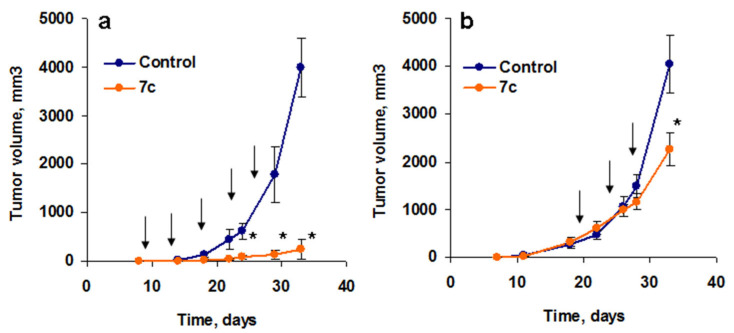
Effect of compound 7c on tumor growth. (**a**,**b**) BALB/c mice were s.c. inoculated with colorectal Colon-26 cells. Tumor growth in control and at early (**a**) or late (**b**) treatment with compound **7c**. Dates of the treatment with **7c** are shown with arrows, significant differences—with asterisks (*p* < 0.05). Data are shown as average ± SEM.

To estimate the antitumor potential of **7c**, mice were inoculated with colorectal murine tumor Colon26. Two protocols of the treatment were used to estimate the effect of the preparation: at early stages of tumor growth (group 1) and at late ones (group 2). The treatment was initiated in group 1 when the tumor volumes reached 2–8 mm^3^, at day 8 post cell inoculation. Compound **7c** is soluble only in DMSO, so intratumoral treatment was selected for the experiments [68]. The total volume of DMSO injected was 2.5 μL/per injection. To compensate for its effect, the control group was treated with the vehicle containing the same volume of DMSO in PBS. In this case, the effect of compound **7c** was significant post three injections (Figure 10a and Figure S1a,b). Group 2 was designed to monitor the effect on large tumors. Only three injections were given (Figure 10b). To this end, the effect of the treatment was less evident but still significant.

Visually large tumor treatment with compound **7c** resulted in tumor destruction (Figure S1c, see Supplementary Material for details); however, the total volume was still large. This can be a result of the environmental mass which does not proliferate but forms the volume of the tumor.

### 2.3. Docking Studies

Since the structure of PI3K was determined [69], a significant amount of research efforts was focused on the binding site architectures and pharmacophore models of possible inhibitors. It is known that the ATP-binding site of PI3K is highly conservative. It includes a hinge region, a specificity pocket, and an affinity pocket, which is not involved in the interactions with ATP but is surrounded by amino acid residues, common for all isoforms [28,50]. The development of specific inhibitors for the distinct isoforms is based on the analysis of two non-conserved clusters in the PI3K catalytic site, named regions 1 and 2 [70,71,72].

**Figure 11:**
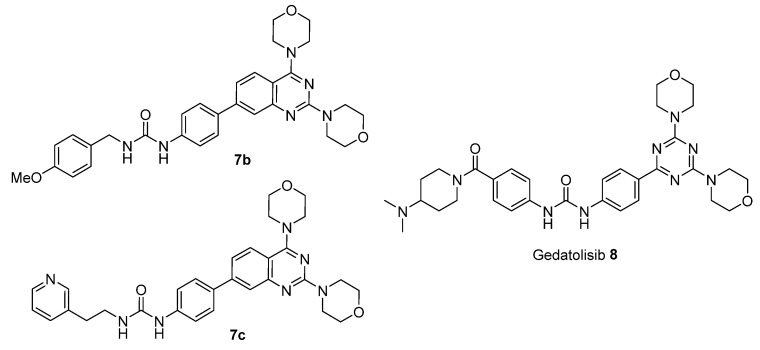
Structure of the molecules used for docking studies.

To gain an insight into the mechanism of interaction of the synthesized compounds with the target kinases, docking studies were carried out. Two active molecules **7b** and **7c** were chosen for the study. Gedatolisib (**8**), a highly active triazine-based PI3K inhibitor that has entered phase II clinical trials (NCT03698383, data for June 2022), was used as a reference compound (Figure 11).

**Figure 12:**
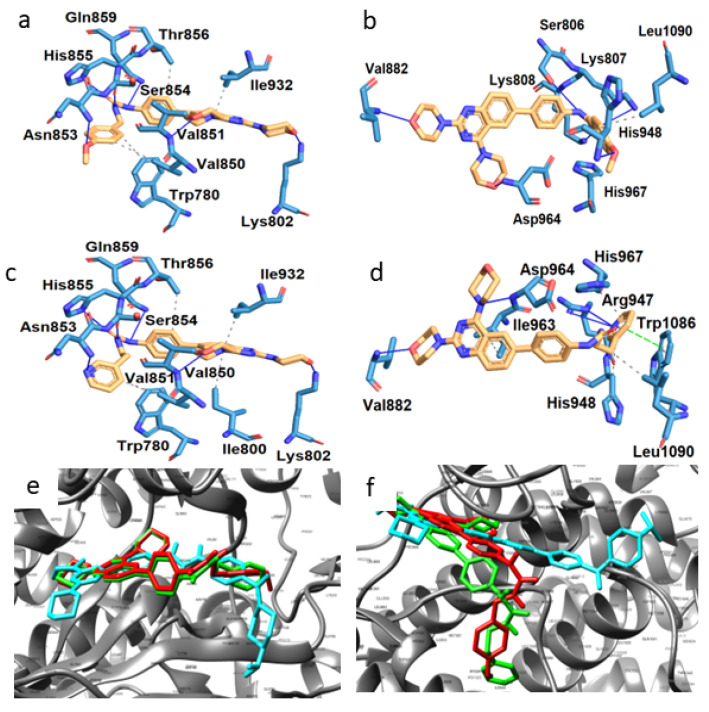
Docking of the compounds **7b** (**a**,**b**) and **7c** (**c**,**d**) into the catalytic site of PI3Kα (**a**,**c**) and PI3Kγ (**b**,**d**). The gray dotted line is hydrophobic interactions, the blue solid line is hydrogen bonds, the light green dotted line is parallel π-π stacking, dark green dashed—normal π-π stacking. yellow dotted line—salt bridge. Pictures 5E and 5F represent a superposition of **7b**, **7c** and Gedatolisib **8** in the catalytic site of PI3Kα (**e**) and PI3Kγ (**f**). Gedatolisib (**8**) is colored in blue, **7b**—red and **7c**—green. Visualization of the docking results (protein-ligand complexes) was carried out using the PLIP service. The superposition of ligands was visualized via UCSF Chimera program.

After the geometry optimization, docking of the titled compounds into the catalytic site of PI3K was carried out. The crystal structures of the p110a subunit of the PI3Kα (4TV3.pdb [73]) and PI3Kγ (7JWE.pdb [74]) proteins were used for the study. The amino acid environment of the molecules **7b**,**c**, and **8** in the catalytic site of proteins is shown in Figure 12. Other docking results (RMSD within the cluster, binding energies, inhibition constants) of **7b**,**c** and **8** in comparison with less active molecules **6** and **7a** are summarized in Table 2 (amino acids participating in H-bond formation are highlighted in bold).

According to the docking results, the quinazoline core did not form H-bonds (Figure 11a–d) but facilitated the proper accommodation of the ligands in the binding site. Moreover, the heterocyclic core participated in the hydrophobic interaction with Ile932 (Figure 12a,c), and Ile963 (Figure 12d). The pharmacophores, predominantly involved in H-bond formation, were the morpholine rings (H-bonds with Lys802, Asp964, Val882), the urea moiety (H-bonds with Ser854, Ser806, Lys808), and heteroatoms in the variable part of the molecule. The methoxy group in **7b** formed contacts with the Asn853 and His948 in the PI3Kα and PI3Kγ isoforms, respectively (Figure 12a,b). The nitrogen atom in the pyridyl moiety of **7c**, in turn, interacted with the Asn853 in the PI3Kα and with the Arg947 in the PI3Kγ, in addition to Trp1086, Leu1090 and His948, also participating in hydrophobic contacts and π stacking. The efficient bonding of **7c** may be caused by a flexible spacer, containing two carbon atoms. The lone electron pair at the nitrogen atom could be shared with the amino acid environment, in contrast to the lone electron pair at the oxygen of the furan ring in the less active compound **7a**.

In the case of PI3Kα, the accommodations of **7b** and **7c** in the catalytic site are very similar to each other, and share much in common with gedatolisib (**8**) (Figure 12e). For PI3Kγ, the positions of **7b** and **7c** are somewhat different. Moreover, the substituents at the carbamide fragments of **7b** and **7c** were significantly deflected from the distal part of gedatolisib **8** (Figure 12f). It is likely that such a difference in the binding mode as well as additional contacts with the amino acid residues for **7b** and **7c**, found for PI3Kα, enhance their selectivity for this isoform.

## 3. Materials and Methods

### 3.1. General Information 

All commercially available reagents were purchased from Aldrich, Fluka, Alfa Aesar, Abcr. ^1^H NMR and ^13^C NMR spectra were recorded on an Agilent DDR2 400 spectrometer at 25 °C. Chemical shifts (δ) are reported in ppm for the solution of the compound in DMSO-d_6_ with internal reference TMS and *J* values in Hertz. Atomic numeration is given only for NMR assignment. MALDI spectra were recorded on a Bruker Microflex LT spectrometer. Elemental analysis was performed using an Elementar (Vario Micro Cube) apparatus, all the compounds were found to have a purity of >95%. Column chromatography was performed using *Merck Kieselgel 60* (70–230 mesh). The purity of the compound, used in experiments in vivo, was analyzed by high-performance liquid chromatography (HPLC) (Knauer Smartline S2600) using a C18 column (Diasphere, 4.0 × 250 mm, 5 μM) at a flow rate of 0.8 mL/min, acquisition time 20 min, gradient mode from CH_3_CN/H_2_O (1:1, volume ratio) to CH_3_CN/H_2_O (95:5, volume ratio). The tested compound was >95% pure by HPLC analysis. All reactions were performed with commercially available reagents. Solvents were purified according to standard procedures. The petroleum ether used corresponds to the fraction 40–70 °C.

### 3.2. Synthesis of Compounds **2**–**6**

#### 3.2.1. Synthesis of 2-Amino-4-bromobenzoic Acid **2**

2-nitro-4-bromobenzoic acid (1.5g, 6.1 mmol, 1 equiv.) was dissolved in 23 mL of a mixture of water and hydrochloric acid (1:1), then SnCl_2_·2H_2_O (4.134 g, 18.3 mmol, 3 equiv.) was added. The mixture was stirred for 3 h at 90 °C. The reaction was cooled to room temperature and the precipitate was filtered off, washed with water, and dried to give an off-white solid. Yield 81% (1.067 g).

**M.p.** = 219–221 °C

**^1^H NMR** (400 MHz, DMSO-d_6_) δ 7.57 (d, J = 8.5 Hz, *C6-H*, 1H), 6.88 (d, J = 1.9 Hz, *C3-H*, 1H), 6.53 (dd, J = 8.5, 1.9 Hz, *C5-H*, 1H).

**^13^C NMR** (101 MHz, DMSO-d_6_) δ 169.36, 152.06, 132.70, 127.01, 117.99, 117.13, 78.42.

**Elemental analysis**: for C_7_H_6_BrNO_2_ calculated: C, 38.92; H, 2.80; found: C, 38.54; H, 2.89.

#### 3.2.2. Synthesis of 7-Bromoquinazoline-2,4(1H,3H)-dione **3**

2-amino-4-bromobenzoic acid **2** (500 mg, 2.31 mmol, 7 equiv.) and urea (1.448 g, 24.14 mmol, 73 equiv.) were stirred at 160 °C without solvent for 6 h. Then the reaction mixture was cooled to 90 °C and 15 mL of water was added while stirring for 5 min. The precipitate formed was filtered off and washed with water to yield a solid cake that was suspended in a solution of 0.5N NaOH (25mL) and heated to boil for 10 min. The solution was cooled and then acidified to pH 2 with concentrated HCl, and the solid was filtered off. After washing with water: methanol (1:1), the product was dried to give a pale-beige solid. Yield 68% (378 mg).

**M.p.** = 230 °C (decomposition)

**^1^H NMR** (400 MHz, DMSO-d_6_) δ 11.38 (s, *NH*, 1H), 11.29 (s, *NH*, 1H), 7.79 (d, J = 8.4 Hz, *C5-H*, 1H), 7.38 (d, J = 1.7 Hz, *C8-H*, 1H), 7.34 (dd, J = 8.4, 1.8 Hz, *C6-H*, 1H).

**^13^C NMR** (101 MHz, DMSO-d_6_) δ 162.66, 150.52, 142.46, 129.41, 128.67, 125.70, 118.13, 114.05.

**Elemental analysis**: for C_8_H_5_BrN_2_O_2_ calculated: C, 39.86; H, 2.09; found: C, 39.72; H, 2.20.

#### 3.2.3. Synthesis of 7-Bromo-2,4-dichloroquinazoline **4**

7-bromoquinazoline-2,4(1H,3H)-dione (300 mg, 1.24 mmol, 3 equiv.), triethylamine (210 mg, 0.3 mL, 2.07 mmol, 5 equiv.) and POCl_3_ (1.720 g, 1.05 mL, 11.2 mmol, 27 equiv.) were refluxed for 7 h. Then the mixture was cooled, and crushed ice was added. The reaction mixture was then stirred for 1 h at 0–5 °C. The solid product was filtered off, washed with water and dried to give a yellow solid. Yield 75% (259 mg).

**M.p.** = 189−191 °C

**^1^H NMR** (400 MHz, DMSO-d_6_) δ 7.83 (d, J = 8.8 Hz, *C5-H*, 1H), 7.70 (d, J = 1.9 Hz, *C8-H*, 1H), 7.40 (dd, J = 8.8, 1.9 Hz, *C6-H*, 1H).

**^13^C NMR** (101 MHz, DMSO-d_6_) δ 161.31, 159.85, 149.07, 128.59, 128.26, 126.74, 122.99, 114.33.

**Elemental analysis**: for C_8_H_3_BrCl_2_N_2_ calculated: C, 34.57; H, 1.09; found: C, 34.79; H, 1.31.

#### 3.2.4. Synthesis of 4,4’-(7-Bromoquinazoline-2,4-diyl)dimorpholine **5**

7-bromo-2,4-dichloroquinazoline (250 mg, 0.9 mmol, 1 equiv.) was dissolved in 12 mL of dichloromethane, then morpholine (313 mg, 0.3 mL, 3.6 mmol, 4 equiv.) was added. The reaction was carried out for 1 h at 0 °C, then 23 h at room temperature. After the completeness of the reaction, the precipitate was filtered off and the filtrate was concentrated under reduced pressure. The product was purified by column chromatography to give a beige solid, yield 71% (242 mg). Eluent: petroleum ether-ethyl acetate (6:1, then 2:1).

**M.p.** = 136−138 °C

**^1^H NMR** (400 MHz, DMSO-d_6_) δ 7.72 (d, J = 8.8 Hz, *C5-H*, 1H), 7.56 (d, J = 2.0 Hz, *C8-H*, 1H), 7.21 (dd, J = 8.8, 2.1 Hz, *C6-H*, 1H), 3.77–3.74 (m, *N-CH_2_ (morpholine)*,8H), 3.67–3.64 (m, *O-CH_2_* (*morpholine*),4H), 3.63–3.60 (m, *O-CH_2_* (*morpholine*),4H).

**^13^C NMR** (101 MHz, DMSO-d_6_) δ 164.67, 163.99, 128.97, 128.52, 127.93, 127.43, 112.95, 110.09, 66.06, 65.85, 49.74, 49.30.

**MALDI** (positive mode, no matrix): 379.24 (M+H) 100%.

**Elemental analysis**: for C_16_H_19_BrN_4_O_2_ calculated: C, 50.67; H, 5.05; found: C, 50.42; H, 5.09;

#### 3.2.5. Synthesis of Tert-butyl (4-(2,4-dimorpholinoquinazolin-7-yl)phenyl)carbamate

4,4’-(7-bromoquinazoline-2,4-diyl)dimorpholine **5** (240 mg, 0.63 mmol, 1 equiv.), *N*-*Boc*-aminophenylboronic acid pinacol ester (222 mg, 0.70 mmol, 1.1 equiv.), sodium carbonate (201 mg, 1.90 mmol, 3 equiv.) were placed into a Schlenk flask, the flask was filled with argon. The mixture of 17 mL of dioxane and 0.3 mL of water was added followed by Pd(PPh_3_)_4_ (93 mg, 0.13 mmol, 0.2 equiv.). The reaction was carried out for 24 h at 90 °C. Then the mixture was poured into distilled water (50 mL) and extracted with AcOEt (3 × 70 mL). The combined organic layer was dried over Na_2_SO_4_ and concentrated under reduced pressure. The product was purified by column chromatography to give a beige solid, yield 93% (289 mg). Eluent: petroleum ether-ethyl acetate (2:1).

**M.p.** = 148−150 °C

**^1^H NMR** (400 MHz, DMSO-d_6_) δ 9.51 (s, *NH*, 1H), 7.83 (d, J = 8.7 Hz, *C5-H*, 1H), 7.69 (d, J = 8.8 Hz, *C14-H*, *C10-H*, 2H), 7.61 (d, J = 1.9 Hz, *C8-H*, 1H), 7.58 (d, J = 8.8 Hz, *C11-H*, *C13-H*, 2H), 7.41 (dd, J = 8.7, 1.9 Hz, *C6-H*, 1H), 3.81–3.74 (m, *N-CH_2_* (*morpholine*), 8H), 3.70–3.65 (m, *O-CH_2_* (*morpholine*), 4H), 3.65–3.60 (m, *O-CH_2_* (*morpholine*), 4H), 1.49 (s, *Boc*, 9H).

**^13^C NMR** (101 MHz, DMSO-d_6_) δ 164.78, 157.91, 154.20, 152.71, 143.64, 139.80, 132.54, 127.20, 125.92, 121.97, 119.42, 118.40, 110.04, 79.24, 66.14, 65.94, 49.84, 44.14, 28.12.

**MALDI** (positive mode, no matrix): 490.59 (M^+^) 100%

**Elemental analysis**: for C_27_H_33_N_5_O_4_ calculated: C, 65.97; H, 6.77; found: C, 66.16; H, 6.65.

#### 3.2.6. Synthesis of 4-(2,4-Dimorpholinoquinazolin-7-yl)aniline **6**

Tert-butyl (4-(2,4-dimorpholinoquinazolin-7-yl)phenyl)carbamate (289 mg, 0.59 mmol, 1 equiv.) was dissolved in 26 mL of a mixture of trifluoroacetic acid and dichloromethane (7:27). The reaction was carried out for 1 h at room temperature. The mixture was then neutralized with sodium bicarbonate until pH = 8 and extracted with AcOEt (3 × 50 mL). The extract was dried over Na_2_SO_4_ and concentrated under reduced pressure. The product was purified by column chromatography to give a yellow solid, yield 98% (225 mg). Eluent: petroleum ether-ethyl acetate (1:3).

**M.p.** = 191–193 °C.

**^1^H NMR** (400 MHz, DMSO-d_6_) δ 7.77 (d, J = 8.7 Hz, *C5-H*, 1H), 7.51 (d, J = 1.8 Hz, *C8-H*, 1H), 7.48 (d, J = 8.6 Hz, *C10-H*, *C14-H*, 2H), 7.36 (dd, J = 8.6, 1.9 Hz, *C8-H*, 1H), 6.66 (d, J = 8.6 Hz, *C11-H*, *C13-H*, 2H), 5.38 (s, *NH_2_*, 2H), 3.80–3.74 (m, *N-CH_2_* (*morpholine*), 8H), 3.69–3.64 (m, *O-CH_2_* (*morpholine*),4H), 3.63–3.58 (m, *O-CH_2_* (*morpholine*),4H).

**^13^C NMR** (101 MHz, DMSO-d_6_) δ 164.75, 157.80, 149.28, 144.58, 127.56, 125.91, 125.69, 120.41, 119.14, 114.17, 112.88, 109.31, 66.14, 65.97, 49.86, 44.17.

**MALDI** (DCTB, pos. mode): 390.29 (M^+^) 86%

**Elemental analysis**: for C_22_H_25_N_5_O_2_ calculated: C, 67.50; H, 6.44; found: C, 67.50; H, 6.44.

### 3.3. General Procedure for Synthesis of Compounds **7a**–**l**

Triphosgene (14 mg, 0.28 mmol, 0.37 equiv.) was placed into a Schlenk flask, and the flask was filled with argon. Chloroform (1 mL) was added and the mixture was cooled to 0 °C. The solution of 4-(2,4-dimorpholinoquinazolin-7-yl)aniline (50 mg, 0.128 mmol, 1 equiv.) and triethylamine (28.4 mg, 40 μL, 0.28 mmol, 2.2 equiv.) in 1 mL of chloroform was added to the mixture. The reaction was carried out for 30 min at 0 °C, then 20 h at 65 °C. After that, the corresponding amine (1 equiv.) was added to the mixture in one portion. The reaction was carried out for an additional 24 h at 65 °C. After the completeness of the reaction, the volatiles were removed under reduced pressure and the product was isolated using column chromatography.

#### 3.3.1. 1-(4-(2,4-Dimorpholinoquinazolin-7-yl)phenyl)-3-(furan-2-ylmethyl)urea **7a**

Amine: furan-2-ylmethanamine (12.4 mg, 0.128 mmol, 1 equiv.)

Eluent: petroleum ether-ethyl acetate (1:1). Pale yellow solid, yield 53%.

**M.p**. = 184–186 °C.

**^1^H NMR** (400 MHz, DMSO-d_6_) δ 8.70 (s, *NH*, 1H), 7.83 (d, J = 8.7 Hz, *C5-H*, 1H), 7.68 (d, J = 8.7 Hz, *C10-H*, *C14-H*, 2H), 7.60 (d, J = 1.9 Hz, *C8-H*, 1H), 7.59 (dd, J = 1.8, 0.7 Hz, *C17-H*, 1H), 7.52 (d, J = 8.8 Hz, *C4-H*, *C5-H*, 2H), 7.41 (dd, J = 8.7, 1.9 Hz, *C6-H*, 1H), 6.61 (t, J = 5.7 Hz, *NH*, 1H), 6.40 (dd, J = 3.1, 1.9 Hz, C18-H, 1H), 6.27 (dd, J = 3.2, 0.5 Hz, C19-H, 1H), 4.31 (d, J = 5.7 Hz, CH_2_, 2H), 3.82–3.74 (m, *N-CH_2_* (*morpholine*), 8H), 3.70–3.60 (m, *O-CH_2_* (*morpholine*), 8H).

**^13^C NMR** (101 MHz, DMSO-d_6_) δ 164.77, 157.89, 154.77, 154.19, 153.02, 143.75, 142.04, 140.64, 131.69, 127.25, 125.90, 121.77, 119.37, 118.00, 110.45, 109.96, 106.52, 66.13, 65.94, 49.84, 44.14, 36.13.

**MALDI** (no matrix. pos. mode): 514.0 (M^+^) 100%

**Elemental analysis**: for C_28_H_30_N_6_O_4_ calculated: C, 65.36; H, 5.88; found: C, 65.50; H, 5.97.

#### 3.3.2. 1-(4-(2,4-Dimorpholinoquinazolin-7-yl)phenyl)-3-(4-methoxybenzyl)urea **7b**

Amine: (4-methoxyphenyl)methanamine (17.5 mg, 17 μL, 0.128 mmol, 1 equiv.)

Eluent: petroleum ether-ethyl acetate (1:1). Yellowish solid, yield 66%.

**M.p.** = 210–212 °C.

**^1^H NMR** (400 MHz, DMSO-d_6_) δ 8.69 (s, *NH*, 1H), 7.81 (d, J = 8.7 Hz, *C5-H*,1H), 7.67 (d, J = 8.8 Hz, *C10-H*, *C14-H*, 2H), 7.60 (d, J = 1.8 Hz, *C8-H*, 1H), 7.53 (d, J = 8.8 Hz, *C11-H*, *C13-H*, 2H), 7.41 (dd, J = 8.7, 1.8 Hz, *C6-H*, 1H), 7.27–7.22 (m, *C17-H*, *C21-H*, 2H), 6.92–6.88 (m, *C18-H*, *C20-H*, 2H), 6.59 (t, J = 5.9 Hz, *NH*, 1H), 4.24 (d, J = 5.8 Hz, *C15-H*, 2H), 3.81–3.75 (m, *N-CH_2_* (*morpholine*), 8H), 3.73 (s, *O-CH_3_* 3H), 3.70–3.60 (m, *O-CH_2_* (*morpholine*), 8H).

**^13^C NMR** (101 MHz, DMSO-d_6_) δ 164.79, 158.21, 157.92, 155.04, 154.22, 143.79, 140.82, 132.13, 131.56, 128.54, 127.24, 125.91, 121.76, 119.37, 117.97, 113.73, 109.96, 66.15, 65.96, 55.07, 49.85, 44.15, 42.26.

**MALDI** (DCTB, pos. mode): 555.1 (M+H) 100%

**Elemental analysis**: for C_31_H_34_N_6_O_4_ calculated: C, 67.13; H, 6.18; found: C, 67.31; H, 6.28.

#### 3.3.3. 1-(4-(2,4-Dimorpholinoquinazolin-7-yl)phenyl)-3-(2-(pyridin-3-yl)ethyl)urea **7c**

Amine: 2-(pyridin-3-yl)ethan-1-amine (15.6 mg, 0.128 mmol, 1 equiv.)

Eluent: ethyl acetate-acetone (1:1). Yellow solid, yield 85%.

**M.p**. = 193–195 °C.

**^1^H NMR** (400 MHz, DMSO-d_6_) δ 8.66 (s, *NH*, 1H), 8.47 (d, *J* = 1.8 Hz, C21-H, 1H), 8.44 (dd, *J* = 4.7, 1.7 Hz, C20-H, 1H), 7.83 (d, *J* = 8.7 Hz, C5-H, 1H), 7.70–7.68 (m, C19-H, 1H), 7.66 (d, *J* = 8.8 Hz, C10-H, C14-H, 2H), 7.61 (d, *J* = 1.7 Hz, C8-H, 1H), 7.51 (d, *J* = 8.8 Hz, C11-H, C13-H, 2H), 7.41 (dd, *J* = 8.6, 1.8 Hz, C6-H, 1H), 7.34 (dd, *J* = 7.7, 5.1 Hz, C18-H, 1H), 6.23 (t, *J* = 5.7 Hz, NH, 1H), 3.78 (dd, *J* = 9.5, 6.3 Hz, *N-CH_2_* (*morpholine*), 8H), 3.71–3.60 (m, *O-CH_2_* (*morpholine*), 8H), 3.40–3.37 (m, *J* = 6.0 Hz, C15-H, 2H), 2.79 (t, *J* = 7.0 Hz, *C16-H*, 2H).

**^13^C NMR** (101 MHz, DMSO-d_6_) δ 164.67, 157.65, 155.00, 153.88, 149.91, 147.42, 143.85, 140.84, 136.23, 135.01, 131.45, 127.22, 125.95, 123.48, 121.52, 119.44, 117.91, 109.87, 66.12, 65.95, 49.82, 44.17, 32.83.

**MALDI** (DCTB, pos. mode): 540.3 (M+H) 100%, 562.3 (M+Na) 1.2%

**Elemental analysis**: for C_30_H_33_N_7_O_3_ calculated: C, 66.77; H, 6.16; found: C, 66.58; H, 6.22.

**HPLC analysis**: purity 95,89%, retention time: 5467 min.

#### 3.3.4. 1-Cyclopropyl-3-(4-(2,4-dimorpholinoquinazolin-7-yl)phenyl)urea **7d**

Amine: cyclopropanamine (7.3 mg, 0.128 mmol, 1 equiv.)

Eluent: petroleum ether-ethyl acetate (1:1). Pale beige solid, yield 35%.

**M.p**. = 83–85 °C.

**^1^H NMR** (400 MHz, DMSO-d_6_) δ 8.59 (s, *NH*, 1H), 7.82 (d, *J* = 8.7 Hz, C5-H, 1H), 7.67 (d, *J* = 8.8 Hz, C10-H, C14-H, 2H), 7.65–7.62 (m, *NH*, 1H), 7.60 (d, *J* = 1.8 Hz, C8-H, 1H), 7.53 (d, *J* = 8.8 Hz, C11-H, C13-H, 2H), 7.41 (dd, *J* = 8.7, 1.8 Hz, C6-H, 1H), 3.83–3.74 (m, *N-CH_2_* (*morpholine*), 8H), 3.70–3.60 (m, *O-CH_2_* (*morpholine*), 8H), 2.56 (ddd, *J* = 10.2, 6.9, 3.4 Hz, C14-H, 1H), 1.25–1.22 (m, C16-H, 2H), 0.87–0.81 (m, C17-H, 2H).

**^13^C NMR** (101 MHz, DMSO-d_6_) δ 164.78, 157.90, 155.90, 154.22, 143.80, 140.74, 131.54, 127.17, 125.89, 121.74, 119.38, 118.07, 109.94, 66.14, 65.95, 49.84, 44.14, 22.40, 6.40, 5.59.

**MALDI** (no matrix. pos. mode): 475.4 (M+H) 100%, 497.5 (M+Na) 1%.

**Elemental analysis**: for C_26_H_30_N_6_O_3_ calculated: C, 65.80; H, 6.37; found: C, 65.61; H, 6.33.

#### 3.3.5. N6-((4-(2,4-Dimorpholinoquinazolin-7-yl)phenyl)carbamoyl)lysine **7e**

Amine: *rac*-lysine (7 mg, 0.128 mmol, 1 equiv.)

Eluent: petroleum ether-ethyl acetate (1:2). Pale beige solid, yield 32%.

**M.p.** = 89–91 °C.

**^1^H NMR** (400 MHz, DMSO-d_6_) δ 9.84 (s, *NH*, 1H), 9.79 (s, *NH*, 1H), 8.09 (d, *J* = 8.8 Hz, C5-H, 1H), 7.95 (d, *J* = 1.9 Hz, C8-H, 1H), 7.81–7.79 (m, C10-H, C14-H, 2H), 7.74–7.70 (m, C11-H, C13-H, 2H), 7.42 (dd, *J* = 8.8, 1.9 Hz, C6-H, 1H), 3.87–3.85 (m, NH_2_, C19-H, 3H), 3.81–3.75 (m, *N-CH_2_* (*morpholine*), *C15-H*, 10H), 3.69–3.66 (m, *O-CH_2_* (*morpholine*), 4H), 3.65–3.62 (m, *O-CH_2_* (*morpholine*), 4H), 1.26 (td, *J* = 7.1, 1.3 Hz, C16-H, C17-H, C18-H, 6H).

**^13^C NMR** (101 MHz, DMSO-d_6_) δ 164.70, 164.20, 155.68, 153.46, 144.52, 140.06, 139.50, 131.69, 127.64, 124.01, 123.06, 121.37, 118.48, 112.67, 66.12, 65.92, 60.32, 49.82, 49.36, 44.14, 30.68, 24.77, 22.07

**MALDI** (no matrix. pos. mode): 464.0 (100%), 488.1 (9%).

**Elemental analysis**: for C_29_H_37_N_7_O_5_ calculated: C, 61.80; H, 6.62; found: C, 61.67; H, 6.72.

#### 3.3.6. 1-(4-(2,4-Dimorpholinoquinazolin-7-yl)phenyl)-3-(4-nitrophenyl)urea **7f**

Amine: 4-nitroaniline (17.7 mg, 0.128 mmol, 1 equiv.)

Eluent: petroleum ether-ethyl acetate (1:2). Yellow solid, yield 67%.

**M.p**. = 240–242 °C.

**^1^H NMR** (400 MHz, DMSO-d_6_) δ 10.87 (s, *NH*, 1H), 10.33 (s, *NH*, 1H), 8.20 (d, J = 9.2 Hz, C17-H, C19-H, 2H), 7.84 (d, J = 8.5 Hz, *C5-H*, 1H), 7.74 (m, *C11-H*, *C13-H*, *C16-H*, *C20-H*, 4H), 7.62 (m, C10-H, C14-H, C8-H, 3H), 7.44 (dd, J = 8.6, 1.6 Hz, *C6-H*, 1H), 3.83–3.75 (m, *N-CH_2_* (*morpholine*), 8H), 3.71–3.60 (m, *O-CH_2_* (*morpholine*), 8H).

**^13^C NMR** (101 MHz, DMSO-d_6_) δ 164.79, 157.93, 154.23, 152.24, 150.75, 146.63, 140.81, 139.63, 134.04, 132.67, 127.44, 127.36, 125.95, 125.27, 121.84, 119.43, 118.37, 117.01, 110.07, 66.15, 65.96, 49.86, 44.16.

**MALDI** (DCTB, pos. mode): 537.0 100%, 556.2 (M+H) 36%.

**Elemental analysis**: for C_29_H_29_N_7_O_5_ calculated: C, 62.69; H, 5.26; found: C, 62.82; H, 5.37.

#### 3.3.7. 1-(4-(2,4-Dimorpholinoquinazolin-7-yl)phenyl)-3-(4-fluorophenyl)urea **7g**

Amine: 4-fluoroaniline (14 mg, 12 μL, 0.128 mmol, 1 equiv.)

Eluent for column chromatography: petroleum ether-ethyl acetate (1:1). Yellow solid, yield 57%.

**M.p.** = 190–192 °C.

**^1^H NMR** (400 MHz, DMSO-d_6_) δ 8.85 (s, *NH*, 1H), 8.77 (s, *NH*, 1H), 7.84 (d, J = 8.7 Hz, *C5-H*, 1H), 7.73 (d, J = 8.8 Hz, *C10-H*, *C14-H*, 2H), 7.63 (d, J = 1.8 Hz, *C8-H*, 1H), 7.58 (d, J = 8.8 Hz, *C11-H*, *C13-H*, 2H), 7.51–7.45 (m, *C17-H*, *C19-H*, 2H), 7.43 (dd, J = 8.7, 1.9 Hz, *C6-H*, 1H), 7.17–7.09 (m, *C16-H*, *C20-H*, 2H), 3.82–3.75 (m, *N-CH_2_* (*morpholine*), 8H), 3.70–3.61 (m, *O-CH_2_* (*morpholine*), 8H).

**^13^C NMR** (101 MHz, DMSO-d_6_) δ 164.75, 158.56, 157.87 (d, *J_C-F_* = 0.9 Hz), 156.19, 152.52, 143.69, 140.00, 135.93 (d, *J_C-F_* = 2.4 Hz), 132.33, 127.35, 125.96, 121.89, 120.05 (d, *J_C-F_* = 7.7 Hz), 119.43, 118.52, 115.40 (d, *J_C-F_* = 22.2 Hz), 110.02, 66.13, 65.95, 49.83, 44.15.

**^19^F NMR** (376 MHz, DMSO-d_6_) δ -121.40 (ddd, J = 13.7, 8.8, 5.0 Hz).

**MALDI** (DCTB, pos. mode): 529.1 (M+H) 100%, 527.1 (M^+^) 12%

**Elemental analysis**: for C_29_H_29_FN_6_O_3_ calculated: C, 65.90; H, 5.53; found: C, 65.69; H, 5.48.

#### 3.3.8. 1-(4-(2,4-Dimorpholinoquinazolin-7-yl)phenyl)-3-(pyridin-2-yl)urea **7h**

Amine: pyridin-2-amine (12 mg, 0.128 mmol, 1 equiv.)

Eluent: petroleum ether-ethyl acetate (1:2). Brown solid, yield 30%.

**M.p.** = 210–212 °C.

**^1^H NMR** (400 MHz, DMSO-d_6_) δ 10.70 (s, *NH*, 1H), 9.52 (s, *NH*, 1H), 8.31 (d, *J* = 5.9 Hz, C19-H, 1H), 8.00 (dd, *J* = 6.8, 1.2 Hz, C16-H, 1H), 7.76 (d, *J* = 8.6 Hz, *C10-H*, *C14-H*, 2H), 7.67 (d, *J* = 8.7 Hz, *C11-H*, *C13-H*, 2H), 7.64 (d, *J* = 1.6 Hz, C8-H, 1H), 7.51 (d, *J* = 8.5 Hz, C5-H, 1H), 7.44 (dd, *J* = 8.6, 1.6 Hz, C6-H, 1H), 7.03 (dd, *J* = 6.9, 5.9 Hz, C17-H, 1H), 6.96 (t, *J* = 5.9 Hz, C18-H, 1H), 3.83–3.72 (m, *N-CH_2_* (*morpholine*), 8H), 3.66 (dd, *J* = 17.2, 4.4 Hz, *O-CH_2_* (*morpholine*), 8H).

**^13^C NMR** (101 MHz, DMSO-d_6_) δ 164.77, 158.22, 157.90 154.18, 152.79, 147.38, 143.58, 139.31, 136.78, 132.99, 127.44, 125.98, 122.01, 119.10, 117.58, 112.19, 110.09, 108.51, 66.14, 65.95, 49.84, 44.14.

**MALDI** (DCTB, pos. mode): 512.0 (M+H) 100%, 510.0 (M^+^) 26%, (M+Na) 4.6%.

**Elemental analysis**: for C_28_H_29_N_7_O_3_ calculated: C, 65.74; H, 5.71; found: C, 65.66; H, 5.93.

#### 3.3.9. 1-(4-(2,4-Dimorpholinoquinazolin-7-yl)phenyl)-3-(naphthalen-1-yl)urea **7i**

Amine: naphthalen-1-amine (18,3 mg, 0.128 mmol, 1 equiv.)

Eluent: petroleum ether-ethyl acetate (1:1). Beige solid, yield 97%.

**M.p.** = 228–230 °C.

**^1^H NMR** (400 MHz, DMSO-d_6_) δ 10.77 (s, *NH*, 1H), 9.78 (s, *NH*, 1H), 8.58–8.53 (m, *C22-H*, 1H), 8.09 (dd, J = 7.6, 0.8 Hz, *C15-H*, 1H), 7.93–7.88 (m, *C19-H*, 1H), 7.84 (d, J = 8.7 Hz, *C5-H*, 1H), 7.77–7.68 (m, *C10-H*, *C11-H*, *C13-H*, *C14-H*, 4H), 7.64 (d, J = 1.8 Hz, *C8-H*, 1H), 7.60 (d, J = 8.2 Hz, *C18-H*, 1H), 7.56–7.50 (m, *C20-H*, *C21-H*, 2H), 7.46 (m, *C6-H*, *C17-H*, 2H), 3.82–3.75 (m, J = 10.0, 5.9 Hz, *N-CH_2_* (*morpholine*), 8H), 3.70–3.60 (m, *O-CH_2_* (*morpholine*), 8H).

**^13^C NMR** (101 MHz, DMSO-d_6_) δ 164.79, 157.92, 154.23, 153.26, 143.79, 140.69, 134.76, 133.73, 131.84, 128.18, 127.31, 125.92, 125.79, 125.48, 122.45, 122.28, 121.80, 119.42, 119.25, 118.10, 116.73, 116.39, 109.98, 66.15, 65.95, 49.85, 44.15.

**MALDI** (pos. mode, no matrix): 561.2 (M+H) 100%.

**Elemental analysis**: for C_33_H_32_N_6_O_3_ calculated: C, 70.70; H, 5.75; found: C, 70.76; H, 5.59.

#### 3.3.10. Methyl 4-(3-(4-(2,4-Dimorpholinoquinazolin-7-yl)phenyl)ureido)benzoate **7j**

Amine: methyl 4-aminobenzoate (19.3 mg, 0.128 mmol, 1 equiv.)

Eluent: petroleum ether-ethyl acetate (1:1). Light yellow solid, yield 73%.

**M.p.** = 178–180 °C.

**^1^H NMR** (400 MHz, DMSO-d_6_) δ 9.63 (s, *NH*, 1H), 9.44 (s, *NH*, 1H), 7.90 (d, J = 8.8 Hz, *C16-H*, *C20-H*, 2H), 7.84 (d, J = 8.6 Hz, *C5-H*, 1H), 7.74 (d, J = 8.7 Hz, *C17-H*, *C19-H*, 2H), 7.65–7.61 (m, *C10-H*, *C11-H*, *C13-H*, *C14-H*, 4H), 7.60 (d, J = 1.9 Hz, *C8-H*, 1H), 7.43 (dd, J = 8.6, 1.9 Hz, *C6-H*, 1H), 3.82 (s, *OCH_3_*, 3H), 3.81–3.75 (m, *N-CH_2_* (*morpholine*), 8H), 3.70–3.60 (m, *O-CH_2_* (*morpholine*), 8H).

**^13^C NMR** (101 MHz, DMSO-d_6_) δ 165.93, 164.78, 157.92, 154.22, 152.23, 144.43, 143.64, 139.74, 132.59, 130.41, 127.39, 125.95, 122.39, 121.98, 119.42, 118.56, 117.22, 110.07, 66.14, 65.95, 49.85, 44.15, 28.98.

**MALDI** (DCTB, pos. mode): 569.2 (M+H) 100%.

**Elemental analysis**: for C_31_H_32_N_6_O_5_ calculated: C, 65.48; H, 5.67; found: C, 65.62; H, 5.75.

#### 3.3.11. Ethyl 4-((4-(2,4-Dimorpholinoquinazolin-7-yl)phenyl)carbamoyl)piperazine-1-carboxylate **7k**

Amine: ethyl piperazine-1-carboxylate (23.4 mg, 0.128 mmol, 1 equiv.)

Eluent: petroleum ether-ethyl acetate (1:1). Yellow solid, yield 95%.

**M.p**. = 109–111 °C.

**^1^H NMR** (400 MHz, DMSO-d_6_) δ 8.79 (s, *NH*, 1H), 7.83 (d, J = 8.7 Hz, *C5-H*, 1H), 7.68 (d, J = 8.9 Hz, *C10-H*, *C14-H*, 2H), 7.61 (d, J = 8.9 Hz, *C11-H*, *C13-H*, 2H), 7.60 (d, J = 1.9 Hz, *C8-H*, 1H), 7.42 (dd, J = 8.7, 1.9 Hz, *C6-H*, 1H), 4.08 (q, *J* = 7.1 Hz, *C19-H*, 2H), 3.82–3.75 (m, *N-CH_2_* (*morpholine*), 8H), 3.70–3.60 (m, *O-CH_2_* (*morpholine*), 8H), 3.51–3.46 (m, *C15-H*, *C18-H*, 4H), 3.43–3.39 (m, *C16-H*, *C17-H* 4H), 1.20 (t, J = 7.1 Hz, *C20-H*, 3H).

**^13^C NMR** (101 MHz, DMSO-d_6_) δ 164.77, 157.90, 154.80, 154.65, 154.55, 143.77, 140.82, 132.21, 126.89, 125.90, 121.90, 119.73, 119.41, 110.00, 66.14, 65.95, 60.88, 50.69, 49.84, 44.14, 43.55, 14.58.

**MALDI** (DCTB, pos. mode): 576.4 (M+H) 100%.

**Elemental analysis**: for C_30_H_37_N_7_O_5_ calculated: C, 62.59; H, 6.48; found: C, 62.27; H, 6.24.

#### 3.3.12. 1-(4-(2,4-Dimorpholinoquinazolin-7-yl)phenyl)-3-(prop-2-yn-1-yl)urea **7l**

Amine: prop-2-yn-1-amine (7 mg, 0.128 mmol, 1 equiv.)

Eluent: petroleum ether-ethyl acetate (1:2). Beige solid, yield 36%.

**M.p.** = 168–170 °C.

**^1^H NMR** (400 MHz, DMSO-d_6_) δ 9.02 (d, *J* = 7.4 Hz, NH, 1H), 8.97 (d, *J* = 7.3 Hz, NH, 1H), 8.10 (d, *J* = 8.8 Hz, C5-H, 1H), 7.97 (d, *J* = 0.5 Hz, C8-H, 1H), 7.74 (d, *J* = 8.4 Hz, C10-H, C14-H, 2H), 7.62 (d, *J* = 8.5 Hz, C11-H, C13-H, 2H), 7.45 (dd, *J* = 8.5, 0.6 Hz, C6-H, 1H), 3.86 (d, *J* = 4.3 Hz, C15-H, 2H), 3.80–3.77 (m, C17-H, *N-CH_2_* (*morpholine*), 9H), 3.71–3.65 (m, *O-CH_2_* (*morpholine*), 8H).

**^13^C NMR** (101 MHz, DMSO-d_6_) δ 164.21, 155.70, 153.48, 152.32, 144.59, 140.50, 131.33, 127.71, 126.49, 124.00, 122.96, 118.60, 109.96, 79.16, 66.79, 66.11, 65.94, 49.37, 44.19, 33.27.

**MALDI** (no matrix. pos. mode): 392.0 (100%), 418.0 (74%), 427.1 (23%), 425.1 (17%), 470.1 (M^+^) 6%.

**Elemental analysis**: for C_26_H_28_N_6_O_3_ calculated: C, 66.09; H, 5.97; found: C, 66.17; H, 6.05.

### 3.4. Biological Assays

#### 3.4.1. Cytotoxicity of the Target Compounds

Human breast cancer MCF7, MDA-MB-231, SkBr-3 (purchased from the ATCC collection), human keratinocytes HaCaT, murine fibroblasts L929 and macrophages J774 cells (Collection of Shemyakin-Ovchinnikov Institute of Bioorganic Chemistry RAS) were cultured in standard DMEM or RPMI-1640 medium (ThermoScientific, Waltham, MA, USA) supplemented with 10% fetal bovine serum (FBS, Cytiva, Marlborough, MA, USA) at 37 °C, 5 % CO_2_ and 80–85% humidity (NuAir CO_2_ incubator). The cell growth was evaluated by MTT (3-[4,5-dimethylthiazol-2-yl]-2,5-diphenyltetrazolium bromide) test [75]. Cells were seeded at a density of 5 × 10^3^ cells per well in 96-well flat-bottomed plates (TPP). The compounds were dissolved in DMSO (AppliChem) to 10 mM before the experiments, and then the resulting solutions were diluted by the medium to the required concentrations. Solutions of the tested compounds with different concentrations in 100 μL of the medium were added and the cells were grown for 72 h. MTT (10 μL/well, 5 mg/mL) was added for the final 3 h. The formazan purple crystals were dissolved in DMSO (100 μL per well). The absorbance of solutions was measured at 540 nm with a MultiScan reader (ThermoFisher). Results were analyzed by Excel package (Microsoft). The inhibition of proliferation (inhibitory index) was calculated as [1–(ODexperiment/ODcontrol)], where OD was MTT optical density. IC_50_ (Table 1) was determined as the concentration giving 50% of inhibition in the comparison with it at the highest concentration of the compounds.

#### 3.4.2. Immunoblotting

MCF7 cells were seeded onto 100 mm dishes (Corning) and cultivated for 24 h. Compound **7c** was added at different concentrations while the reference compound wortmannin (Selleckchem, Houston, TX, USA) was used at 500 nM. The cells were harvested after 72 h of growth with the compounds. To prepare cell extracts, MCF7 cells were twice washed in phosphate buffer and incubated for 10 min on ice in the modified lysis buffer containing 50 mM Tris-HCl, pH 7.5, 0.5% Igepal CA-630, 150 mM NaCl, 1 mM EDTA, 1 mM DTT, 1 mM PMSF, 0.1 mM sodium orthovanadate and aprotinin, leupeptin, pepstatin (1 µg/mL each). The protein content was determined by the Bradford method. Cell lysates were separated in 10% SDS-PAGE under reducing conditions, transferred to a nitrocellulose membrane (GE HealthCare, Chicago, IL, USA), and processed according to a standard protocol. To prevent nonspecific absorption, the membranes were treated with 5% nonfat milk solution in TBS buffer (20 mM Tris, 500 mM NaCl, pH 7.5) with 0.1% Tween-20 and then incubated with primary antibodies (Cell Signaling Technology, Danvers, MA, USA) overnight at 4 °C. Goat anti-rabbit IgG (Jackson ImmunoResearch, West Grove, PA, USA), conjugated to horseradish peroxidase, were used as the secondary antibodies. Signals were detected using the ECL reagent [76] using the ImageQuant LAS4000 system (GE HealthCare, Chicago, IL, USA).

The signals of phospho-proteins to total ones were calculated using the ImageJ program from inverted images of the bands.

#### 3.4.3. PI3K Inhibition Assay

The PI 3-Kinase (Class I) HTRF Assay Kit (Cat# 33-016 from Millipore) was used to determine the activity of recombinant PI3K (p110α/p85α) human (Cat# 14-602 from Millipore). The kit was supplemented with ATP (Cat# A26209 from Sigma-Aldrich, St. Louis, MI, USA), DL-dithiothreitol (Cat# S-D5545-1 from Sigma-Aldrich), and Wortmannin inhibitor (Cat# 12-338 from Millipore, Burlington, MA, USA). The latter was used at 0.1 μM concentration.

For measurements, a Victor ×5 plate reader (PerkinElmer, 2030, Waltham, MA, USA) was used in TRF mode with excitation at 340 nm. Fluorescence emission was recorded at 613 and 670 nm in a 384-well plate (Cat# S-CLS3658 from Sigma-Aldrich) at a time interval of 150–350 μs. The enzymatic reaction and the determination of its products were carried out according to the recommendations of the reagent kit manufacturer (Millipore) in two wells for each sample. In calculations, readings in parallel wells were averaged. The calculations were made according to the recommendations of the equipment manufacturer.

#### 3.4.4. Cell Cycle Analysis

The cell cycle was analyzed using PI-stained DNA. Cells from the compounds **7** treated cultures were collected at 24, 48, and 72 h, trypsinized, washed in ice-cold PBS, fixed by the addition of 70% ethanol and left for 2 h at −20 °C. Thereafter, the cells were washed twice in PBS, stained with 50 μg/mL of propidium iodide (Sigma, Merck KGaA, Darmstadt, Germany) in PBS, treated with 10 µg/mL of RNAse and analyzed by flow cytometry using FACScan device (BD, USA). A total of 2000 events were collected. The results were analyzed using FlowJo 10 software (BD, Franklin Lakes, NJ, USA).

*Apoptosis analysis*

Apoptosis was analyzed by flow cytometry using a FACScan device (BD, USA). Cells were incubated with the compounds **7** as above, trypsinized, washed in ice-cold PBS, stained with annexin V-FITC (BD, USA, Franklin Lakes, NJ) and propidium iodide (Sigma, Merck KGaA, Darmstadt, Germany), incubated for 15 min, and analyzed.

#### 3.4.5. Reactive Oxygen Species (ROS) Production

MCF7 and SkBr-3 cells were seeded in 96-well flat-bottom plates at 3000 cells/well and incubated in a CO_2_ incubator at 37 °C overnight to reach an adhesive state. The compounds **7** at different concentrations were added for 2 h. ROS probe 2’,7’-dichlorodihydrofluorescein diacetate (DCF, Sigma, USA) was added simultaneously with the compounds. After cell incubation for 2 h, the supernatants were removed, the cells were washed with PBS, 100 μL of fresh PBS was added, and the fluorescence was analyzed by spectroluminometer GlomaxMulti (Promega, Fitchburg, MA, USA) at 488 nm. The results are shown as the ratios of OD minus background without DCF in the treated cells to the control ones.

#### 3.4.6. Confocal Analysis

For confocal analysis, cells were grown overnight on sterile cover slips inserted into 6-well plates (Costar, Washington, WA, USA) in 200 µL of complete culture medium. Compounds **7** were added in 10 µL and incubated with the cells overnight. LysoTrackRed, WGA-AlexaFluor555 (Applied Biosystems, Foster City, CA, USA), and Hoechst 33,342 (Sigma, Merck KGaA, Darmstadt, Germany) were added for 30 min. Cells were fixed with 1% paraformaldehyde, washed, and polymerized with Mowiol 4.88 medium (Calbiochem, Germany). Slides were analyzed using an Eclipse TE2000 confocal microscope (Nikon, Japan). ROS production was analyzed in the same way 2 h after the addition of a compound of type **7**.

#### 3.4.7. In Vivo Studies

Murine colorectal CT26 (ATCC, CRL-2639) adenocarcinoma tumor cell line was used in the study. Cells were grown in RPMI-1640 supplemented with 10% fetal calf serum (FCS) and pen-strep-glut (all from PanEco, Moscow, Russian Federation). Cells were passaged by trypsinization using Trypsin/EDTA solution (PanEco, Moscow, Russian Federation) twice a week. Cell authentication was not performed by the authors.

Six- to eight-week-old female BALB/c mice were obtained from the Pushchino branch of Shemyakin-Ovchinnikov Institute of Bioorganic Chemistry RAS. All studies were conducted in an AAALAC accredited facility in compliance with the PHS Guidelines for the Care and Use of Animals in Research, protocol #325 from 24.05.2021

Colon-26 cells (10^5^) were subcutaneously (s.c.) injected into the depilated flanks of 6-week-old female BALB/c mice. Mice (*n* = 20) were divided in three groups: control mice (*n* = 8) were injected with vehicle only; mice from group 1 (*n* = 6) and 2 (*n* = 6) were treated with 50 µL of 1 mM **7c** injected s.c. near the tumors. Treatment of the group 1 mice was started as soon as the tumors were visible (~2–8 mm^3^ tumor volumes) once in 3 days; tumors in group 2 were left to grow till 400–500 mm^3^ and then treated with 50 µL of 1 mM **7c** injected s.c. around the tumors. The vehicle contained 2.5 µL DMSO in PBS. The perpendicular diameter of each tumor was measured twice a week, and tumor volume was calculated using the following formula: tumor volume (mm^3^) = *a* × *b*^2^ × 0.5, where *a* represents the longest diameter, *b* represents the shortest diameter, and 0.5 is a constant used to calculate the volume of an ellipsoid. Mice were sacrificed by cervical dislocation.

#### 3.4.8. Docking Studies

Preliminary optimization of the geometry of ligands was carried out using the Gaussian 03 program [Gaussian] by the density functional method using the B3LYP functional in the 6–31G(d, p) basis. Further, with the final optimized structures, molecular docking was performed (calculation of the most favorable conformation of the complex and binding energy, as well as contacts with amino acid residues of the protein) at the site of phosphorylation of PI3Kα and PI3Kγ protein isoforms. The calculation was performed using the Autodock 4.2 program [77], exploiting the AutoGrid program to create a docking area. The shell MGLTools 1.5.6 was used to prepare the initial data. Each docking experiment was performed using the LGA genetic algorithm (100 conformations, 2,500,000 calculations each). The crystal structures of the p110a subunit of the PI3Kα (4TV3.pdb [73]) and PI3Kγ (7JWE.pdb [74]) proteins were applied. Clustering of ligand conformations in the active site of the receptor (association of conformers with similar atomic coordinates into groups-clusters) of docking conformations of the ligand in the active site of the kinase was carried out according to the maximum distance between cluster individuals (RMSD) 2Å. The cluster with the most negative binding Gibbs energy was taken as the most correct solution. Free energy calculations were carried out in a semi-empirical force field [78]. Visualization of docking results (protein–ligand complexes) was carried out using the PLIP service. The superposition of ligands was visualized using the UCSF Chimera program [79].

## 4. Conclusions

A library of new quinazoline-based inhibitors of PI3K/Akt/mTOR was synthesized. The compounds exhibit cytotoxicity in the low micromolar range. The inhibitor **7c**, bearing a pyridine moiety and a flexible spacer in the variable part of the molecule, demonstrated a significant increase in antiproliferative activity (up to 4 nM) while being used in the combination with metformin, indicating a significant synergism for these compounds. According to the MTT assay, compound **7c** possesses the highest activity. Importantly, **7c** demonstrated the selectivity towards MCF7 cells, that expose the mutant type of PI3K in contrast to MDA-MB-231 cells. It was selected for further biological assays, targeting the PI3K/Akt/mTOR pathway. Compound **7c** moderately induces apoptosis, and causes ROS production, lysosome membranes depolarization, and autophagy in low micromolar concentrations. Triggering the PARP-1 cleavage already at 62–125 nM **7c** induces cell death via several mechanisms simultaneously. This fact requires more detailed investigation in the future. Inhibition of key effectors of the PI3K/Akt/mTOR cascade after treatment with **7c** was observed already at 125–250 nM. Lead compounds **7b** and **7c** demonstrated the inhibition of PI3Kα; however, IC_50_ was relatively high—about 50 µM and 500 µM for **7b** and **7c,** respectively. Such moderate inhibition potency taken collectively with the immunoblotting data may suggest the PI3K as a secondary target among kinases’ PI3K/Akt/mTOR cascade. In vivo antitumor efficiency of **7c** was shown by exploiting the xenograft mouse model. In the case of early treatment, a significant effect was observed after three injections. Late treatment of large tumors with compound **7c** results in tumor destruction. Docking studies of **7c** and several other compounds allow the identification of the important structural fragments, responsible for the interactions with the amino acids in the catalytic site of the target kinases. Based on the docking results, we can anticipate the potential selectivity of the synthesized compounds towards PI3Kα in contrast to the PI3Kγ isoform.

In summary, synthesized quinazoline-based compounds demonstrated in vitro and in vivo unusual multitarget activity. Despite the fact that the leader compounds alone with a strong influence on the PI3K/Akt/mTOR signaling pathway also damage DNA and stimulate autophagy, depolarize lysosomes, and cleave PARP, based on the available data, we cannot choose the main target of their action. Clarification of this issue, studying the profile of enzymatic activity, and optimizing the structures of compounds will allow in the future to design promising agents for the treatment of various forms of cancer.

## Figures and Tables

**Table 1:** Antiproliferative activity of compounds **7a**–**l**, IC_50_^a^, μM.

Compounds	Cell Lines				
	MCF7	MDA-MB-231	J774	HaCaT	L929
**7a**	6.6	6.7	Nd ^c^	nd	nd
**7b**	**1.1 ^b^**	**1.7**	**1.6**	**0.8**	12
**7c**	**0.2**	**1**	**0.4**	**6**	**3**
**7d**	5.2	10.5	nd	nd	nd
**7e**	9.5	24	12	12	12
**7f**	6.5	8.8	6	3	12
**7g**	4.9	14	6	25	25
**7h**	13	14	6	3	12
**7i**	6.1	11	6	6	6
**7j**	**3.8**	**4.1**	**0.8**	**3**	**3**
**7k**	11	17	3	6	25
**7l**	>50	>50	6	3	>50

^a^ Standard deviations were within 50% and not shown for the easier reading of the results. ^b^ Effective compounds are shown in red. ^c^ nd: not determined.

**Table 2:** Docking results of the selected compounds and gedatolisib into catalytic sites of PI3Kα and PI3Kγ.

Protein	Ligand		RMSD, A	Binding Energy, kcal/mol	K_inh_ nM	Number of H-Bonds	Contacts with Amino Acids
PI3Kα (4TV3)	Gedatolisib		32.340	−10.93	9.8	3	Ala775, Thr856, Ile932, **Ser774**, **Val851**, **Ser854**, His917, Asp805, Asp915, Asp933
	**6**		35.272	−9.38	133	3	Thr856, Ile932, **Val851**, **Asn853**, **Ser919**
	**7a**		36.007	−10.72	13.9	4	Trp780, **Lys802**, **Val851, Asn853**, **Ser854**
	**7b**		33.765	−11.65	**2.88**	6	**Lys802**, Ile932, Trp780, Val850, Thr856, **Val851**, **Asn853**, **Ser854**, **His855**, Gln859
	**7c**		34.876	−11.18	**6.37**	6	Trp780, Ile800, **Val850**, Thr856, **Lys802**, Val851, **Asn853**, **Ser854**, Ile932, **His855**, **Gln859**
PI3Kγ (7JWE)	Gedatolisib		30.451	−12.79	**0.42**	7	Asp836, Lys875, Ile963, **Asp964**, **Asp841**, **Val882**, **Phe965**, Gly966, His967, **Asp837**
		**6**	32.494	−9.98	48.1	6	Lys833, Leu838, Tyr867, Ile879, Ile963, **Asp964**, Ser806, **Asp836**, **Asp837**, **Asp841**, **His967**
		**7a**	28.646	−10.28	29.1	6	**Lys833**, Leu838, Tyr867, Ile879, Ile963, Asp964, **Ser806**, **Asp836**, **Asp837**, **Asp841**, **His967**
		**7b**	30.748	−10.26	30.3	6	**His948**, His967, Leu1090, **Val882**, **Asp964**, **Ser806**, **Lys807**, **Lys808**
		**7c**	30.800	−10.62	16.4	5	**His948**, Ile963, His967, Leu1090, **Val882**, **Arg947**, **Asp964**, Trp1086

## Data Availability

Not applicable.

## Supplementary Materials

The supporting information can be downloaded at: https://www.mdpi.com/article/10.3390/ijms231810854/s1.

## References

[1] Fruman D.A., Chiu H., Hopkins B.D., Bagrodia S., Cantley L.C., Abraham R.T. (2017). The PI3K Pathway in Human Disease. Cell.

[2] Yang J., Nie J., Ma X., Wei Y., Peng Y., Wei X. (2019). Targeting PI3K in Cancer: Mechanisms and Advances in Clinical Trials. Mol. Cancer.

[3] Fruman D.A., Rommel C. (2014). PI3K and Cancer: Lessons, Challenges and Opportunities. Nat. Rev. Drug Discov..

[4] Jiang N., Dai Q., Su X., Fu J., Feng X., Peng J. (2020). Role of PI3K/AKT Pathway in Cancer: The Framework of Malignant Behavior. Mol. Biol. Rep..

[5] Sampaio N.G., Yu W., Cox D., Wyckoff J., Condeelis J., Stanley E.R., Pixley F.J. (2011). Phosphorylation of CSF-1R Y721 Mediates Its Association with PI3K to Regulate Macrophage Motility and Enhancement of Tumor Cell Invasion. J. Cell Sci..

[6] Schmid M.C., Avraamides C.J., Dippold H.C., Franco I., Foubert P., Ellies L.G., Acevedo L.M., Manglicmot J.R.E., Song X., Wrasidlo W. (2011). Receptor Tyrosine Kinases and TLR/IL1Rs Unexpectedly Activate Myeloid Cell PI3Kγ, A Single Convergent Point Promoting Tumor Inflammation and Progression. Cancer Cell.

[7] Hirsch E., Ciraolo E., Franco I., Ghigo A., Martini M. (2014). PI3K in Cancer–Stroma Interactions: Bad in Seed and Ugly in Soil. Oncogene.

[8] Dituri F., Mazzocca A., Giannelli G., Antonaci S. (2011). PI3K Functions in Cancer Progression, Anticancer Immunity and Immune Evasion by Tumors. Clin. Dev. Immunol..

[9] She Q.-B., Halilovic E., Ye Q., Zhen W., Shirasawa S., Sasazuki T., Solit D.B., Rosen N. (2010). 4E-BP1 Is a Key Effector of the Oncogenic Activation of the AKT and ERK Signaling Pathways That Integrates Their Function in Tumors. Cancer Cell.

[10] Lee T., Yao G., Nevins J., You L. (2008). Sensing and Integration of Erk and PI3K Signals by Myc. PLoS Comput. Biol..

[11] Dey N., Leyland-Jones B., De P. (2015). MYC-Xing It up with PIK3CA Mutation and Resistance to PI3K Inhibitors: Summit of Two Giants in Breast Cancers. Am. J. Cancer Res..

[12] Thorpe L.M., Yuzugullu H., Zhao J.J. (2015). PI3K in Cancer: Divergent Roles of Isoforms, Modes of Activation and Therapeutic Targeting. Nat. Rev. Cancer.

[13] Janku F., Yap T.A., Meric-Bernstam F. (2018). Targeting the PI3K Pathway in Cancer: Are We Making Headway?. Nat. Rev. Clin. Oncol..

[14] Gulluni F., De Santis M.C., Margaria J.P., Martini M., Hirsch E. (2019). Class II PI3K Functions in Cell Biology and Disease. Trends Cell Biol..

[15] Jean S., Kiger A.A. (2014). Classes of Phosphoinositide 3-Kinases at a Glance. J. Cell Sci..

[16] Nobukuni T., Joaquin M., Roccio M., Dann S.G., Kim S.Y., Gulati P., Byfield M.P., Backer J.M., Natt F., Bos J.L. (2005). Amino Acids Mediate MTOR/Raptor Signaling through Activation of Class 3 Phosphatidylinositol 3OH-Kinase. Proc. Natl. Acad. Sci. USA.

[17] Martini M., De Santis M.C., Braccini L., Gulluni F., Hirsch E. (2014). PI3K/AKT Signaling Pathway and Cancer: An Updated Review. Ann. Med..

[18] Franke T.F. (2008). PI3K/Akt: Getting It Right Matters. Oncogene.

[19] Revathidevi S., Munirajan A.K. (2019). Akt in Cancer: Mediator and More. Semin. Cancer Biol..

[20] Cerami E., Gao J., Dogrusoz U., Gross B.E., Sumer S.O., Aksoy B.A., Jacobsen A., Byrne C.J., Heuer M.L., Larsson E. (2012). The CBio Cancer Genomics Portal: An Open Platform for Exploring Multidimensional Cancer Genomics Data: Figure 1. Cancer Discov..

[21] Stemke-Hale K., Gonzalez-Angulo A.M., Lluch A., Neve R.M., Kuo W.-L., Davies M., Carey M., Hu Z., Guan Y., Sahin A. (2008). An Integrative Genomic and Proteomic Analysis of PIK3CA, PTEN, and AKT Mutations in Breast Cancer. Cancer Res..

[22] Yang H., Rudge D.G., Koos J.D., Vaidialingam B., Yang H.J., Pavletich N.P. (2013). MTOR Kinase Structure, Mechanism and Regulation. Nature.

[23] Zou Z., Tao T., Li H., Zhu X. (2020). MTOR Signaling Pathway and MTOR Inhibitors in Cancer: Progress and Challenges. Cell Biosci..

[24] Heavey S., O’Byrne K.J., Gately K. (2014). Strategies for Co-Targeting the PI3K/AKT/MTOR Pathway in NSCLC. Cancer Treat. Rev..

[25] Matasar M.J., Capra M., Özcan M., Lv F., Li W., Yañez E., Sapunarova K., Lin T., Jin J., Jurczak W. (2021). Copanlisib plus Rituximab versus Placebo plus Rituximab in Patients with Relapsed Indolent Non-Hodgkin Lymphoma (CHRONOS-3): A Double-Blind, Randomised, Placebo-Controlled, Phase 3 Trial. Lancet Oncol..

[26] Castel P., Toska E., Engelman J.A., Scaltriti M. (2021). The Present and Future of PI3K Inhibitors for Cancer Therapy. Nat. Cancer.

[27] Baselga J., Im S.-A., Iwata H., Cortés J., De Laurentiis M., Jiang Z., Arteaga C.L., Jonat W., Clemons M., Ito Y. (2017). Buparlisib plus Fulvestrant versus Placebo plus Fulvestrant in Postmenopausal, Hormone Receptor-Positive, HER2-Negative, Advanced Breast Cancer (BELLE-2): A Randomised, Double-Blind, Placebo-Controlled, Phase 3 Trial. Lancet Oncol..

[28] Garces A.E., Stocks M.J. (2019). Class 1 PI3K Clinical Candidates and Recent Inhibitor Design Strategies: A Medicinal Chemistry Perspective. J. Med. Chem..

[29] Sabbah D.A., Hajjo R., Bardaweel S.K., Zhong H.A. (2021). Phosphatidylinositol 3-Kinase (PI3K) Inhibitors: A Recent Update on Inhibitor Design and Clinical Trials (2016–2020). Expert Opin. Ther. Pat..

[30] Hillmann P., Fabbro D. (2019). PI3K/MTOR Pathway Inhibition: Opportunities in Oncology and Rare Genetic Diseases. Int. J. Mol. Sci..

[31] Peng Y., Wang Y., Zhou C., Mei W., Zeng C. (2022). PI3K/Akt/MTOR Pathway and Its Role in Cancer Therapeutics: Are We Making Headway?. Front. Oncol..

[32] Borsari C., Rageot D., Beaufils F., Bohnacker T., Keles E., Buslov I., Melone A., Sele A.M., Hebeisen P., Fabbro D. (2019). Preclinical Development of PQR514, a Highly Potent PI3K Inhibitor Bearing a Difluoromethyl–Pyrimidine Moiety. ACS Med. Chem. Lett..

[33] Hu J., Zhang Y., Tang N., Lu Y., Guo P., Huang Z. (2021). Discovery of Novel 1,3,5-Triazine Derivatives as Potent Inhibitor of Cervical Cancer via Dual Inhibition of PI3K/MTOR. Bioorg. Med. Chem..

[34] Mahajan D., Sen S., Kuila B., Sharma A., Arora R., Sagar M., Mahapatra A.R., Gawade L.B., Dugar S. (2020). Discovery and Development of SPR519 as a Potent, Selective, and Orally Bioavailable Inhibitor of PI3Kα and MTOR Kinases for the Treatment of Solid Tumors. J. Med. Chem..

[35] Wu T.-T., Guo Q.-Q., Chen Z.-L., Wang L.-L., Du Y., Chen R., Mao Y.-H., Yang S.-G., Huang J., Wang J.-T. (2020). Design, Synthesis and Bioevaluation of Novel Substituted Triazines as Potential Dual PI3K/MTOR Inhibitors. Eur. J. Med. Chem..

[36] Rageot D., Bohnacker T., Keles E., McPhail J.A., Hoffmann R.M., Melone A., Borsari C., Sriramaratnam R., Sele A.M., Beaufils F. (2019). (S)-4-(Difluoromethyl)-5-(4-(3-Methylmorpholino)-6-Morpholino-1,3,5-Triazin-2-Yl)Pyridin-2-Amine (PQR530), a Potent, Orally Bioavailable, and Brain-Penetrable Dual Inhibitor of Class I PI3K and MTOR Kinase. J. Med. Chem..

[37] Yaguchi S., Izumisawa Y., Sato M., Nakagane T., Koshumizu I., Sakita K., Kato M., Yoshioka K., Sakata M., Kawashima S. (1997). In Vitro Cytotoxicity of Imidazolyl-1,3,5-Triazine Derivatives. Biol. Pharm. Bull..

[38] Xiang H.-Y., Wang X., Chen Y.-H., Zhang X., Tan C., Wang Y., Su Y., Gao Z.-W., Chen X.-Y., Xiong B. (2021). Identification of Methyl (5-(6-((4-(Methylsulfonyl)Piperazin-1-Yl)Methyl)-4-Morpholinopyrrolo[2,1-f][1,2,4]Triazin-2-Yl)-4-(Trifluoromethyl)Pyridin-2-Yl)Carbamate (CYH33) as an Orally Bioavailable, Highly Potent, PI3K Alpha Inhibitor for the Treatment of Advanced Solid Tumors. Eur. J. Med. Chem..

[39] Yang C., Lu M., Chen Y., Xiang R., Qiu T., Jia Y., Yang Y., Liu X., Deng M., Ling Y. (2021). Development of Anti-Breast Cancer PI3K Inhibitors Based on 7-Azaindole Derivatives through Scaffold Hopping: Design, Synthesis and In Vitro Biological Evaluation. Bioorg. Chem..

[40] Gangadhara G., Dahl G., Bohnacker T., Rae R., Gunnarsson J., Blaho S., Öster L., Lindmark H., Karabelas K., Pemberton N. (2019). A Class of Highly Selective Inhibitors Bind to an Active State of PI3Kγ. Nat. Chem. Biol..

[41] Pemberton N., Mogemark M., Arlbrandt S., Bold P., Cox R.J., Gardelli C., Holden N.S., Karabelas K., Karlsson J., Lever S. (2018). Discovery of Highly Isoform Selective Orally Bioavailable Phosphoinositide 3-Kinase (PI3K)-γ Inhibitors. J. Med. Chem..

[42] Sun Y., Fu R., Lin S., Zhang J., Ji M., Zhang Y., Wu D., Zhang K., Tian H., Zhang M. (2021). Discovery of New Thieno[2,3-d]Pyrimidine and Thiazolo[5,4-d]Pyrimidine Derivatives as Orally Active Phosphoinositide 3-Kinase Inhibitors. Bioorg. Med. Chem..

[43] Xia L., Zhang Y., Zhang J., Lin S., Zhang K., Tian H., Dong Y., Xu H. (2020). Identification of Novel Thiazolo[5,4-b]Pyridine Derivatives as Potent Phosphoinositide 3-Kinase Inhibitors. Molecules.

[44] Xiao Y., Yu Y., Jiang P., Li Y., Wang C., Zhang R. (2020). The PI3K/MTOR Dual Inhibitor GSK458 Potently Impedes Ovarian Cancer Tumorigenesis and Metastasis. Cell. Oncol..

[45] Thakur A., Tawa G.J., Henderson M.J., Danchik C., Liu S., Shah P., Wang A.Q., Dunn G., Kabir M., Padilha E.C. (2020). Design, Synthesis, and Biological Evaluation of Quinazolin-4-One-Based Hydroxamic Acids as Dual PI3K/HDAC Inhibitors. J. Med. Chem..

[46] Das D., Hong J. (2019). Recent Advancements of 4-Aminoquinazoline Derivatives as Kinase Inhibitors and Their Applications in Medicinal Chemistry. Eur. J. Med. Chem..

[47] Teng Y., Li X., Ren S., Cheng Y., Xi K., Shen H., Ma W., Luo G., Xiang H. (2020). Discovery of Novel Quinazoline Derivatives as Potent PI3Kδ Inhibitors with High Selectivity. Eur. J. Med. Chem..

[48] Zhang K., Lai F., Lin S., Ji M., Zhang J., Zhang Y., Jin J., Fu R., Wu D., Tian H. (2019). Design, Synthesis, and Biological Evaluation of 4-Methyl Quinazoline Derivatives as Anticancer Agents Simultaneously Targeting Phosphoinositide 3-Kinases and Histone Deacetylases. J. Med. Chem..

[49] Zhang K., Ji M., Lin S., Peng S., Zhang Z., Zhang M., Zhang J., Zhang Y., Wu D., Tian H. (2021). Design, Synthesis, and Biological Evaluation of a Novel Photocaged PI3K Inhibitor toward Precise Cancer Treatment. J. Med. Chem..

[50] Miller M., Thompson P., Gabelli S. (2019). Structural Determinants of Isoform Selectivity in PI3K Inhibitors. Biomolecules.

[51] Venkatesan A.M., Dehnhardt C.M., Delos Santos E., Chen Z., Dos Santos O., Ayral-Kaloustian S., Khafizova G., Brooijmans N., Mallon R., Hollander I. (2010). Bis(Morpholino-1,3,5-Triazine) Derivatives: Potent Adenosine 5′-Triphosphate Competitive Phosphatidylinositol-3-Kinase/Mammalian Target of Rapamycin Inhibitors: Discovery of Compound 26 (PKI-587), a Highly Efficacious Dual Inhibitor. J. Med. Chem..

[52] Nunnery S.E., Mayer I.A. (2019). Management of Toxicity to Isoform α-Specific PI3K Inhibitors. Ann. Oncol..

[53] Hopkins B.D., Pauli C., Du X., Wang D.G., Li X., Wu D., Amadiume S.C., Goncalves M.D., Hodakoski C., Lundquist M.R. (2018). Suppression of Insulin Feedback Enhances the Efficacy of PI3K Inhibitors. Nature.

[54] Sorokin D., Shchegolev Y., Scherbakov A., Ryabaya O., Gudkova M., Berstein L., Krasil’nikov M. (2020). Metformin Restores the Drug Sensitivity of MCF-7 Cells Resistant Derivates via the Cooperative Modulation of Growth and Apoptotic-Related Pathways. Pharmaceuticals.

[55] Henning R.J., Bourgeois M., Harbison R.D. (2018). Poly(ADP-Ribose) Polymerase (PARP) and PARP Inhibitors: Mechanisms of Action and Role in Cardiovascular Disorders. Cardiovasc. Toxicol..

[56] Chen G., Ding X.-F., Bouamar H., Pressley K., Sun L.-Z. (2019). Everolimus Induces G 1 Cell Cycle Arrest through Autophagy-Mediated Protein Degradation of Cyclin D1 in Breast Cancer Cells. Am. J. Physiol. Physiol..

[57] Yang J., Pi C., Wang G. (2018). Inhibition of PI3K/Akt/MTOR Pathway by Apigenin Induces Apoptosis and Autophagy in Hepatocellular Carcinoma Cells. Biomed. Pharmacother..

[58] Xue J.-F., Shi Z.-M., Zou J., Li X.-L. (2017). Inhibition of PI3K/AKT/MTOR Signaling Pathway Promotes Autophagy of Articular Chondrocytes and Attenuates Inflammatory Response in Rats with Osteoarthritis. Biomed. Pharmacother..

[59] Kim Y.C., Guan K.-L. (2015). MTOR: A Pharmacologic Target for Autophagy Regulation. J. Clin. Investig..

[60] Rong L., Li Z., Leng X., Li H., Ma Y., Chen Y., Song F. (2020). Salidroside Induces Apoptosis and Protective Autophagy in Human Gastric Cancer AGS Cells through the PI3K/Akt/MTOR Pathway. Biomed. Pharmacother..

[61] Lin X., Han L., Weng J., Wang K., Chen T. (2018). Rapamycin Inhibits Proliferation and Induces Autophagy in Human Neuroblastoma Cells. Biosci. Rep..

[62] Stjepanovic G., Davies C.W., Stanley R.E., Ragusa M.J., Kim D.J., Hurley J.H. (2014). Assembly and Dynamics of the Autophagy-Initiating Atg1 Complex. Proc. Natl. Acad. Sci. USA.

[63] Matamala E., Castillo C., Vivar J.P., Rojas P.A., Brauchi S.E. (2021). Imaging the Electrical Activity of Organelles in Living Cells. Commun. Biol..

[64] Cang C., Bekele B., Ren D. (2014). The Voltage-Gated Sodium Channel TPC1 Confers Endolysosomal Excitability. Nat. Chem. Biol..

[65] Cang C., Zhou Y., Navarro B., Seo Y., Aranda K., Shi L., Battaglia-Hsu S., Nissim I., Clapham D.E., Ren D. (2013). MTOR Regulates Lysosomal ATP-Sensitive Two-Pore Na+ Channels to Adapt to Metabolic State. Cell.

[66] Hao J., Zhang W., Huang Z. (2022). Bupivacaine Modulates the Apoptosis and Ferroptosis in Bladder Cancer via Phosphatidylinositol 3-Kinase (PI3K)/AKT Pathway. Bioengineered.

[67] Fan F., Liu P., Bao R., Chen J., Zhou M., Mo Z., Ma Y., Liu H., Zhou Y., Cai X. (2021). A Dual PI3K/HDAC Inhibitor Induces Immunogenic Ferroptosis to Potentiate Cancer Immune Checkpoint Therapy. Cancer Res..

[68] Melero I., Castanon E., Alvarez M., Champiat S., Marabelle A. (2021). Intratumoural Administration and Tumour Tissue Targeting of Cancer Immunotherapies. Nat. Rev. Clin. Oncol..

[69] Walker E.H., Perisic O., Ried C., Stephens L., Williams R.L. (1999). Structural Insights into Phosphoinositide 3-Kinase Catalysis and Signalling. Nature.

[70] Zheng Z., Amran S.I., Thompson P.E., Jennings I.G. (2011). Isoform-Selective Inhibition of Phosphoinositide 3-Kinase: Identification of a New Region of Nonconserved Amino Acids Critical for P110α Inhibition. Mol. Pharmacol..

[71] Zheng Z., Amran S.I., Zhu J., Schmidt-Kittler O., Kinzler K.W., Vogelstein B., Shepherd P.R., Thompson P.E., Jennings I.G. (2012). Definition of the Binding Mode of a New Class of Phosphoinositide 3-Kinase α-Selective Inhibitors Using In Vitro Mutagenesis of Non-Conserved Amino Acids and Kinetic Analysis. Biochem. J..

[72] Frazzetto M., Suphioglu C., Zhu J., Schmidt-Kittler O., Jennings I.G., Cranmer S.L., Jackson S.P., Kinzler K.W., Vogelstein B., Thompson P.E. (2008). Dissecting Isoform Selectivity of PI3K Inhibitors: The Role of Non-Conserved Residues in the Catalytic Pocket. Biochem. J..

[73] Chen P., Deng Y.-L., Bergqvist S., Falk M.D., Liu W., Timofeevski S., Brooun A. (2014). Engineering of an Isolated P110α Subunit of PI3Kα Permits Crystallization and Provides a Platform for Structure-Based Drug Design. Protein Sci..

[74] Rathinaswamy M.K., Gaieb Z., Fleming K.D., Borsari C., Harris N.J., Moeller B.E., Wymann M.P., Amaro R.E., Burke J.E. (2021). Disease-Related Mutations in PI3Kγ Disrupt Regulatory C-Terminal Dynamics and Reveal a Path to Selective Inhibitors. eLife.

[75] Iselt M., Holtei W., Hilgard P. (1989). The Tetrazolium Dye Assay for Rapid in Vitro Assessment of Cytotoxicity. Arzneimittelforschung.

[76] Mruk D.D., Cheng C.Y. (2011). Enhanced Chemiluminescence (ECL) for Routine Immunoblotting. Spermatogenesis.

[77] Morris G.M., Huey R., Lindstrom W., Sanner M.F., Belew R.K., Goodsell D.S., Olson A.J. (2009). AutoDock4 and AutoDockTools4: Automated Docking with Selective Receptor Flexibility. J. Comput. Chem..

[78] Forli S., Olson A.J. (2012). A Force Field with Discrete Displaceable Waters and Desolvation Entropy for Hydrated Ligand Docking. J. Med. Chem..

[79] Pettersen E.F., Goddard T.D., Huang C.C., Couch G.S., Greenblatt D.M., Meng E.C., Ferrin T.E. (2004). UCSF Chimera. A Visualization System for Exploratory Research and Analysis. J. Comput. Chem..

