# Supplementary material for: Synthesis, Molecular Docking, In Vitro and In Vivo Studies of Novel Dimorpholinoquinazoline-Based Potential Inhibitors of PI3K/Akt/mTOR Pathway
Source: Int J Mol Sci. 2022 Sep 17;23(18):10854. doi: 10.3390/ijms231810854 (PMC9503112; doi:10.3390/ijms231810854)
Supplement: Supplementary file 1 [file ijms-23-10854-s001.zip › ijms-1915210-supplementary.pdf]

Supporting information for

Synthesis, molecular docking, *in vitro* and *in vivo* studies of novel dimorpholinoquinazoline-based potential inhibitors of PI3K/Akt/mTOR pathway

Maria V. Zapevalova[a], Ekaterina S. Shchegravina[a,b], Irina P. Fonareva[a], Diana I. Salnikova[c], Danila V. Sorokin[c], Alexander M. Scherbakov[c], Alexander A. Maleev[a], Stanislav K. Ignatov[a], Ivan D. Grishin[a], Alexander N. Kuimov[d], Maryia V. Konovalova[e], Elena V. Svirshchevskaya[e] and Alexey Yu. Fedorov*[a,b]*

[a] *Department of Organic Chemistry, Nizhny Novgorod State University, Gagarina av. 23, Nizhny Novgorod 603950, Russian Federation.*

[b] *N.D. Zelinsky Insitute of Organic Chemistry RAS, Leninsky Prospect 47, Moscow 119991, Russian Federation.*

[c] *Department of Experimental Tumor Biology, Blokhin N.N. National Medical Research Center of Oncology, Kashirskoye sh. 24, Moscow 115522, Russian Federation.*

[d] *A.N. Belozersky Institute of Physico-Chemical Biology, M.V.Lomonosov Moscow State University, Leninskye gory, house 1, building 40, 119992 Moscow, Russian Federation*

[e] *Department of Immunology, Shemyakin-Ovchinnikov Institute of Bioorganic Chemistry RAS, Miklukho-Maklaya 16/10, Moscow 117997, Russian Federation*

* Corresponding authors: sc.katarina@yandex.ru (E.S.S.), afedorovNN@yandex.ru (A.Yu.F.)

Table of contents

NMR spectra of compounds 2-6; 7a-l ……………………………………………………. …..S3

Predicted ADME features of target compounds……………………………….……….. …..S22

Illustrations for docking studies ………………………………………………………… …..S23

Figure S1………………………………………………………………………………… …..S25

HPLC chromatogram for **7c**……………………………………………………………….. …..S26

NMR spectra for compounds 2-6, 7a-l

2-amino-4-bromobenzoic acid **2**

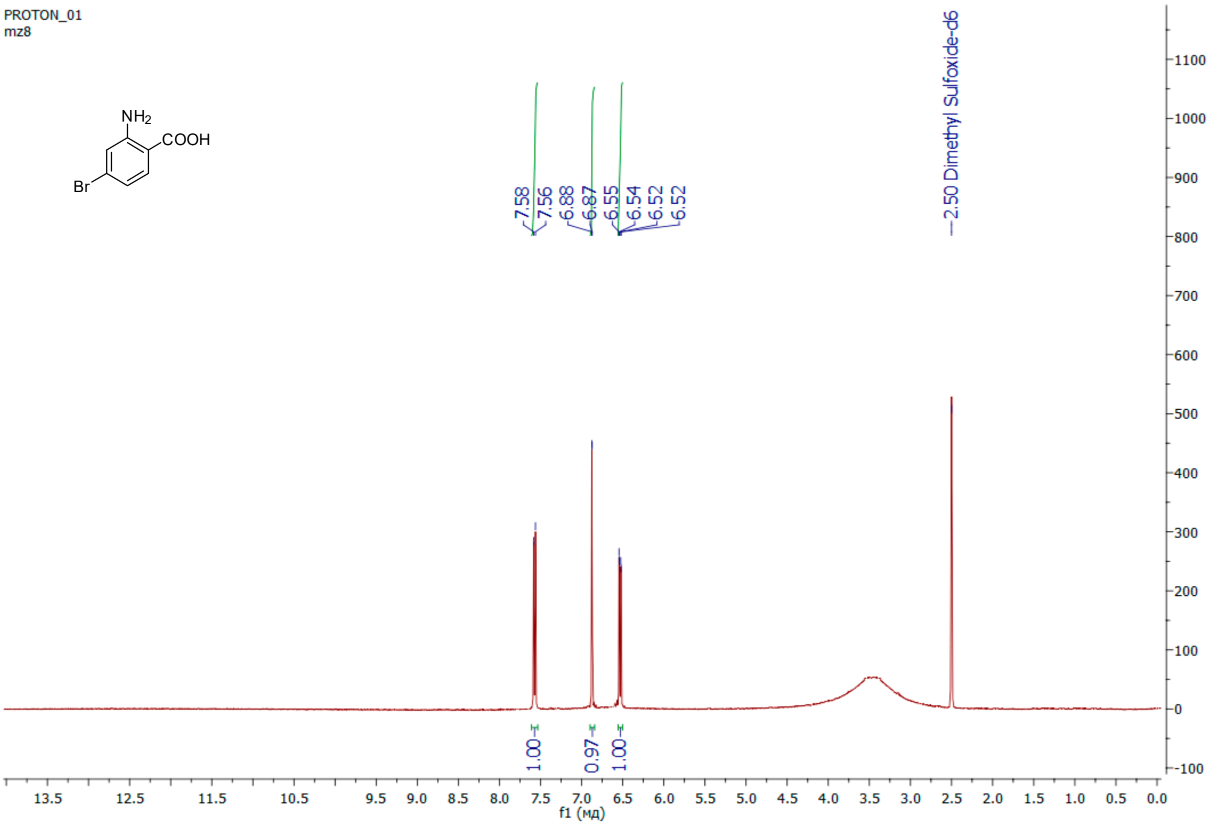

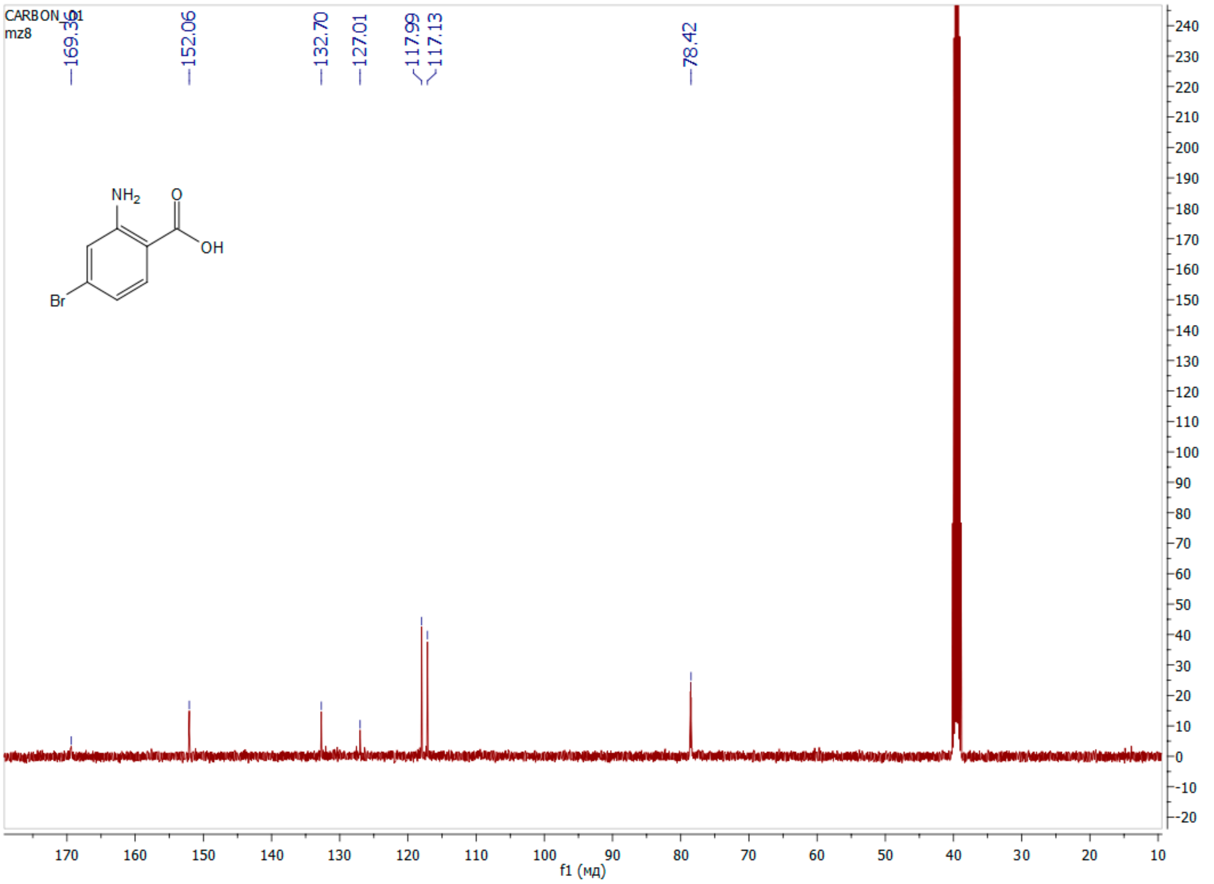

7-bromoquinazoline-2,4(1H,3H)-dione **3**

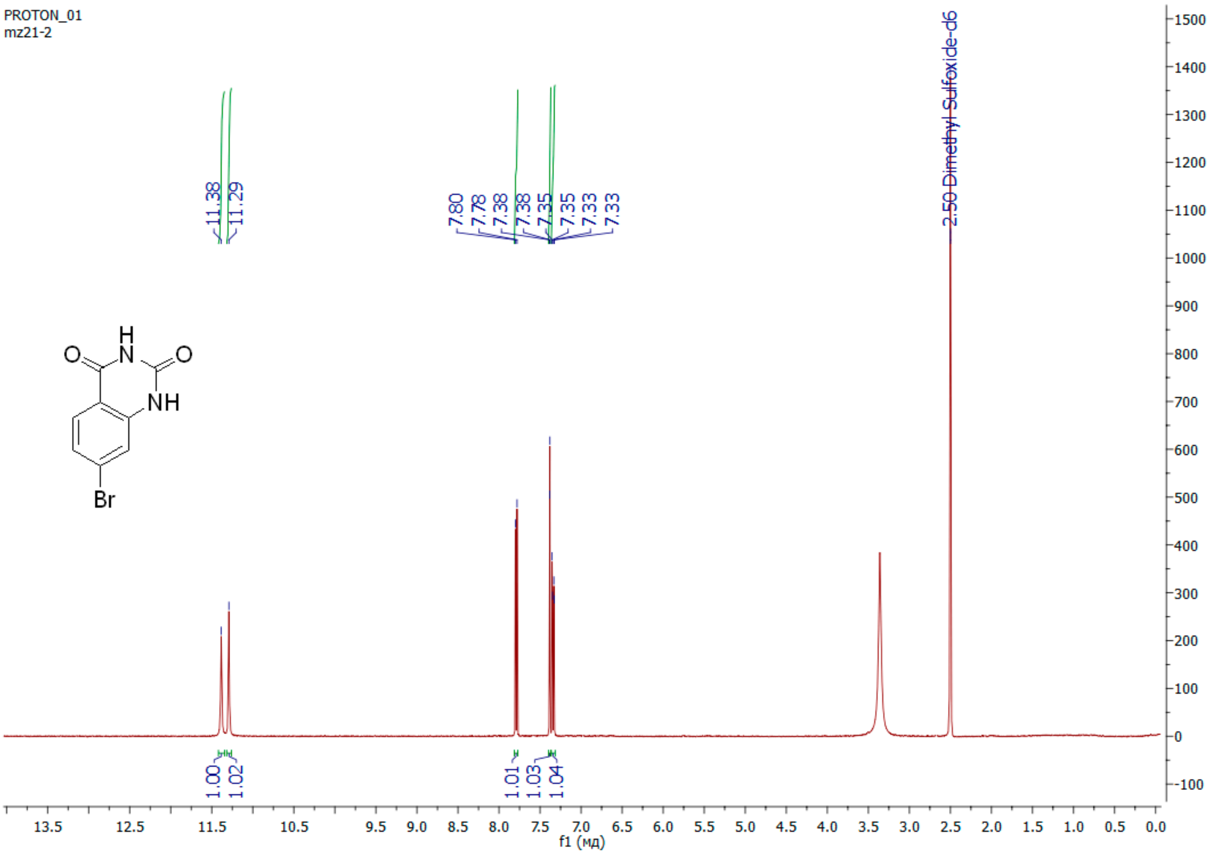

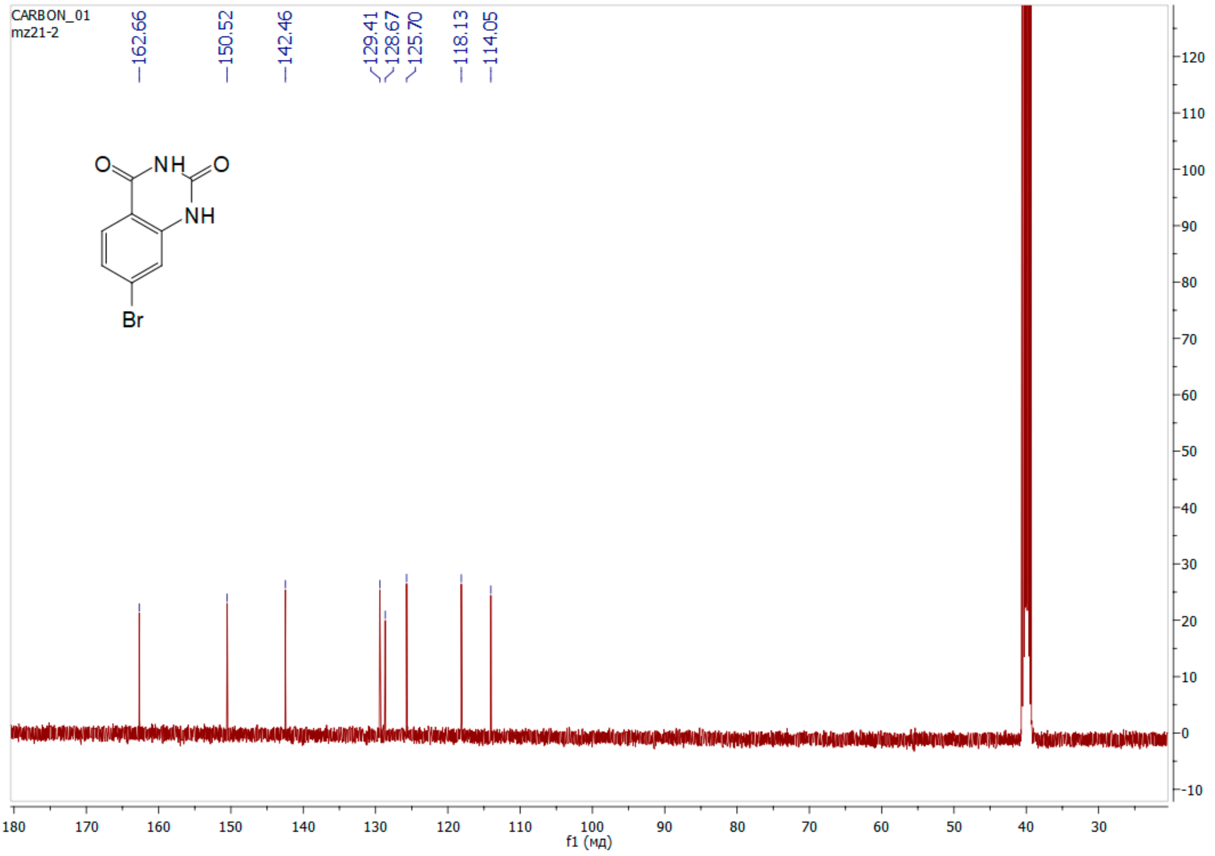

7-bromo-2,4-dichloroquinazoline

4

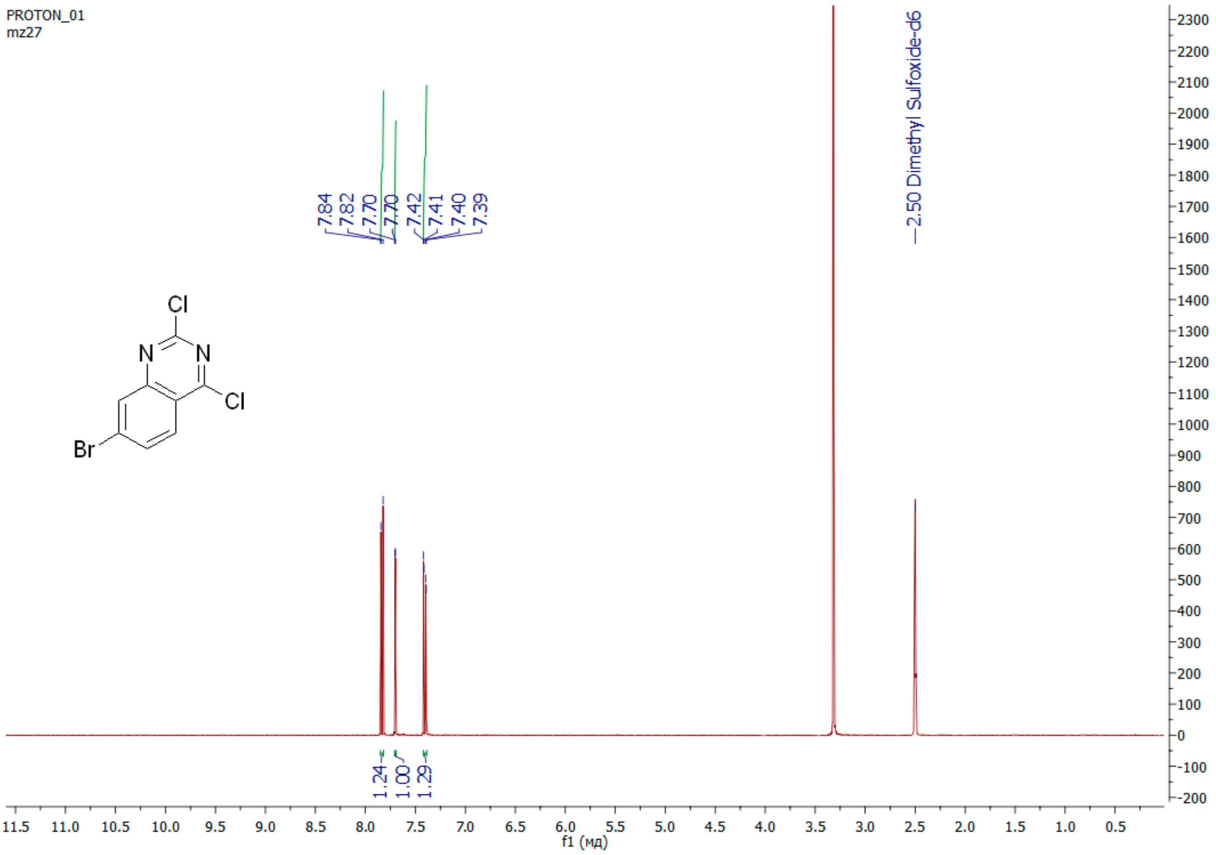

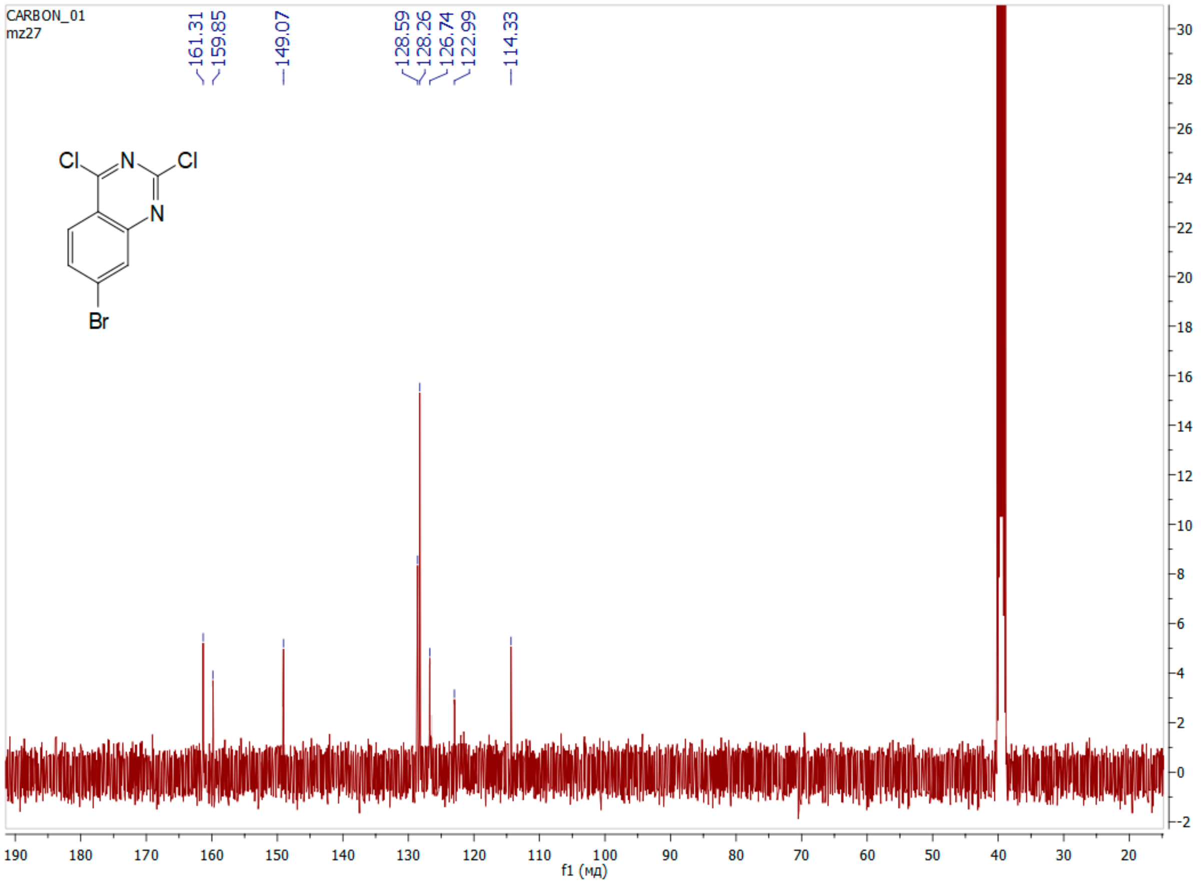

4,4'-(7-bromoquinazoline-2,4-diyl)dimorpholine

5

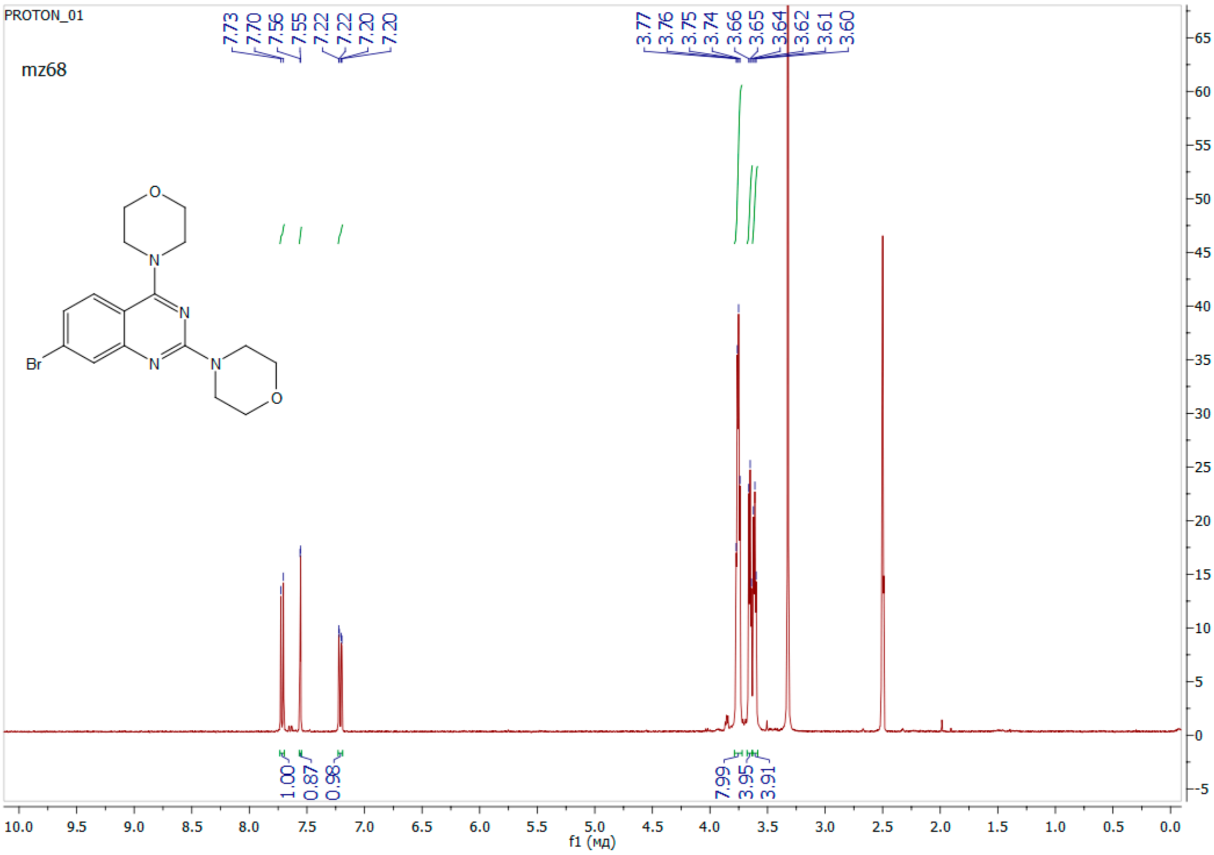

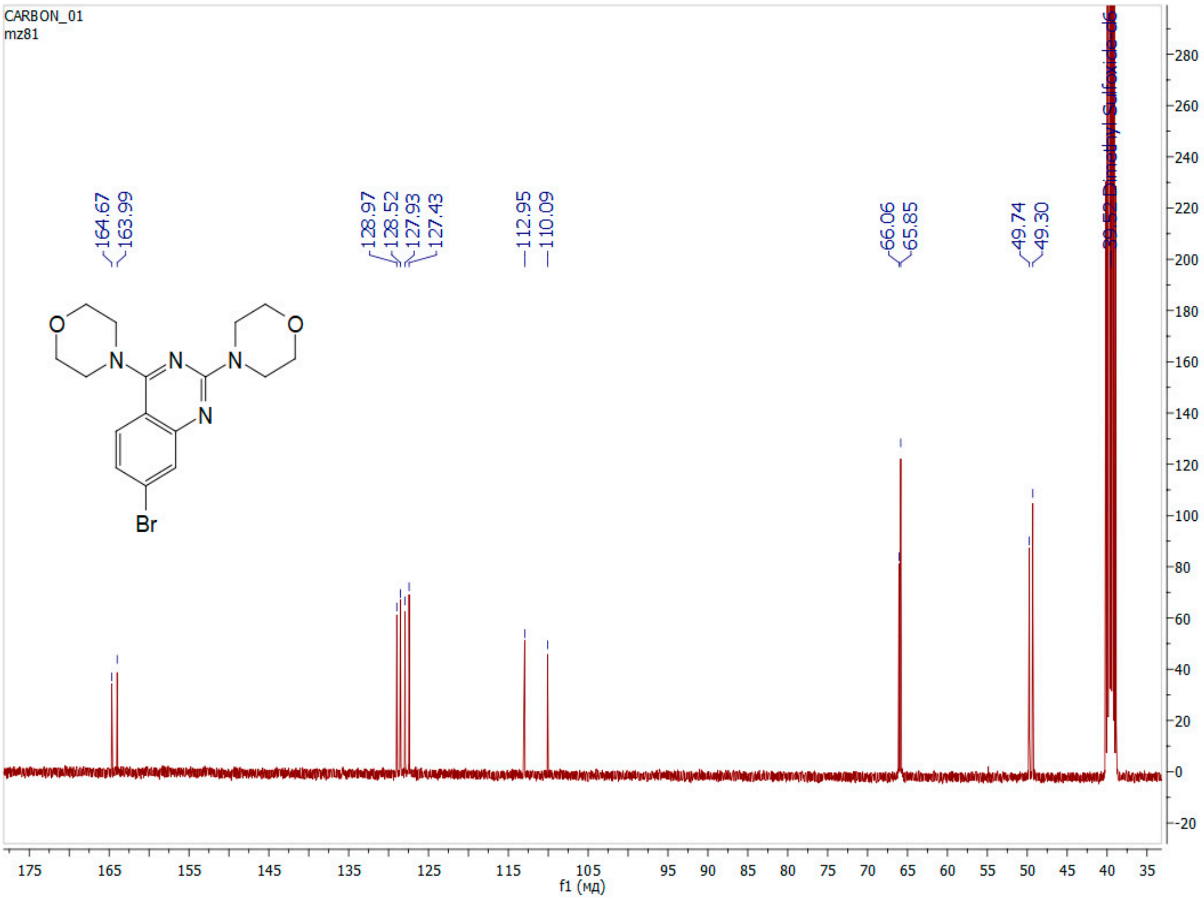

tert-butyl (4-(2,4-dimorpholinoquinazolin-7-yl)phenyl)carbamate

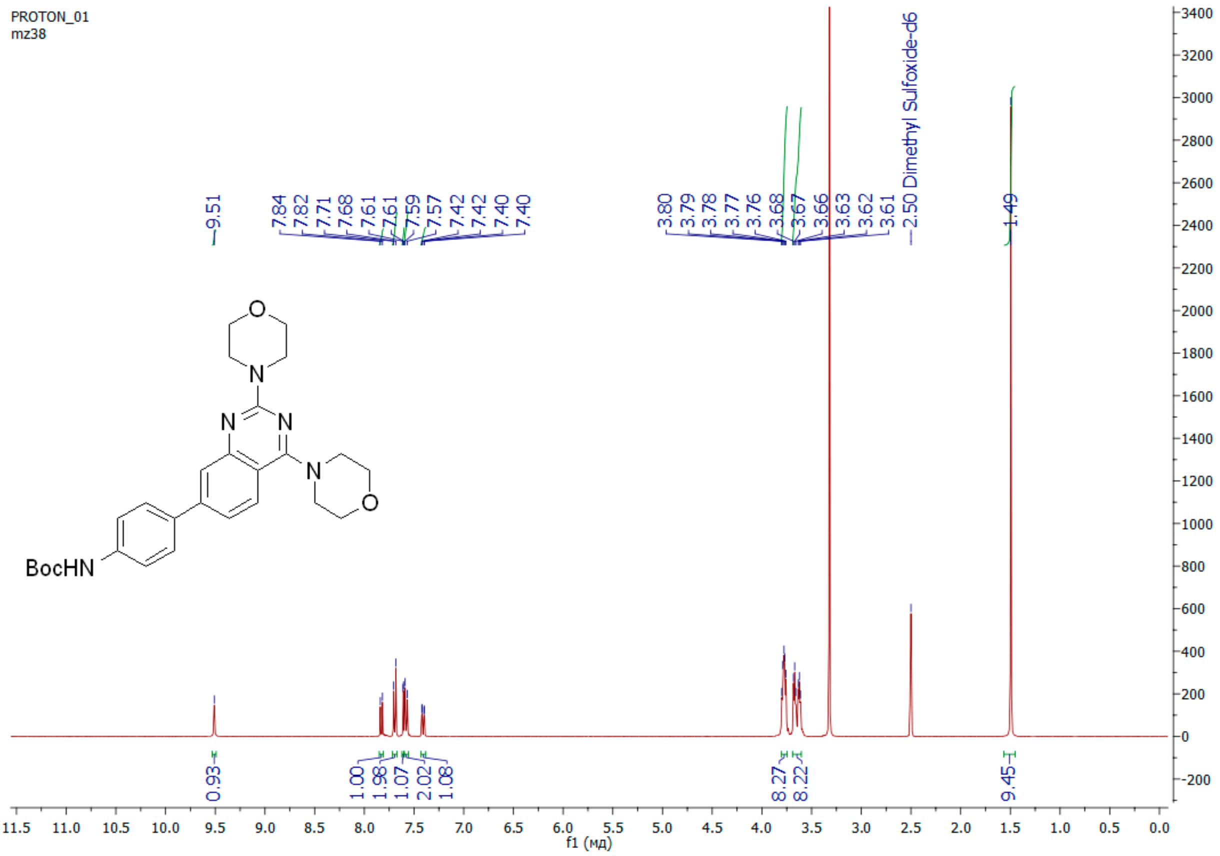

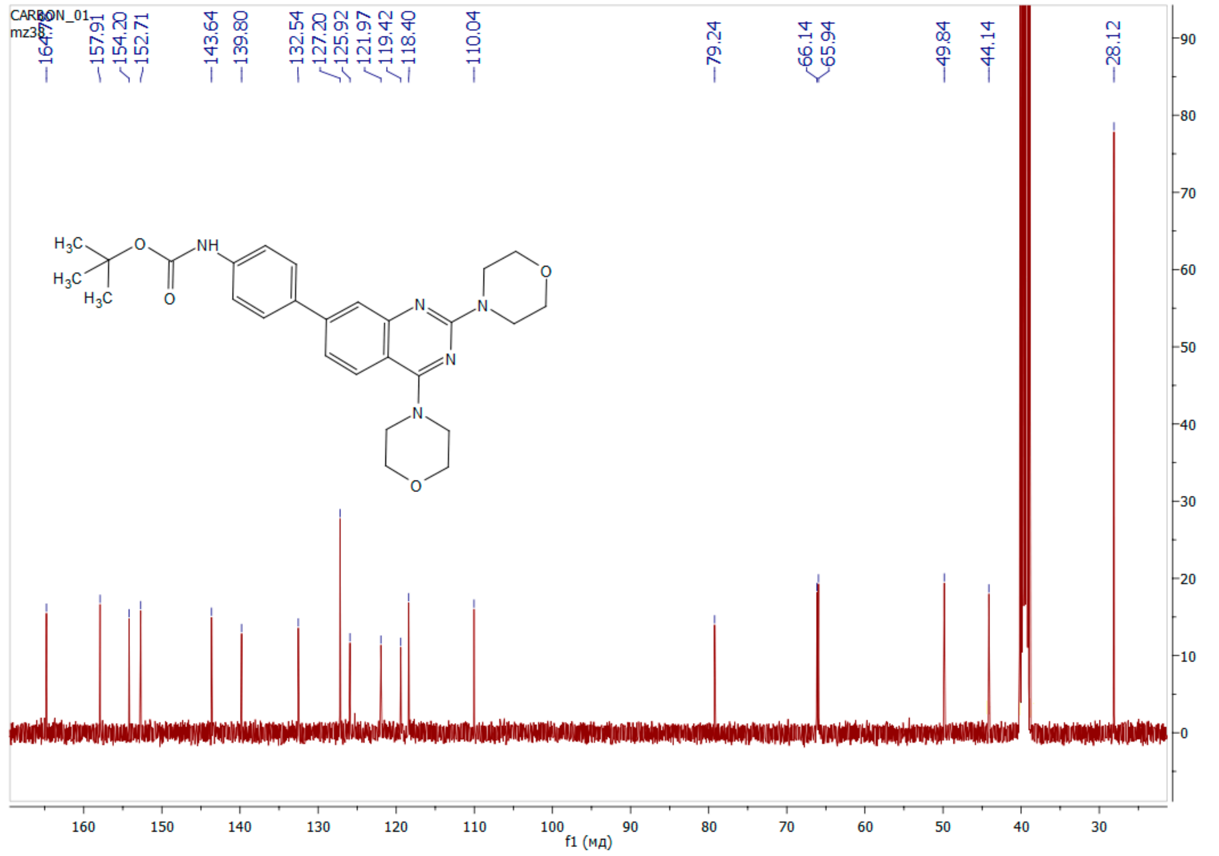

4-(2,4-dimorpholinoquinazolin-7-yl)aniline

6

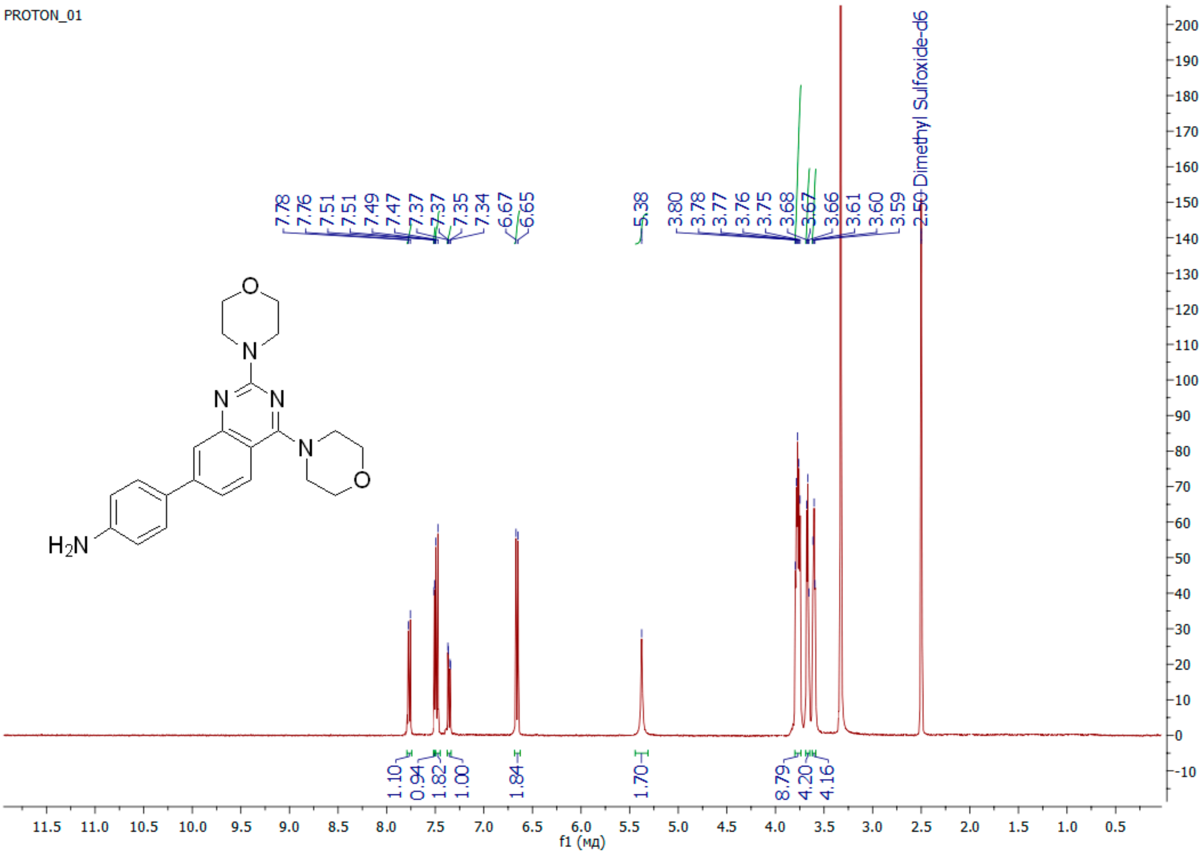

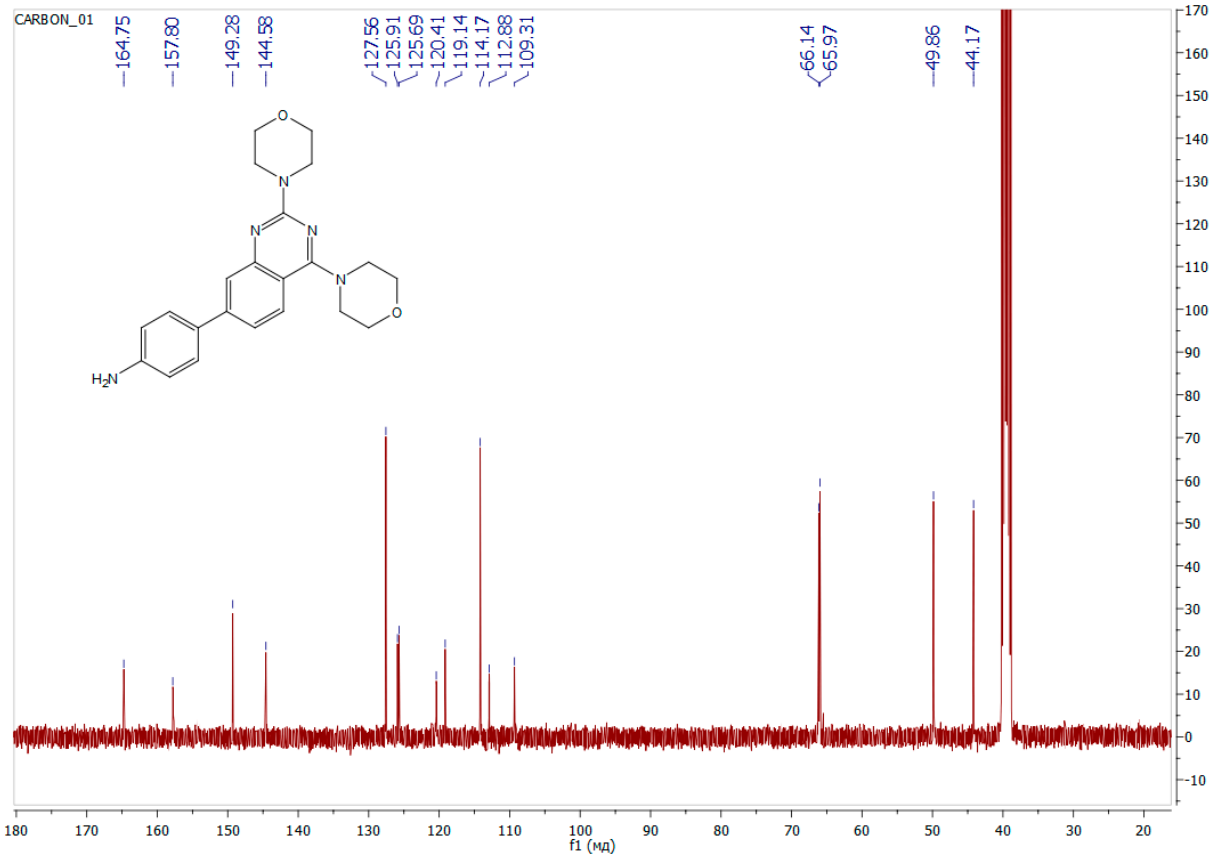

1-(4-(2,4-dimorpholinoquinazolin-7-yl)phenyl)-3-(furan-2-ylmethyl)urea **7a**

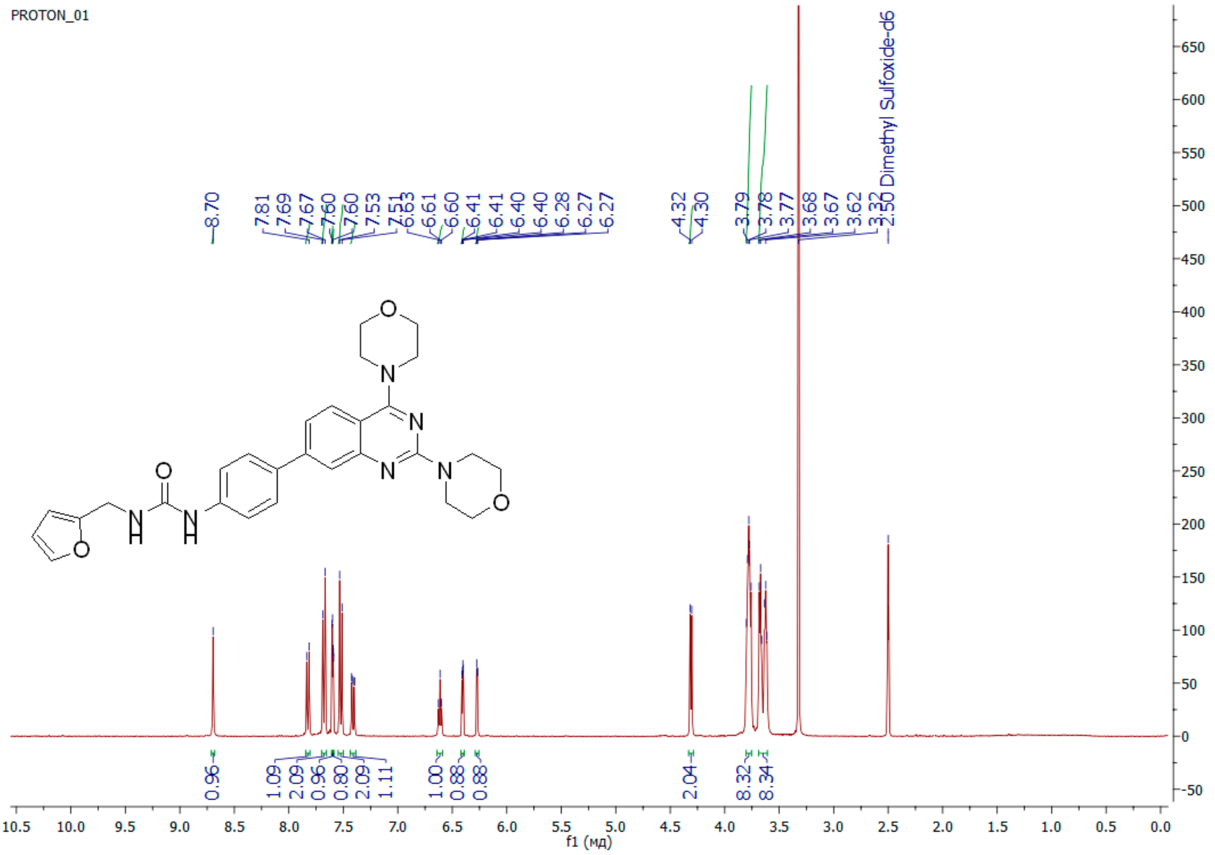

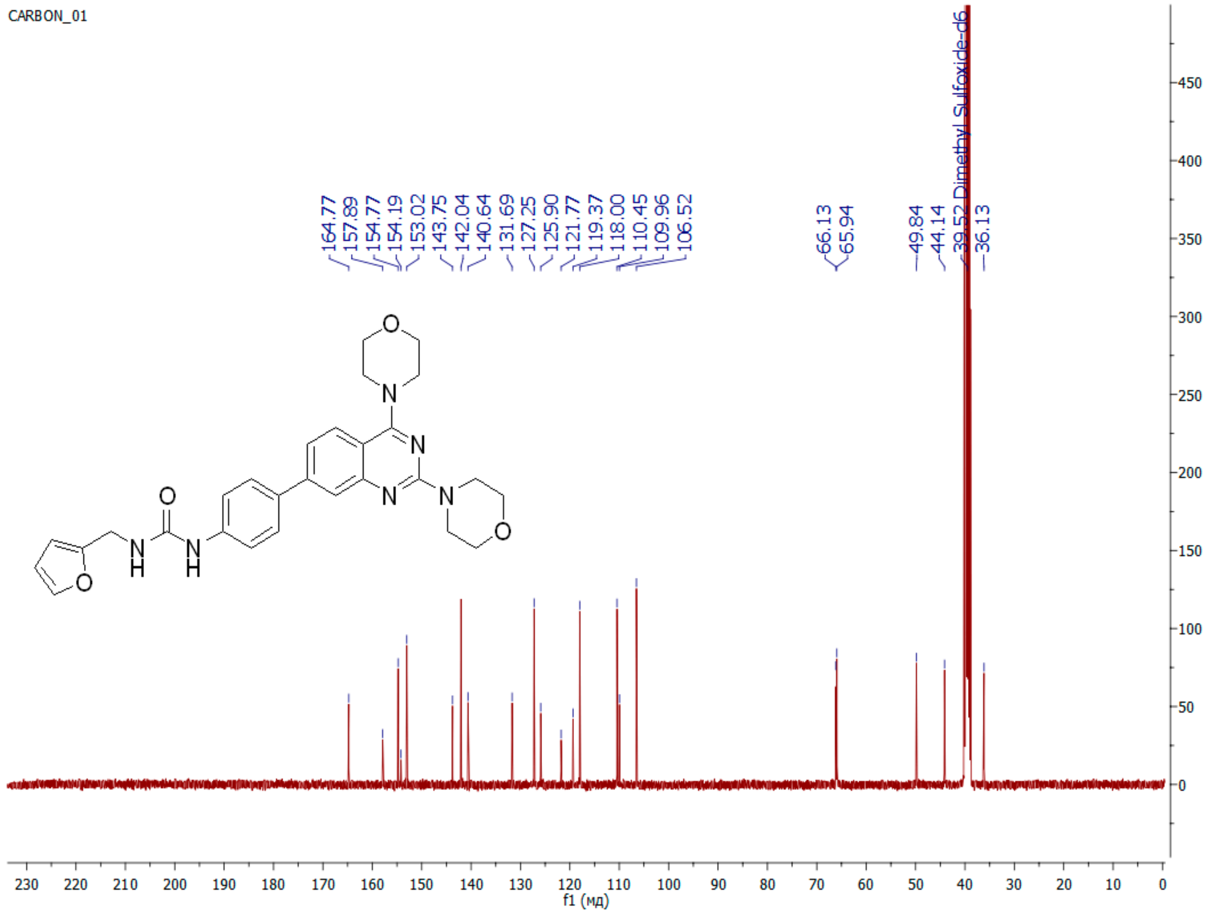

1-(4-(2,4-dimorpholinoquinazolin-7-yl)phenyl)-3-(4-methoxybenzyl)urea
7b

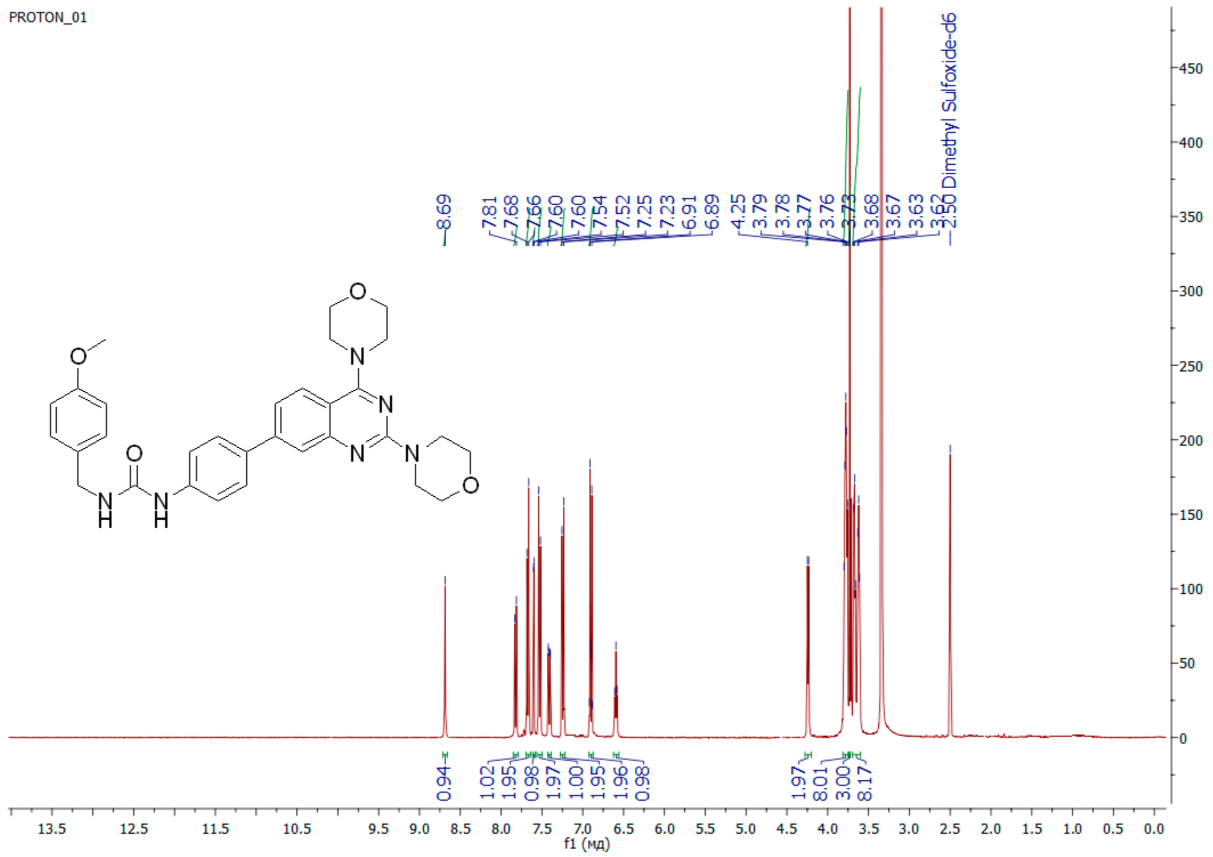

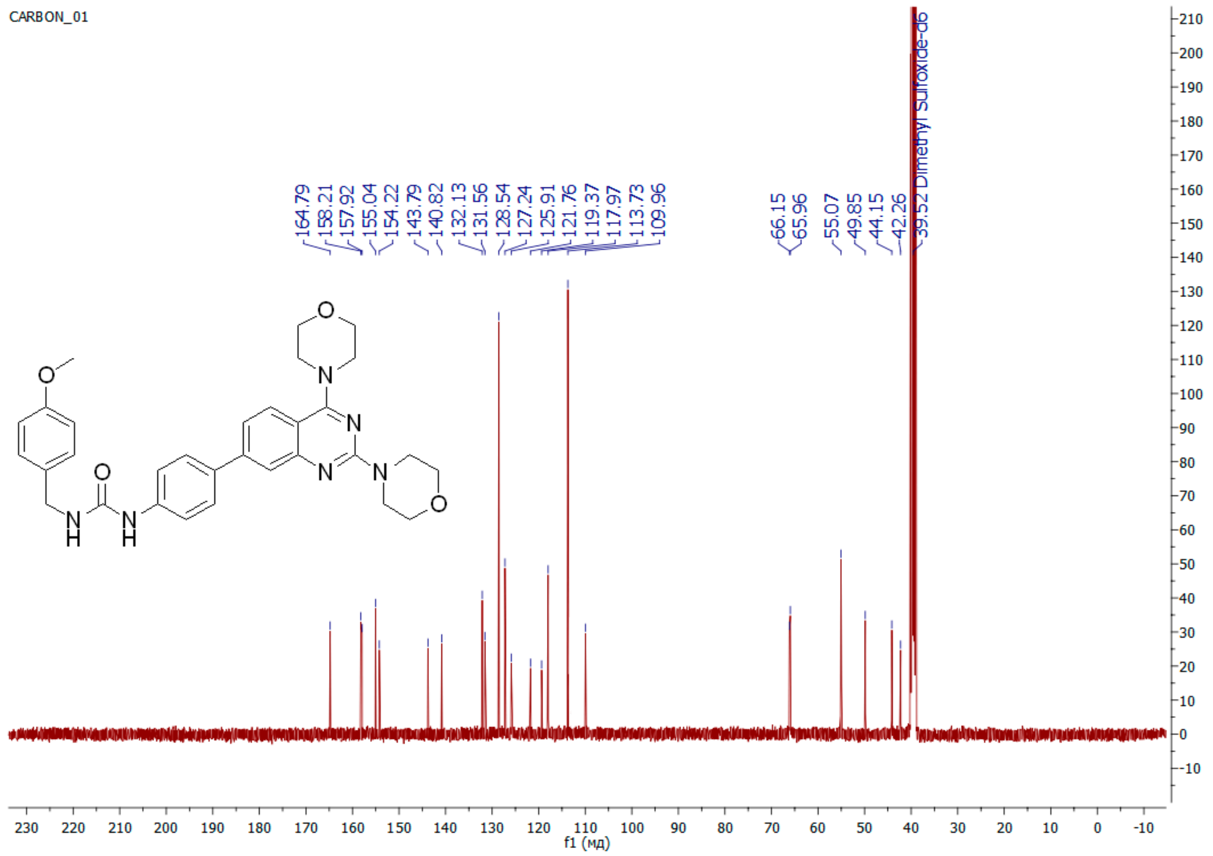

1-(4-(2,4-dimorpholinoquinazolin-7-yl)phenyl)-3-(2-(pyridin-3-yl)ethyl)urea **7c**

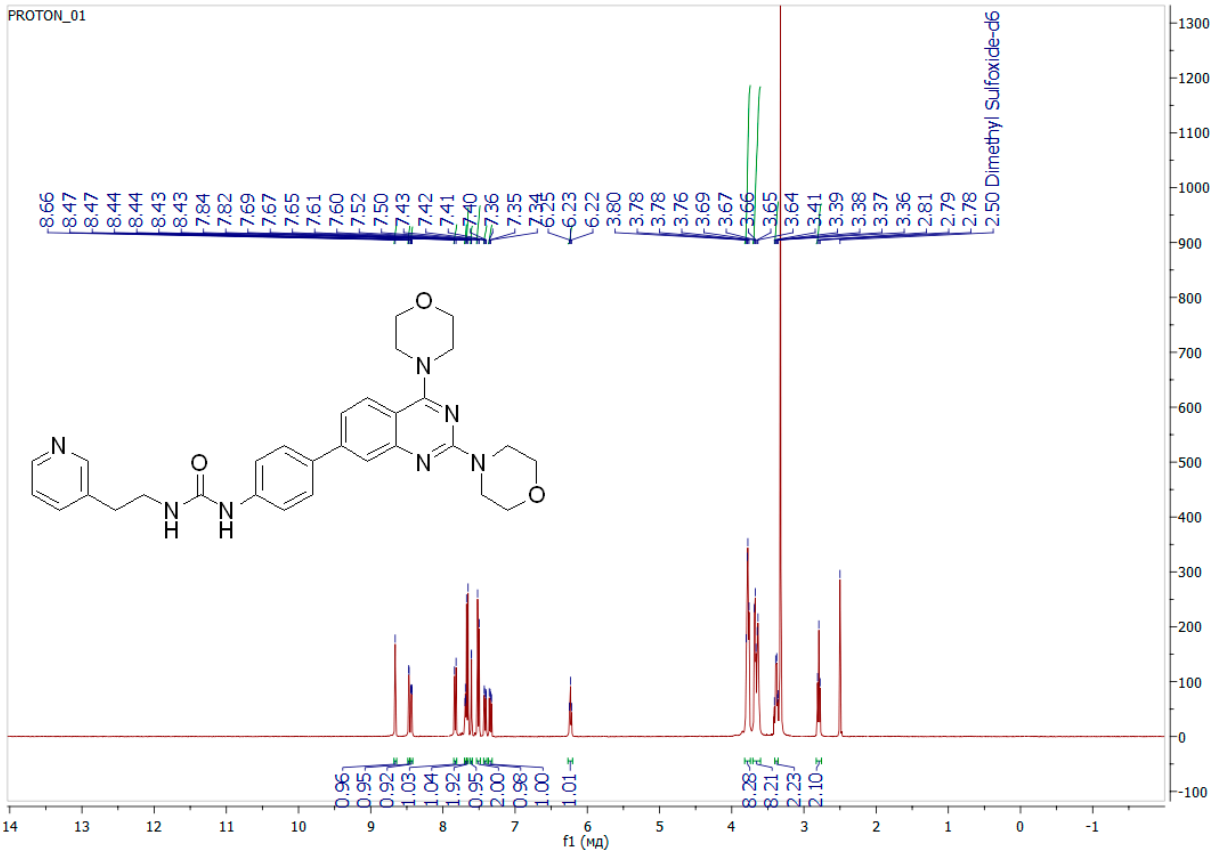

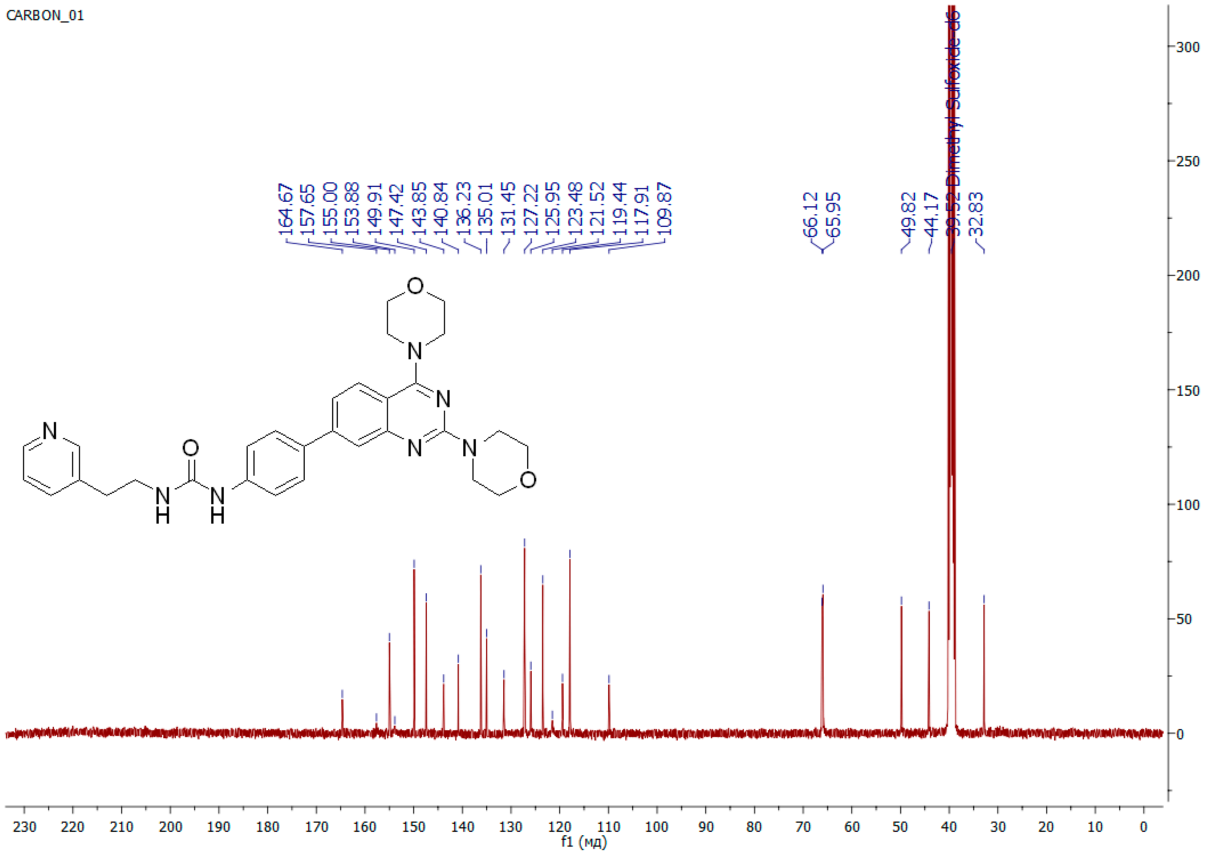

1-cyclopropyl-3-(4-(2,4-dimorpholinoquinazolin-7-yl)phenyl)urea
7d

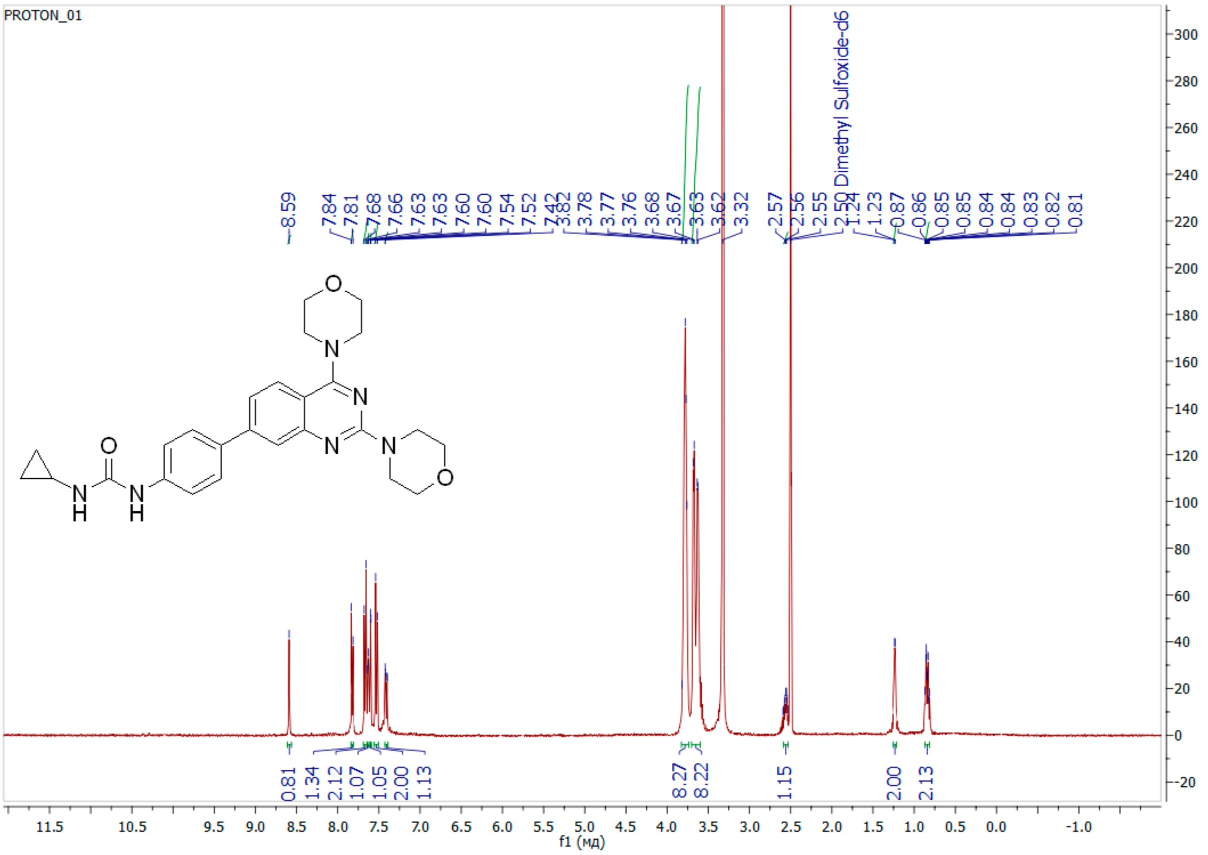

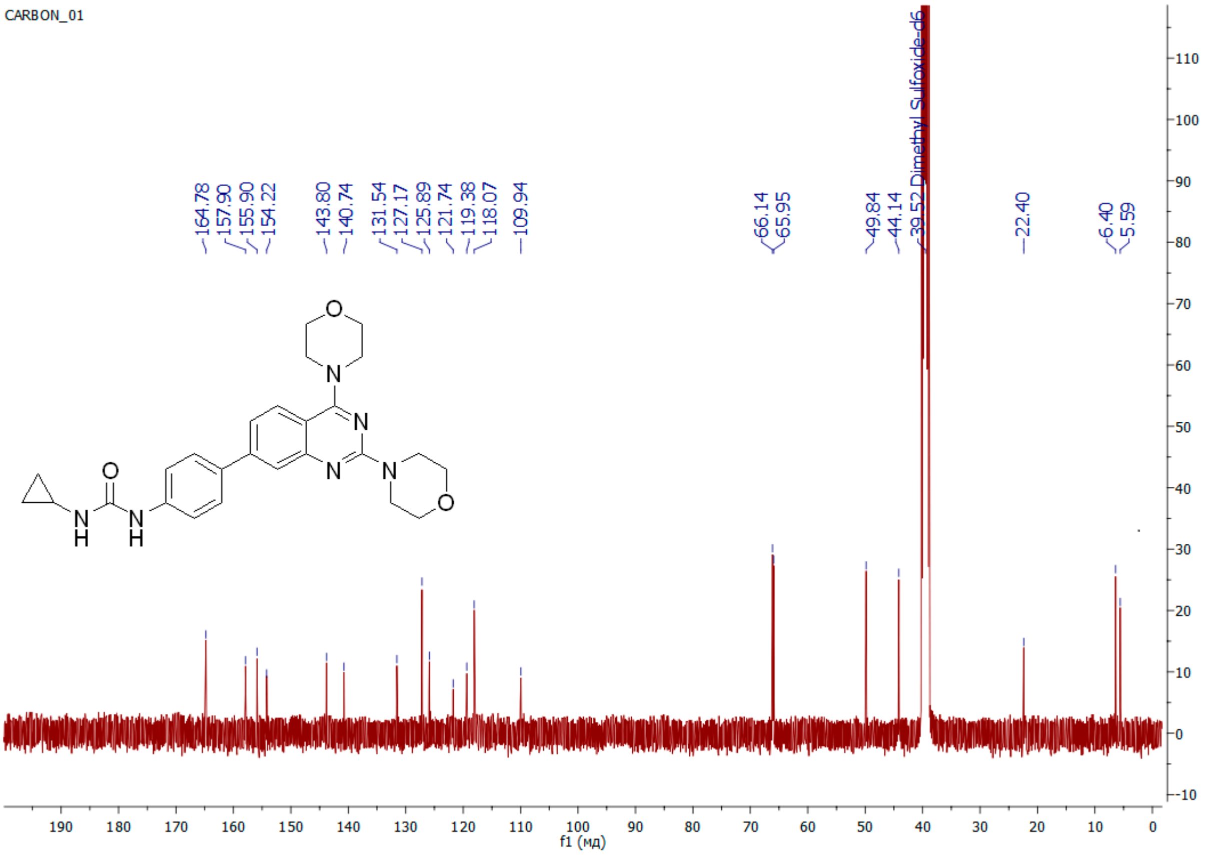

N6-((4-(2,4-dimorpholinoquinazolin-7-yl)phenyl)carbamoyl)lysine

7e

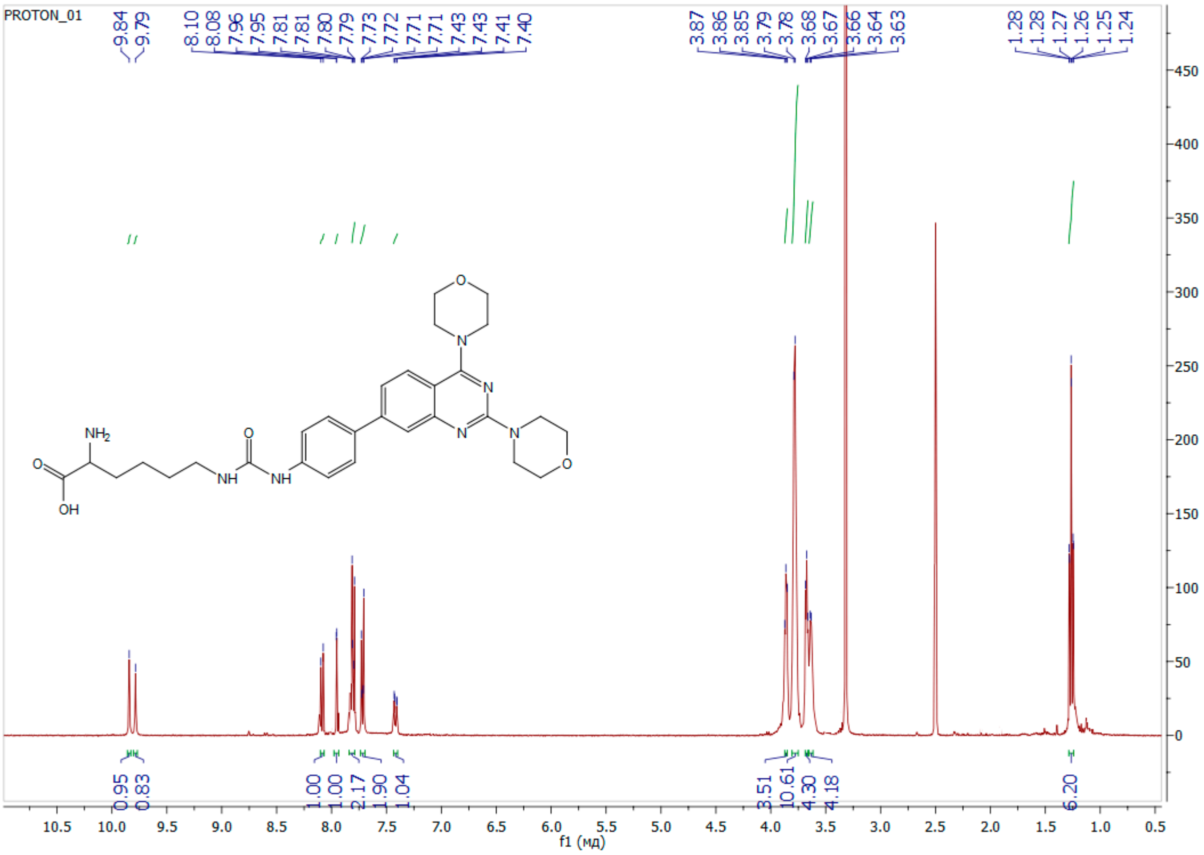

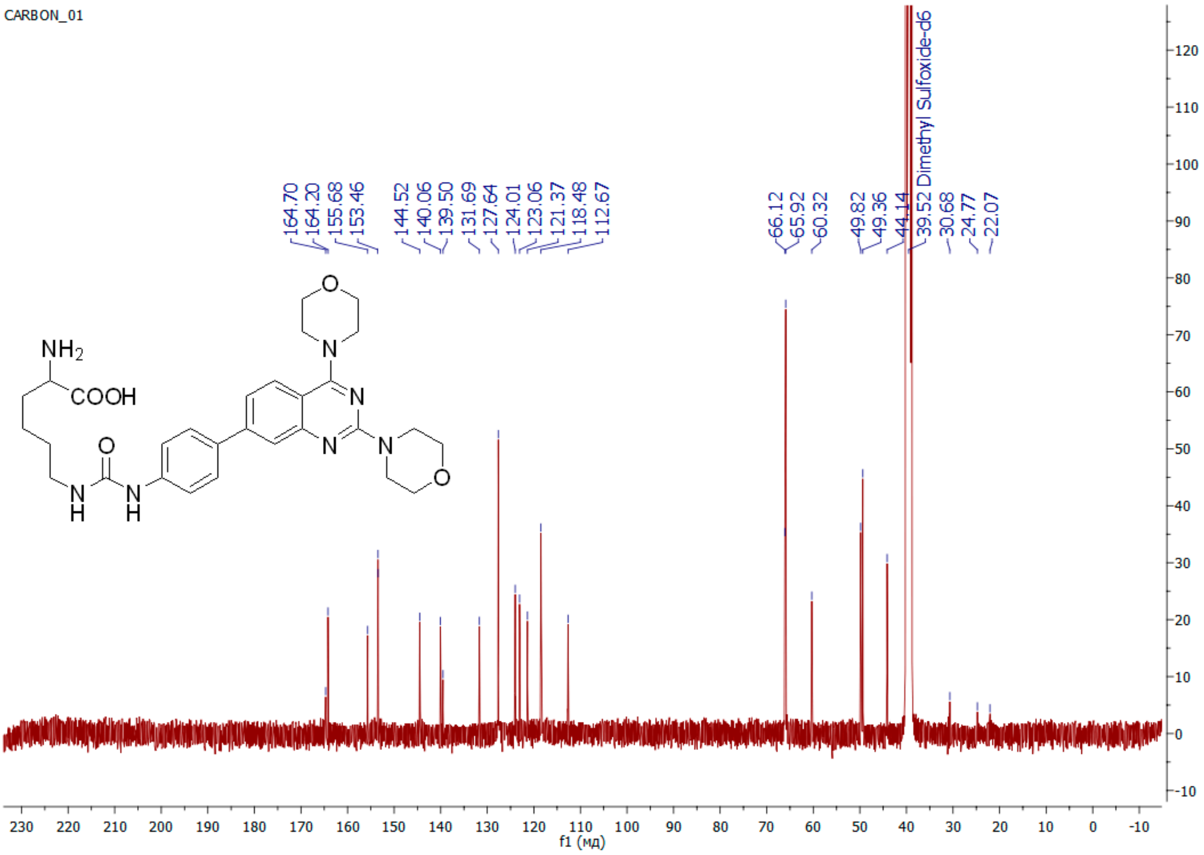

1-(4-(2,4-dimorpholinoquinazolin-7-yl)phenyl)-3-(4-nitrophenyl)urea

7f

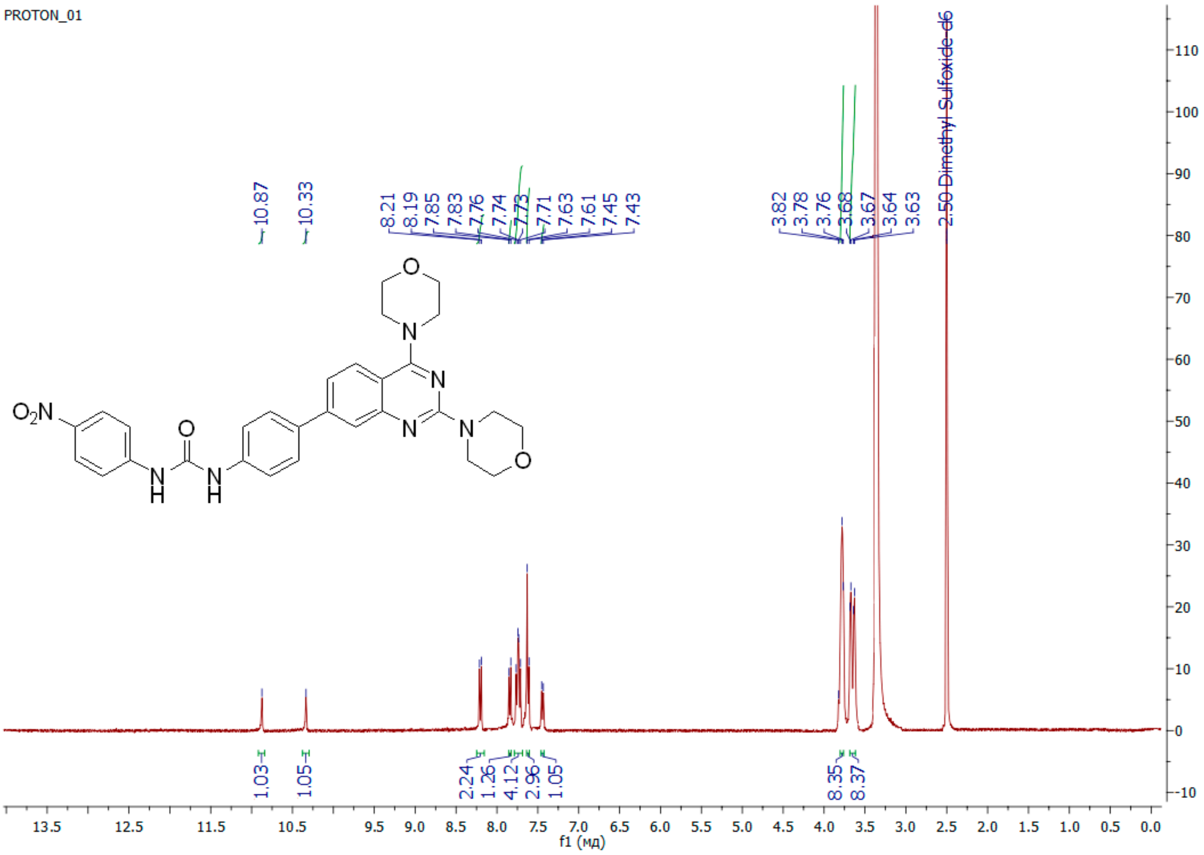

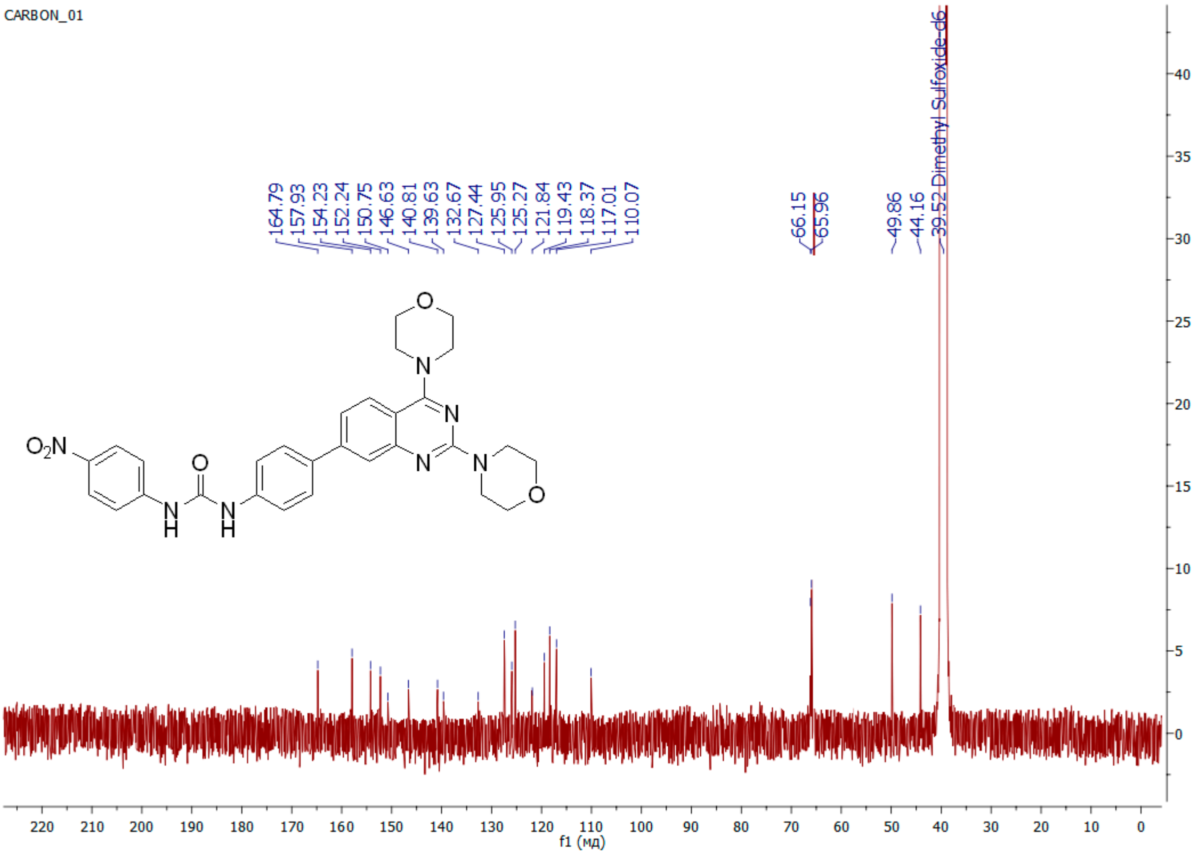

1-(4-(2,4-dimorpholinoquinazolin-7-yl)phenyl)-3-(4-fluorophenyl)urea
7g

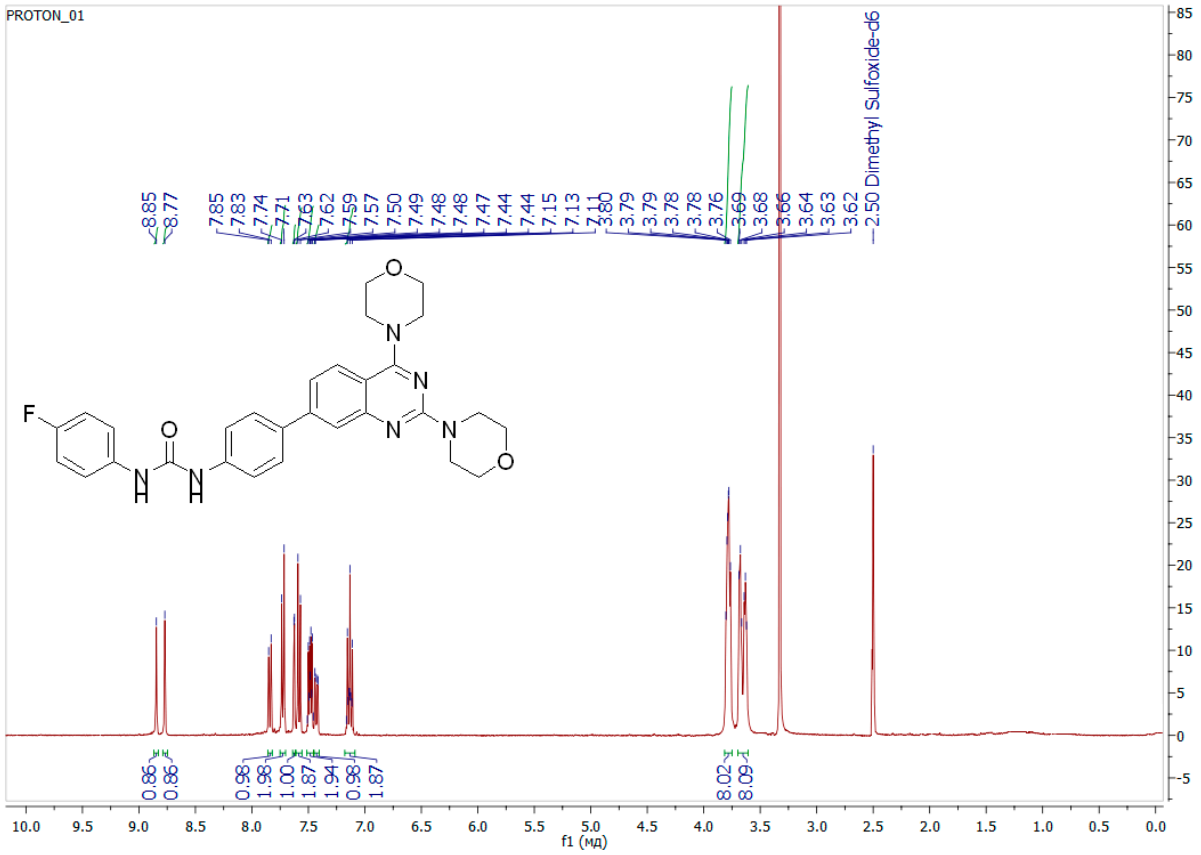

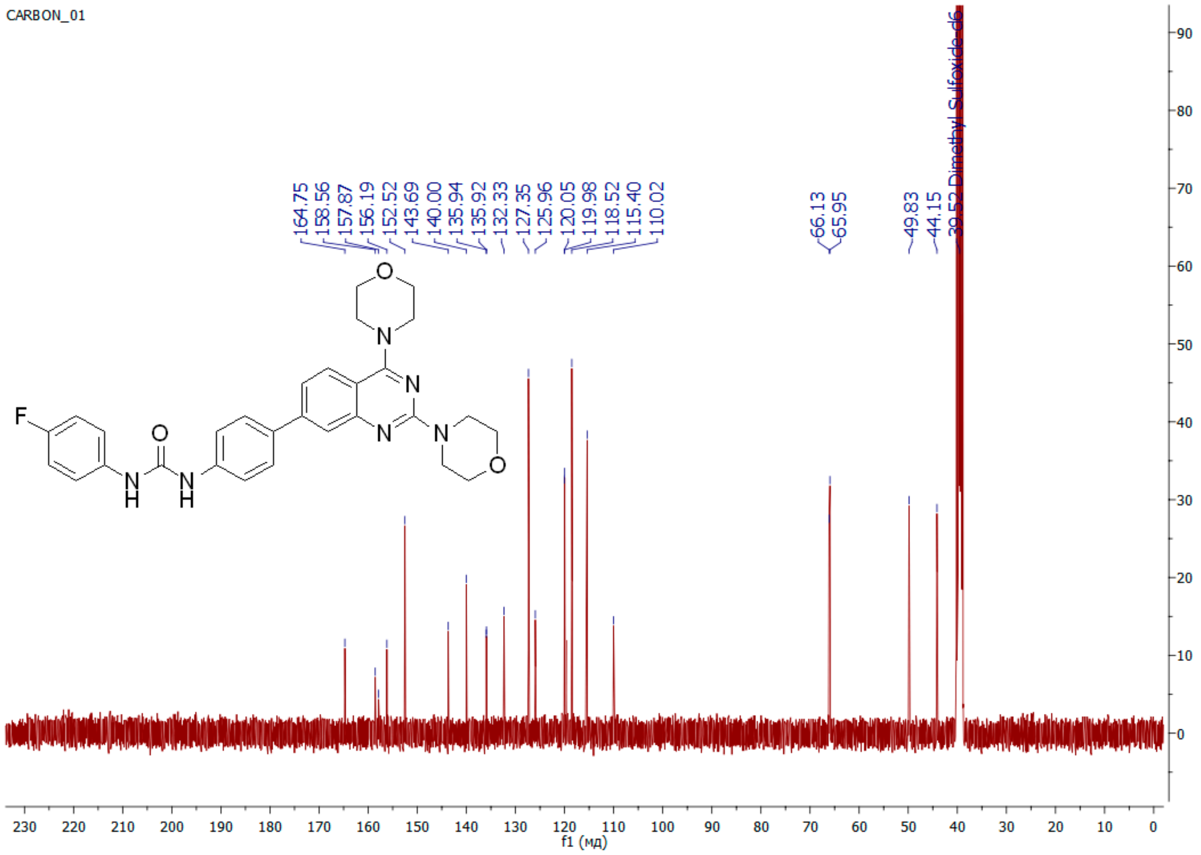

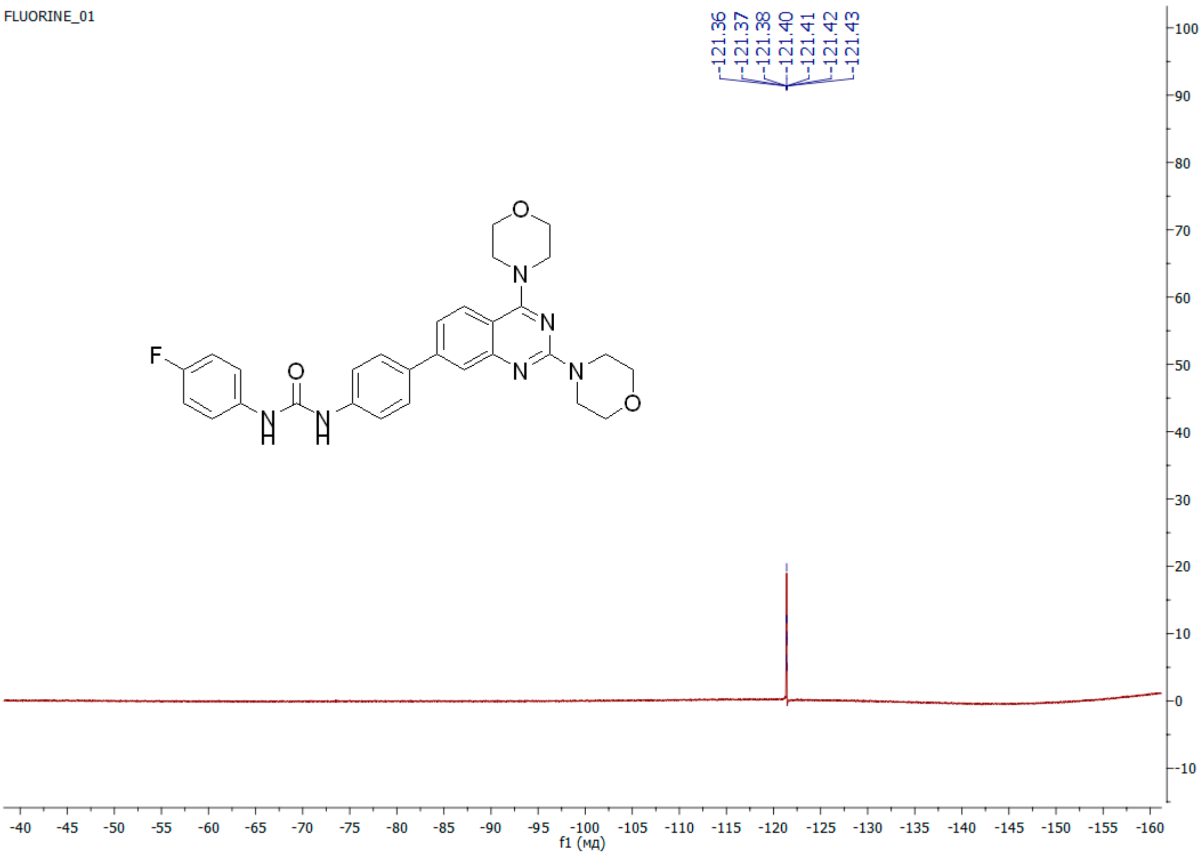
FLUORINE_01
-121.36
-121.37
-121.38
-121.40
-121.41
-121.42
-121.43
F
O
N
H
N
H
N
N
N
O
N
O
f1 (мд)

1-(4-(2,4-dimorpholinoquinazolin-7-yl)phenyl)-3-(pyridin-2-yl)urea
7h

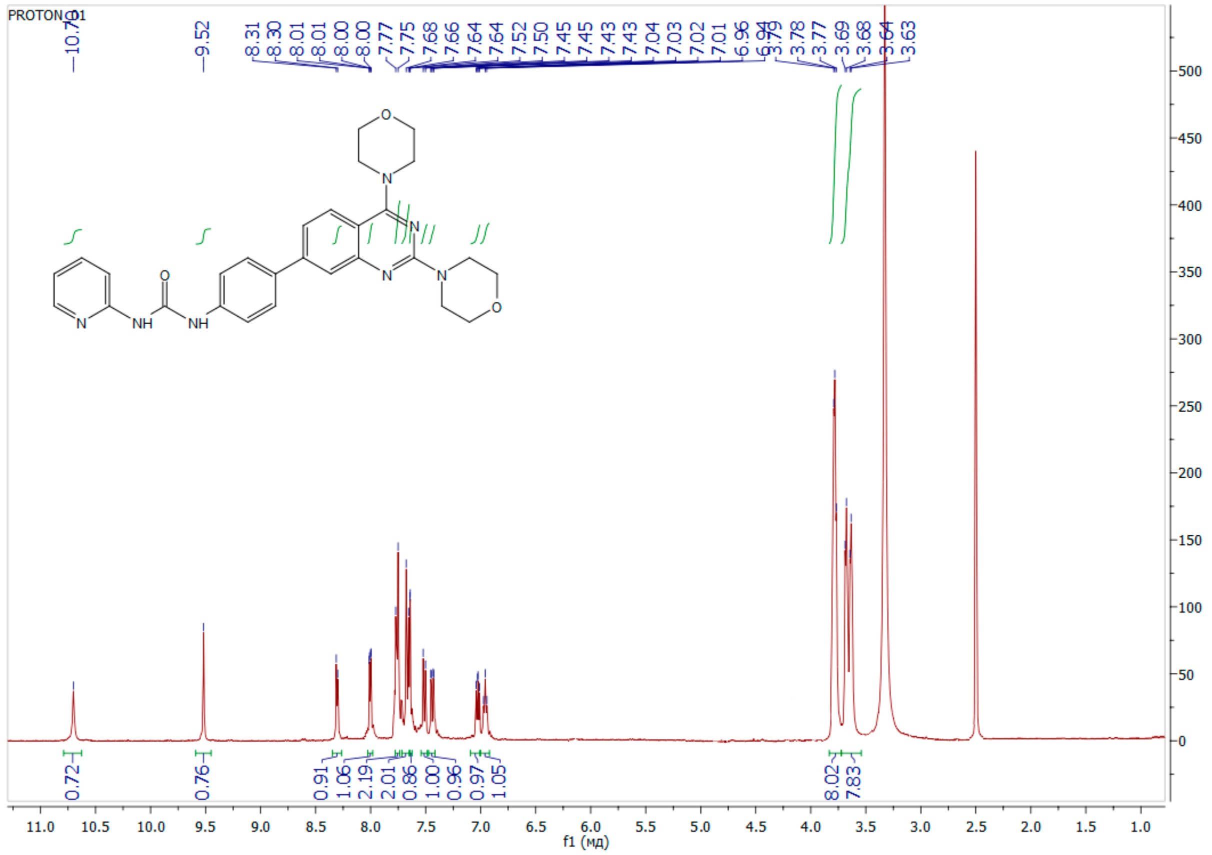

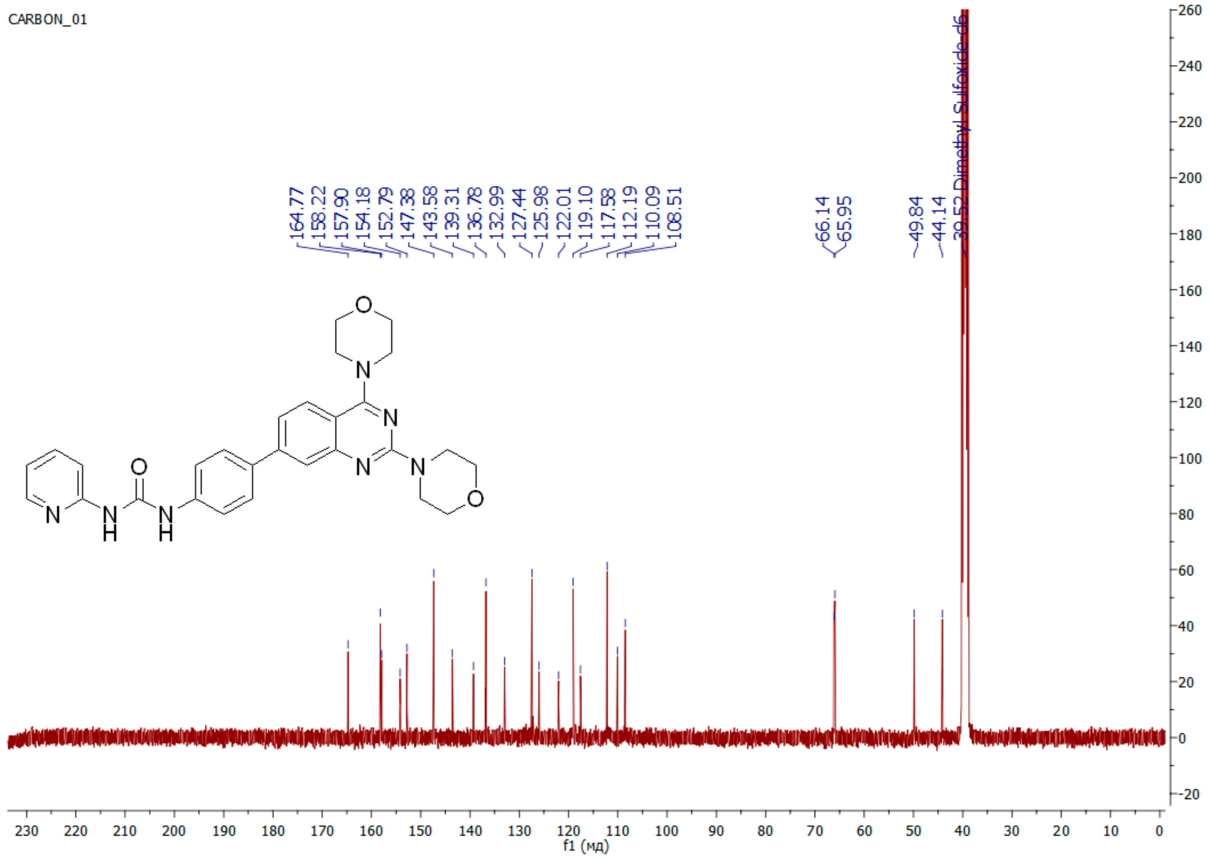

1-(4-(2,4-dimorpholinoquinazolin-7-yl)phenyl)-3-(naphthalen-1-yl)urea

7i

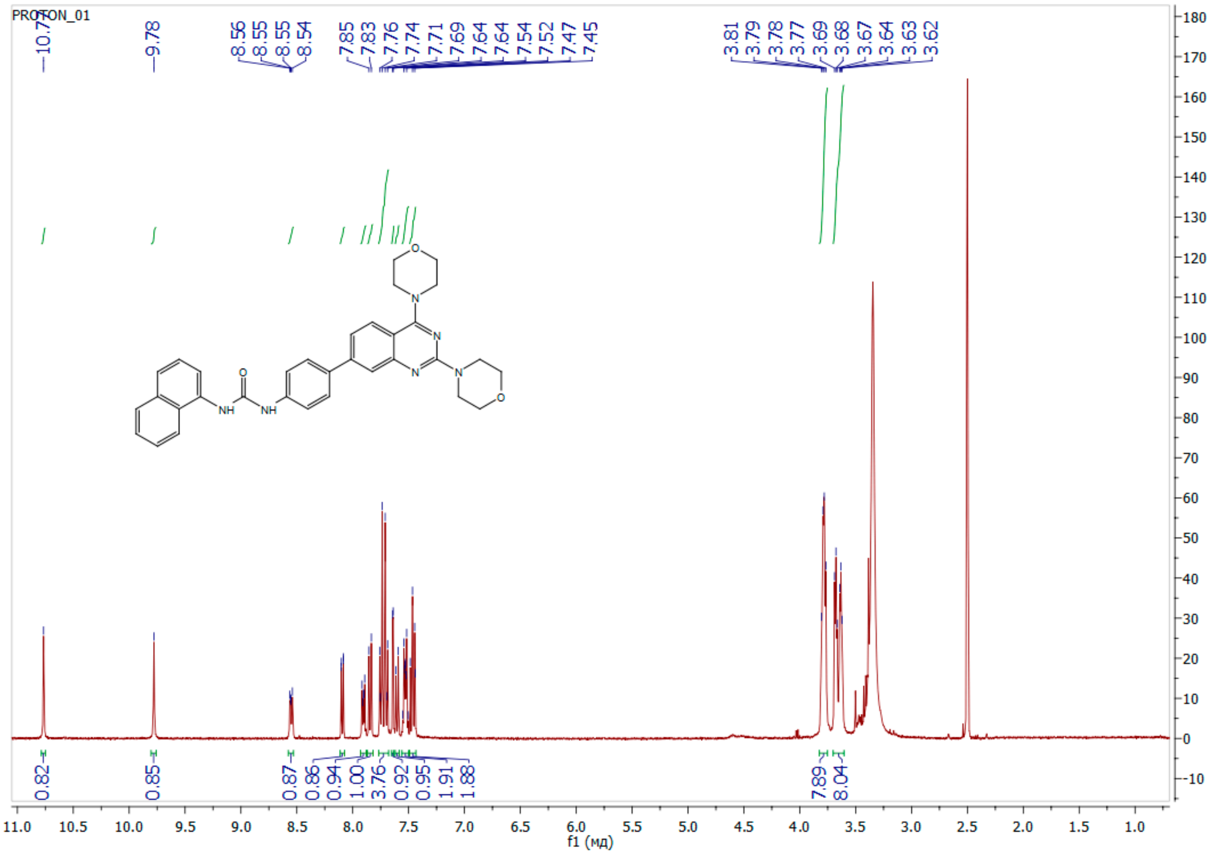

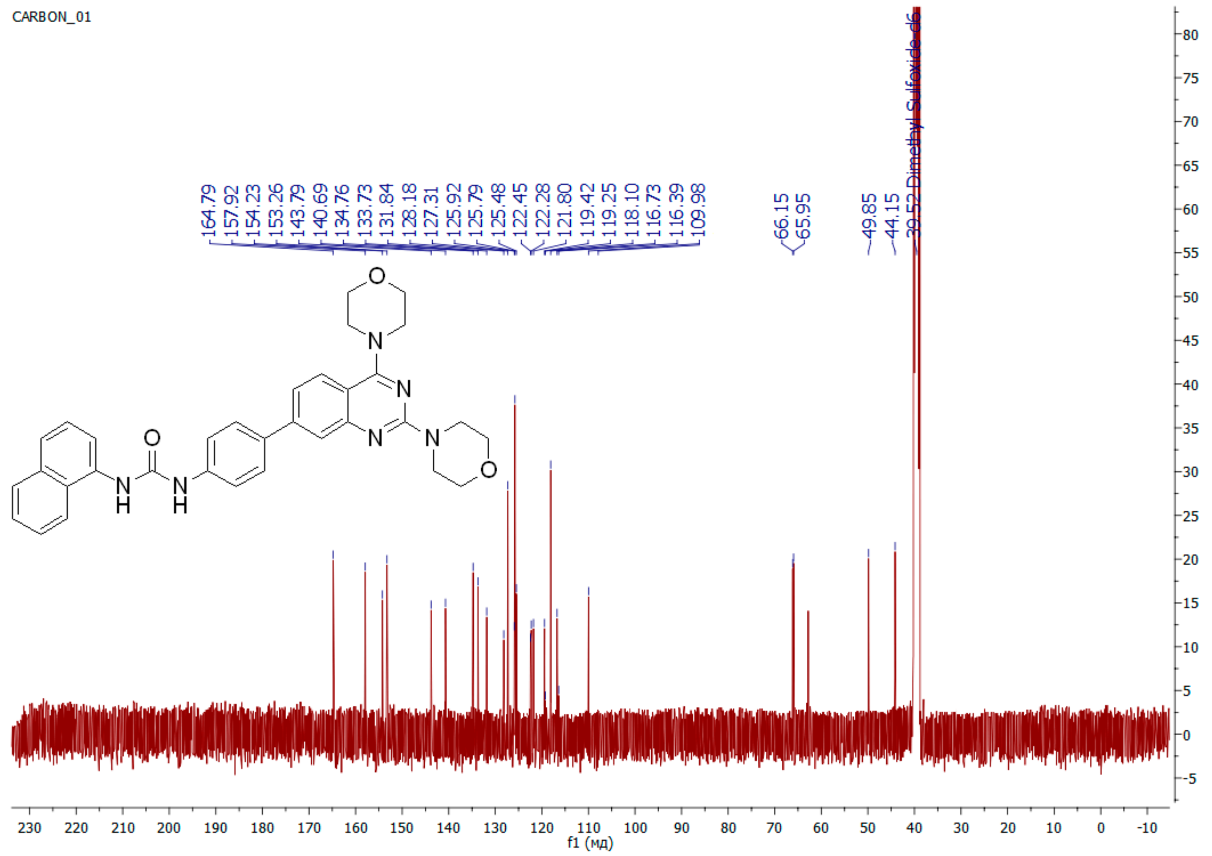

Methyl 4-(3-(4-(2,4-dimorpholinoquinazolin-7-yl)phenyl)ureido)benzoate

7j

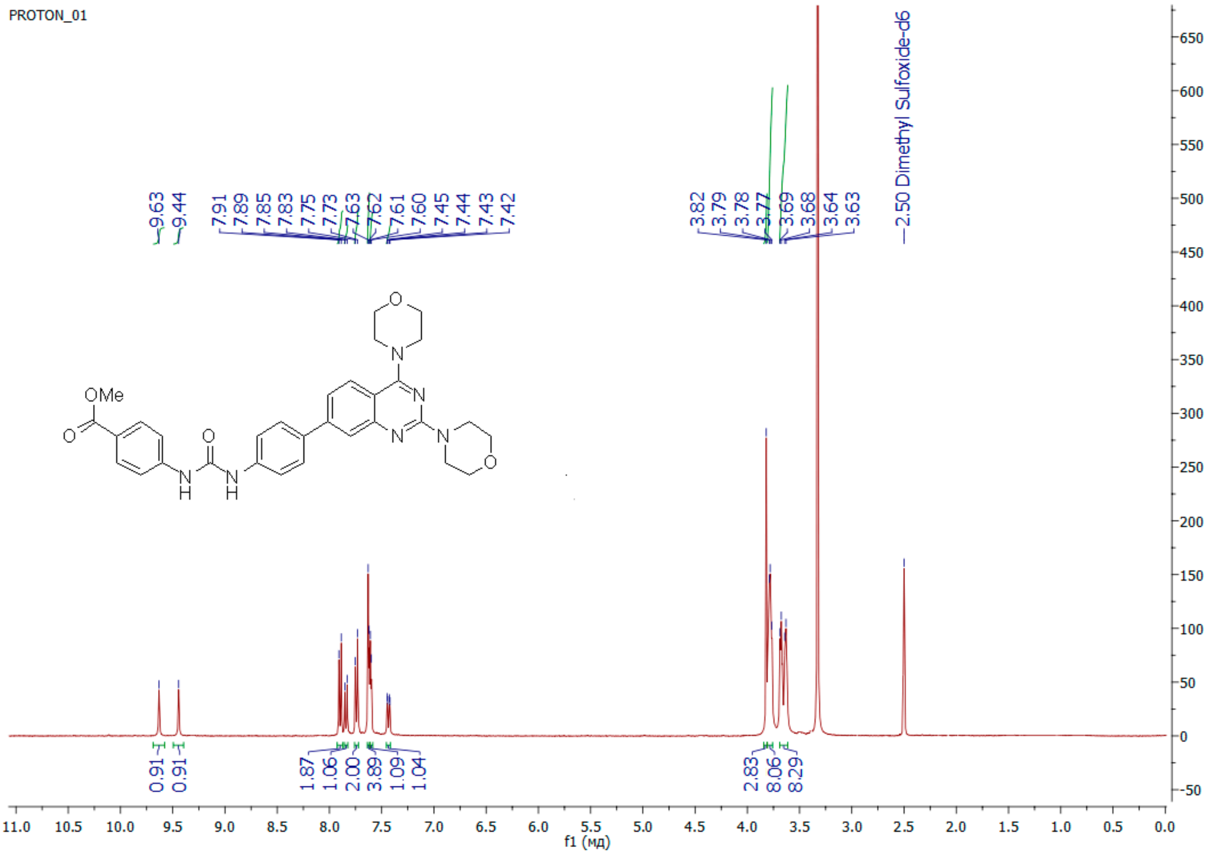

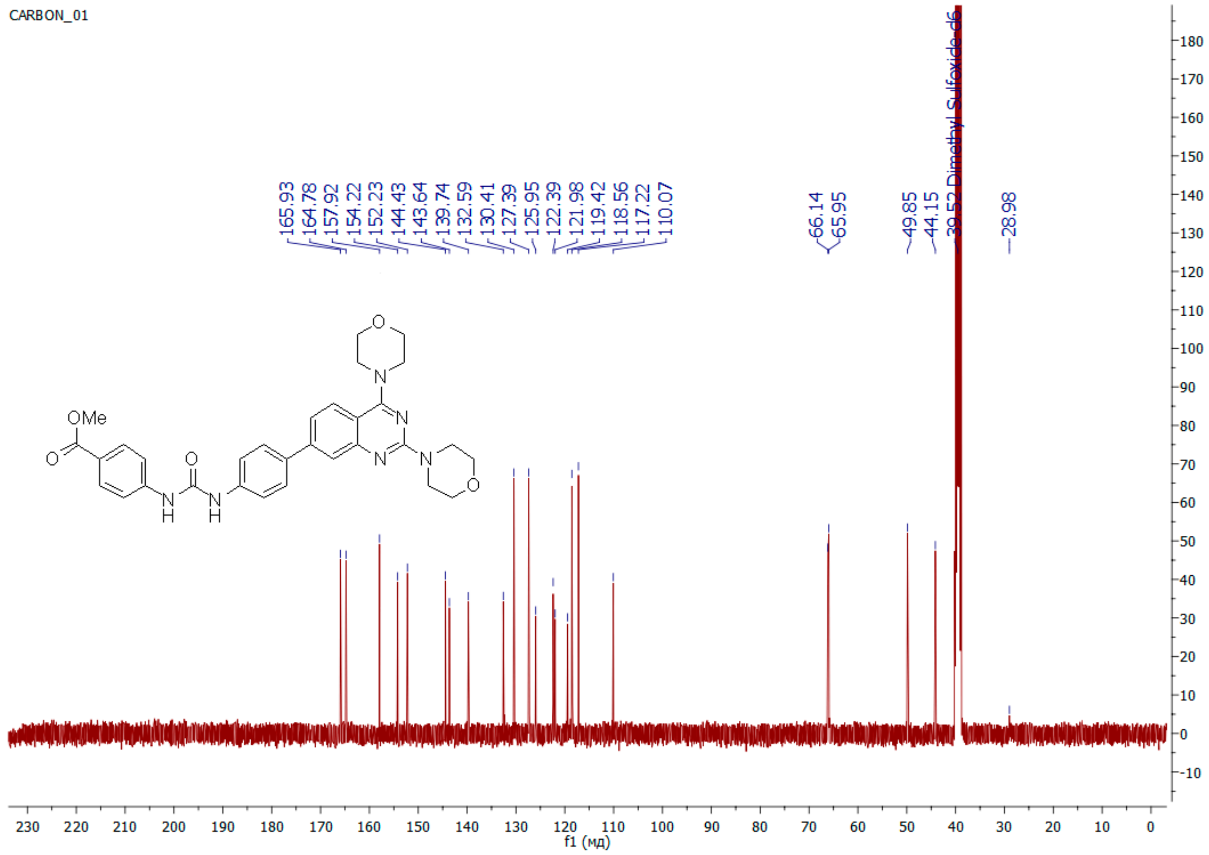

Ethyl 4-((4-(2,4-dimorpholinoquinazolin-7-yl)phenyl)carbamoyl)piperazine-1-carboxylate **7k**

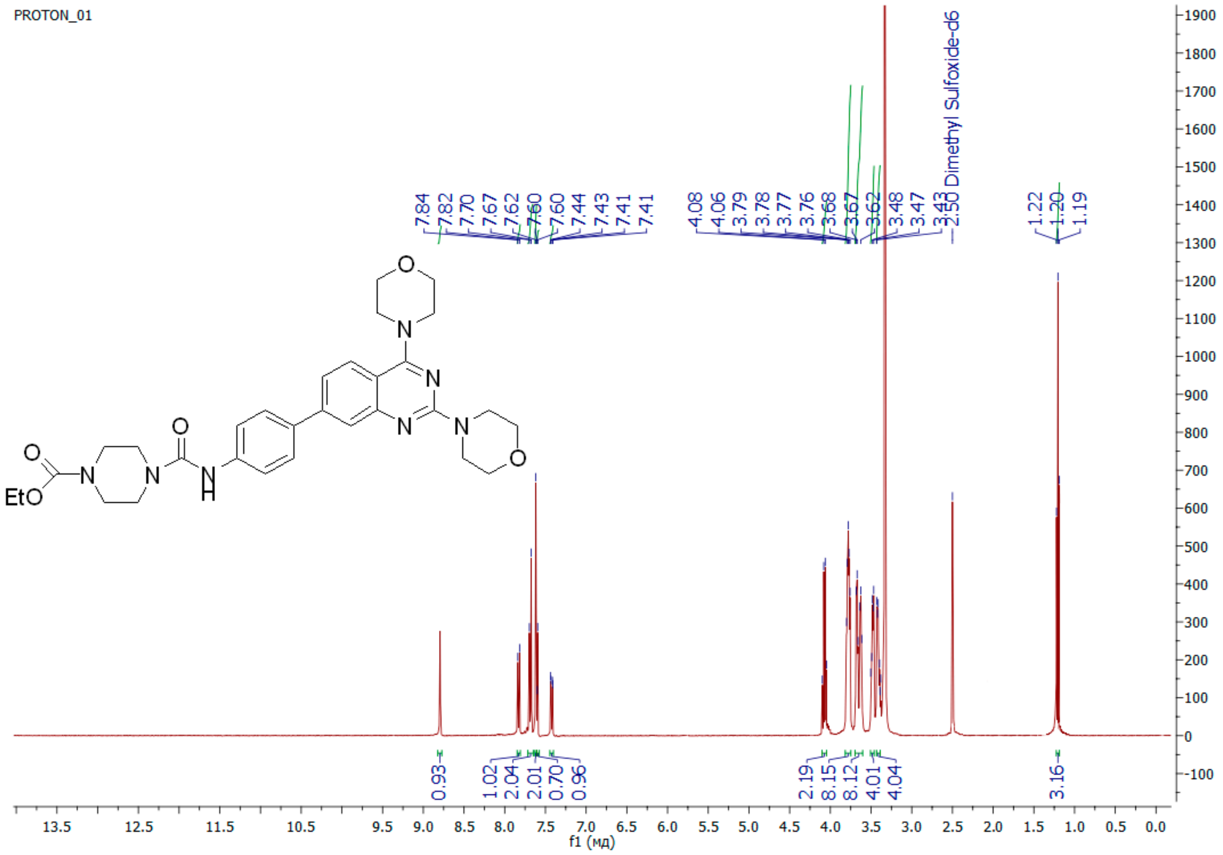

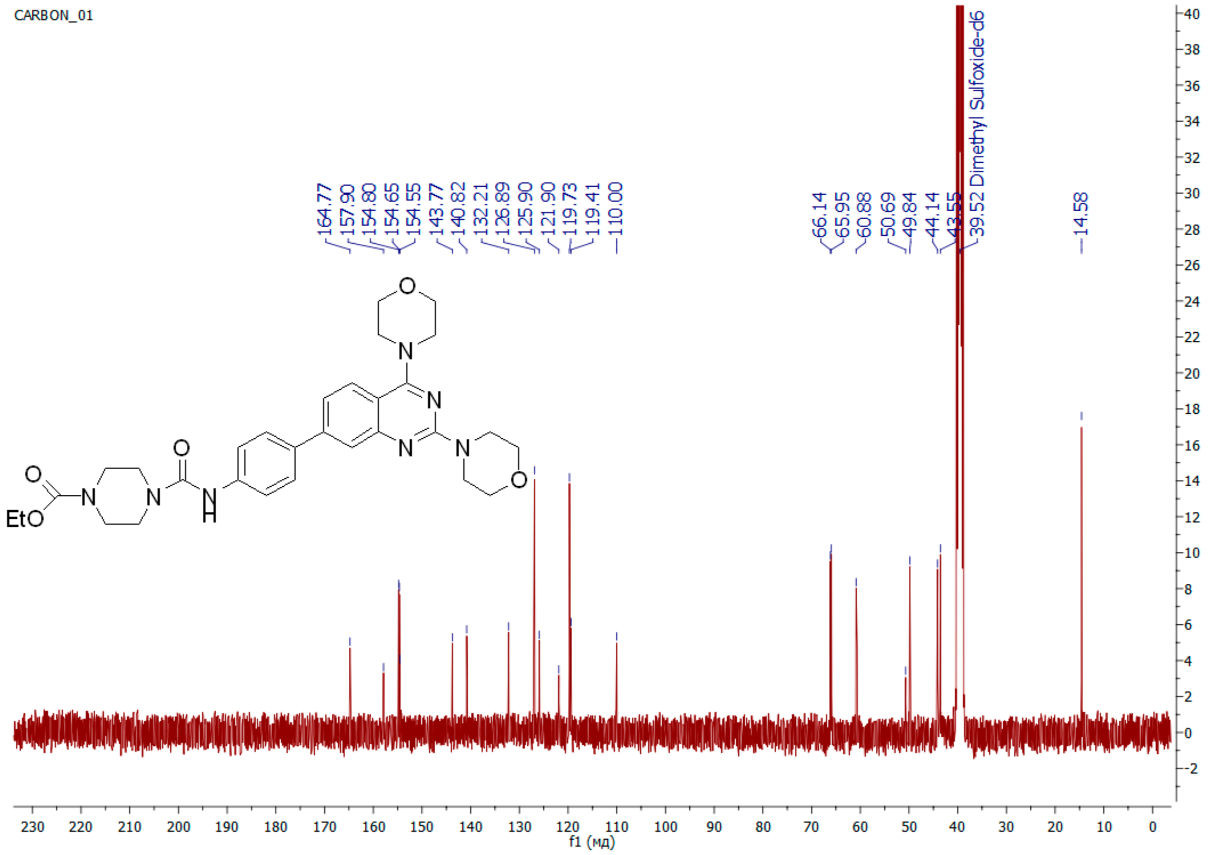

1-(4-(2,4-dimorpholinoquinazolin-7-yl)phenyl)-3-(prop-2-yn-1-yl)urea
7l

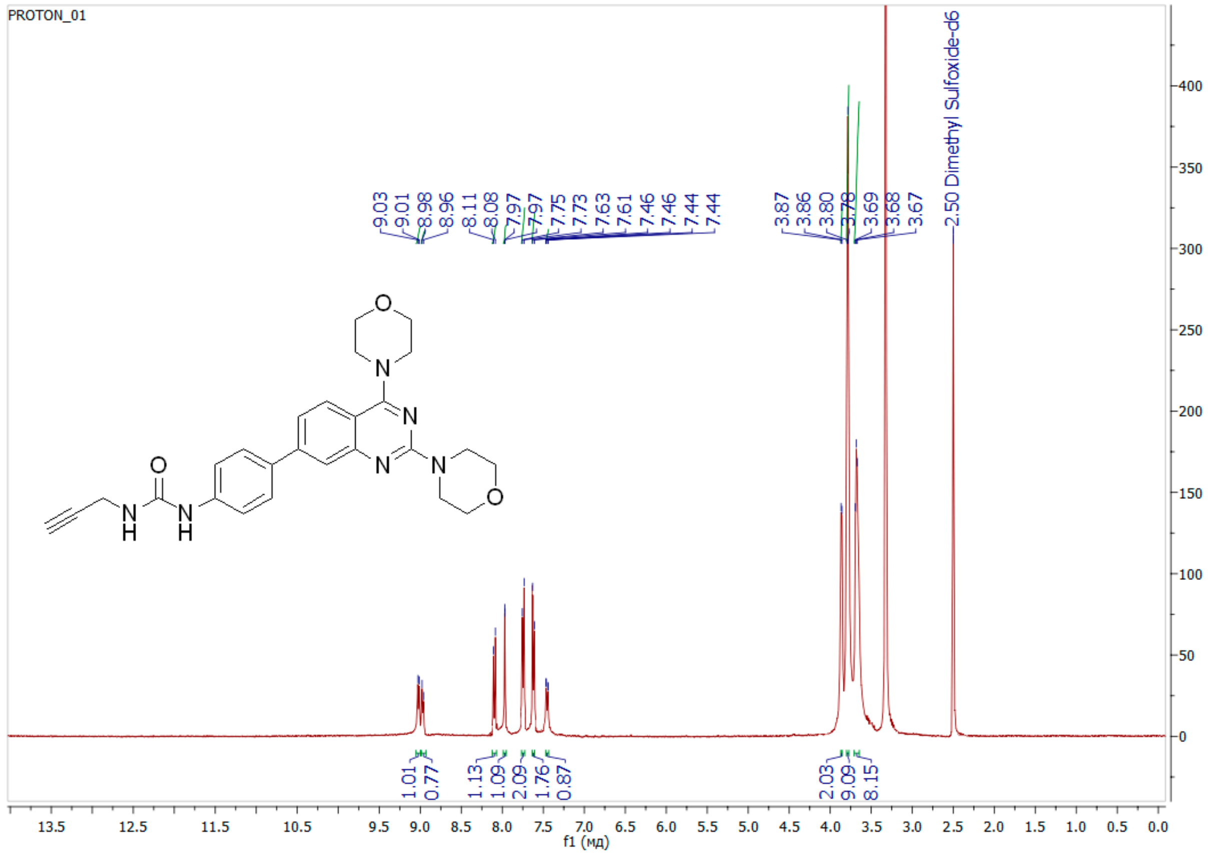

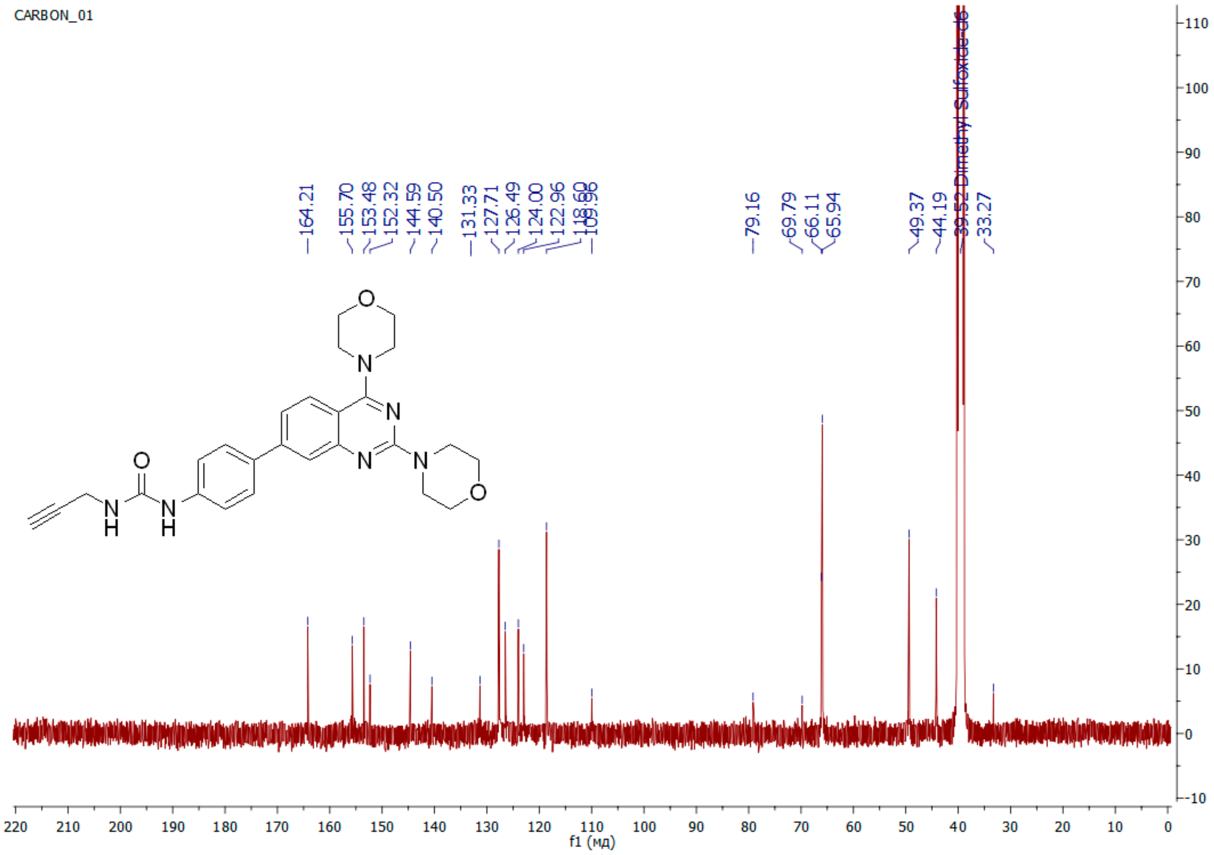

Table S1

Predicted ADME features of target compounds

Compound	c Log P	Solubility in water (ESOL)	Log Kp (skin permeation)	Number of H-bond donors	Number of H-bond acceptors
7a	2.90	9.00×10^{-3} mg/ml	-7.36 cm/s	2	6
7b	3.53	1.85×10^{-3} mg/ml	6.99 cm/s	2	6
7c	3.08	5.07×10^{-3} mg/ml	-7.31 cm/s	2	6
7d	2.74	1.57×10^{-2} mg/ml	7.14 cm/s	2	5
7e	1.22	1.08 mg/ml	-9.87 cm/s	4	8
7f	2.81	1.77×10^{-3} mg/ml	-7.05 cm/s	2	7
7g	3.84	1.35×10^{-3} mg/ml	-6.68 cm/s	2	6
7h	2.94	5.49×10^{-3} mg/ml	-7.18 cm/s	2	6
7i	4.45	1.58×10^{-4} mg/ml	-6.07 cm/s	2	5
7j	3.54	1.71×10^{-3} mg/ml	-7.11 cm/s	2	7
7k	2.48	1.12×10^{-2} mg/ml	-7.99 cm/s	1	7
7l	2.61	3.05×10^{-2} mg/ml	-7.44 cm/s	2	5

Docking of reference (line 1) and synthesized compounds (lines 2-5) in active site of PI3Kα and PI3Kγ isoforms. Structures of compounds are represented below.

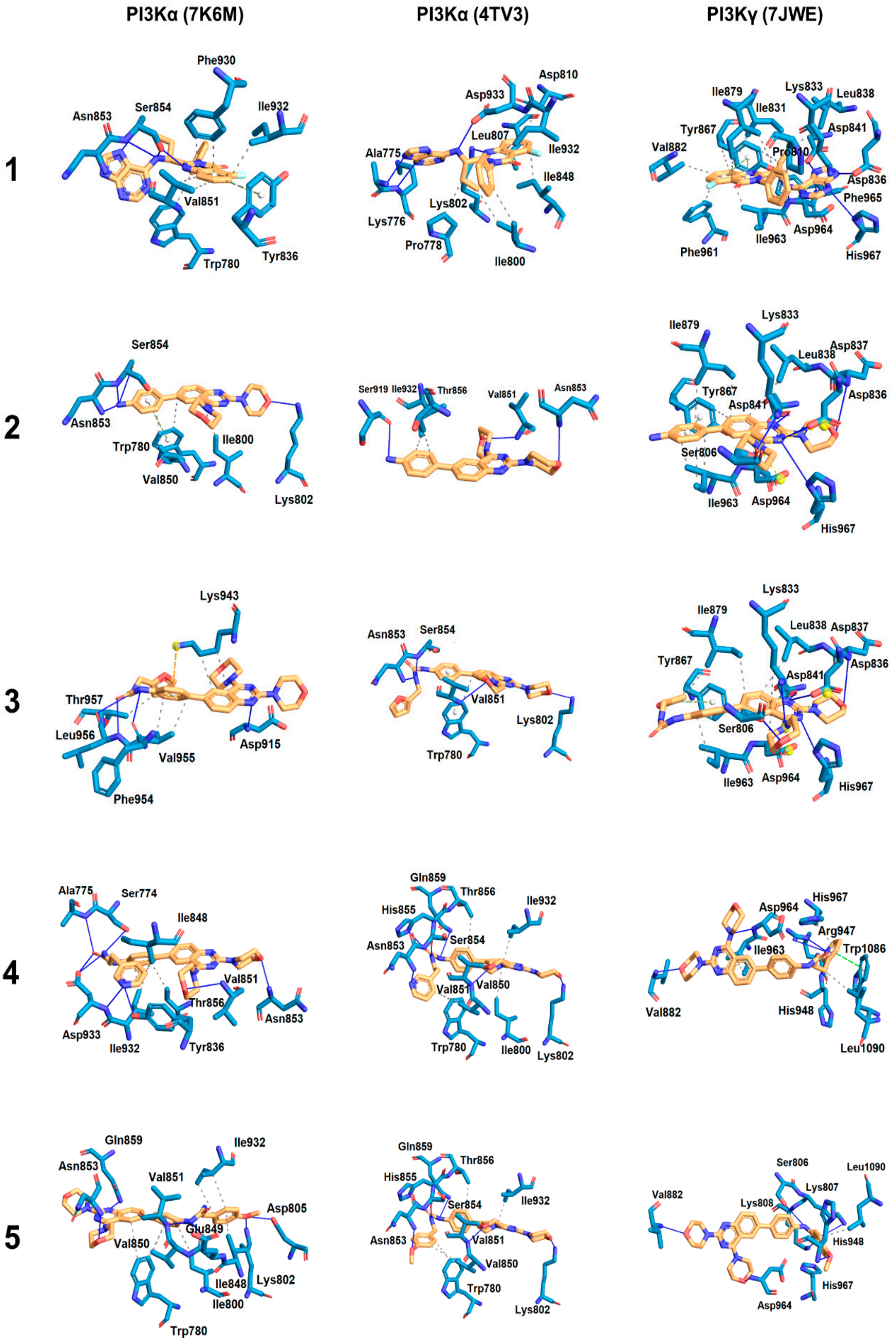

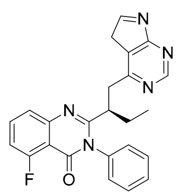

N
N
N
N
N
F
O

1

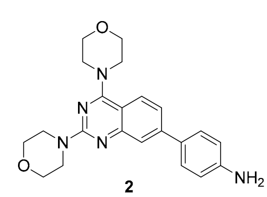

O
N
N
N
N
O
NH2

2

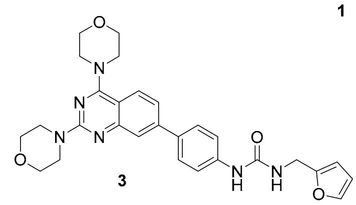

O
N
N
N
N
O
N
H
O
N
H
O

3

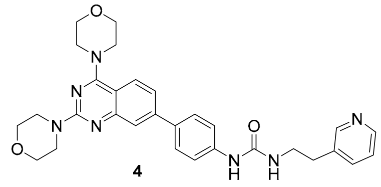

O
N
N
N
N
O
N
H
O
N
H
N

4

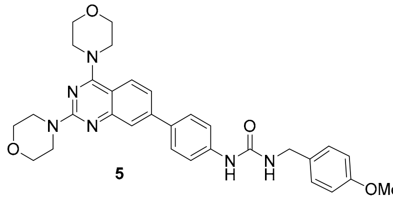

O
N
N
N
N
O
N
H
O
N
H
OMe

5

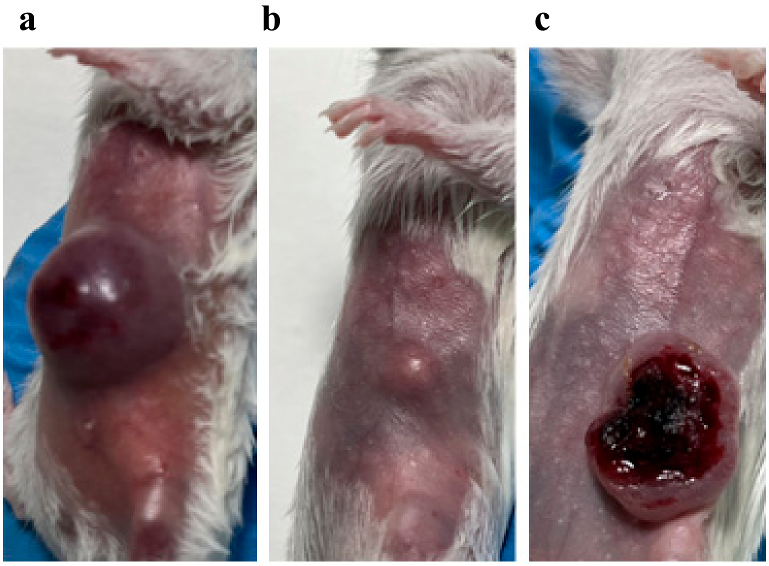

Figure S1. **Effect of the compound 7c on tumor growth**. BALB/c mice were s.c. inoculated with colorectal Colon-26 cells. a-c: Overview of the tumors in control (a), at early (b) and late (c) treatment at day 28 post tumor transplantation.

Area % Report

Data File: C:\EZChrom Elite\Enterprise\Projects\Default\Data\hplc.10001 20.06.2022 18-36-54.dat
Method: C:\EZChrom Elite\Enterprise\Projects\Default\Method\esk.met
Acquired: 20.06.2022 18:37:54
Printed: 21.06.2022 12:36:14

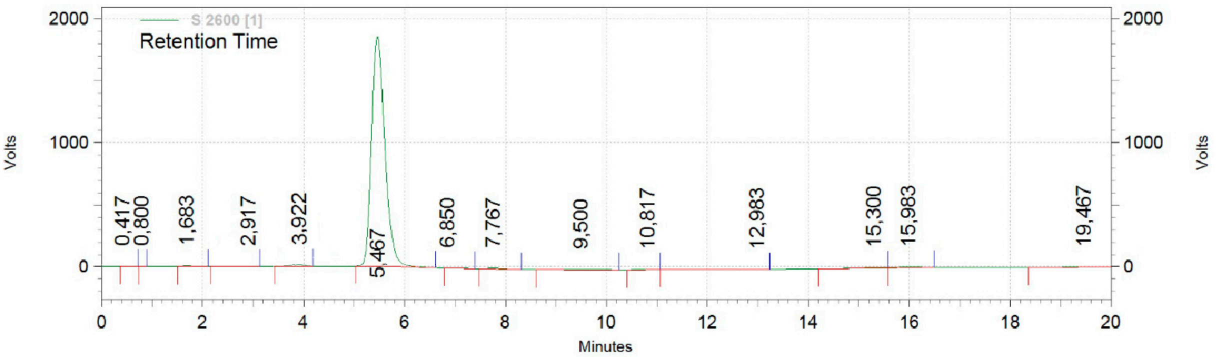

Retention time	Area	Area, %	Height	Height, %
0,417	916	0,00	46	0,00
0,800	1150	0,00	233	0,00
1,683	90433	0,18	8121	0,38
2,917	11892	0,03	414	0,01
3,922	539435	1,52	9047	0,73
5,467	33970229	95,89	1852504	97,45
6,850	33576	0,10	762	0,03
7,767	128543	0,34	6251	0,31
9,500	152964	0,42	3672	0,18
10,817	27283	0,08	1260	0,05
12,983	51771	0,15	724	0,03
15,300	164994	0,47	6506	0,31
15,983	211220	0,57	7880	0,35
19,467	88348	0,25	2518	0,12
**Total**	**35 420 983**	**100**	**1899938**	**100**
